# Benchmarking imputation methods on real-world clinical time series with simulated spatio-temporal missingness

**DOI:** 10.1038/s41467-026-77303-8

**Published:** 2026-10-05

**Authors:** Niklas Giesa, Rustam Zhumagambetov, Maria Sekutowicz, Sophie K. Piper, Victor Sikora, Felix Balzer, Stefan Haufe, Sebastian D. Boie

**Affiliations:** 1 Institute of Medical Informatics (IMI), Charité - Universitätsmedizin Berlin, corporate member of Freie Universität Berlin and Humboldt-Universität zu Berlin, Berlin, Germany; 2 Mathematical Modelling and Data Analysis Department, Physikalisch-Technische Bundesanstalt (PTB), Berlin, Germany; 3 Department of Anesthesiology and Intensive Care Medicine, Charité - Universitätsmedizin Berlin, corporate member of Freie Universität Berlin and Humboldt-Universität zu Berlin, Berlin, Germany; 4 Institute of Biometry and Clinical Epidemiology, Charité - Universitätsmedizin Berlin, corporate member of Freie Universität Berlin and Humboldt-Universität zu Berlin, Berlin, Germany; 5 Department of Infectious Diseases, Respiratory Medicine and Critical Care Medicine, Charité - Universitätsmedizin Berlin, corporate member of Freie Universität Berlin and Humboldt-Universität zu Berlin, Berlin, Germany; 6 Uncertainty, Inverse Modeling and Machine Learning Group, Technische Universität Berlin, Berlin, Germany

**Keywords:** Outcomes research, Data processing, Predictive medicine

## Abstract

Previous studies evaluating imputation methods on clinical time series do not jointly account for spatial (e.g., across features) and temporal (across time) structures in missingness. We present a simulation model that induces realistic spatio-temporal missingness in three real-world clinical datasets by sampling from Markov chains. A variety of imputation methods including last observation carried forward (LOCF), linear interpolation, and spatio-temporal autoencoder (STAE) are applied to fill these missingness patterns. Finally, we evaluate the influence of time series imputations on a downstream prediction task. Here we show that deep STAEs outperform simpler baseline methods when missingness patterns lack spatio-temporal structure. In contrast, linear interpolations perform best on spatio-temporal patterns, contributing to sufficient downstream performances. This study demonstrates that simple linear imputations attain robust performances for clinical real-world data with spatio-temporal missingness. We provide an open benchmark to evaluate the effect of missingness on clinical studies and prediction tasks.

## Abstract (web summary)

This work proposes a model to simulate complex missingness structures in clinical data. When applying various imputation methods to fill gaps of missing data, simple linear models are more robust than complex deep learning techniques.

## Introduction

Although routinely collected electronic health records (EHRs) offer opportunities for clinical machine learning (ML) or retrospective clinical studies, they present inherent challenges due to poor data quality^1–3^. Due to suboptimal fits, missingness in clinical time series hampers the application of ML^4–6^. Furthermore, it remains unclear whether current data handling strategies faithfully reconstruct patient trajectories.

Rubin^7^ introduced three missing data mechanisms: Missing completely at random (MCAR), where data with missing values form an arbitrary subset of the full data which is unobserved, missing at random (MAR), which occurs when the probability of a missing value depends on observed data, and missing not at random (MNAR), where the probability of a missing value depends on unobserved data. These patterns may also occur jointly in multivariate clinical time series^8^. As an example, MCAR may occur when a temperature sensor fails and randomly misses recordings. MAR can be caused by difficulties in measuring blood oxygen saturation for hypothermic patients^9,10^. Still, the exact reason for missing data in clinical time series remains unresolved.

To estimate missing clinical data, various imputation strategies have been established. As a popular example, multivariate imputations by chained equations (MICE) iteratively uses a series of conditional regression models to impute missing values^11^. While the originally proposed MICE algorithm^12^ treats observations as independent, some extensions additionally learn from temporal dependencies^13^. *K*-nearest neighbor (KNN) imputation uses available data from the most similar observed subjects, based on a defined similarity metric^14^. In 2020, Ma et al. proposed a multiple imputation denoising autoencoder (MIDA), which comprises stacked multi-layer perceptrons (MLPs) in an encoder-decoder architecture, reconstructing tabular MCAR data^15^. All these methods omit temporal dynamics, which are of particular interest to ML algorithms using time series of features^16^. Linear (LINPOL) or quadratic interpolation (QUAPOL), last observation carried forward (LOCF), or running median techniques are commonly used to easily impute time series. However, they lack the capability to capture spatial cross-feature dependencies^17–19^. Deep learning (DL) techniques, such as spatio-temporal denoising autoencoder (STAE)^20–22^, are claimed to overcome these issues by jointly learning temporal as well as spatial aspects across features, time, and between subjects.

Previous studies have applied imputation techniques on a wide range of clinical data modalities like EHRs^23^, omics^24^, electrocardiograph^22^, or electroencephalography^25^ recordings. Existing EHR imputation benchmarks often rely on the Medical Information Mart for Intensive Care (MIMIC), an openly accessible database from intensive care units (ICUs)^17,26–28^. Additionally, effects of the imputation quality on the performance of downstream classification tasks, like predicting acute kidney injury (AKI) or death, have been investigated^20,21,29^. While improved versions of KNN or MICE algorithms^13,14^ already addressed temporal aspects, other studies proposed the application of sequential DL autoencoder architectures.

Lia et al.^30^ and Li et al.^31^ proposed STAE models, based on long–short-term memory (LSTM) modules, which were enhanced by a temporal smoothing function. Other STAE models used combinations of convolutional neural networks (CNNs), gated-recurrent units (GRUs), and attention layers^26,32^ to impute both spatial and temporal missingness. Fan et al.^33^ proposed a CNN autoencoder architecture for processing time series via windowing, which involved a trainable graph for static attributes. This model was denoted as a spatio-temporal denoising graph autoencoder (STGAE). The authors Cao et al.^34^ designed an architecture, namely bidirectional recurrent imputation for time series (BRITS), which jointly learns imputations for multivariate time series and solves a downstream classification task. Cao et al. and Du et al.^35^ use masking of known values in time series that hold observed as well as unobserved data. The architecture self-attention-based imputation for time series (SAITS) by Due et al. relies on self-attention modules instead of recurrent mechanisms. In 2018, Chen et al.^36^ introduced the concept of neural ordinary differential equations (ODEs) that can be used to continuously model a latent space, improving discrete representations. The CTA (continuous-time autoencoder) model by Wi et al. exploits ODEs that are applied to regression coefficients from cubic splines, which were fitted to multivariate time series^37^.

A comprehensive evaluation by Jäger et al.^38^ demonstrated that MICE or KNN methods are favorable over DL methods for imputing tabular data. Similarly, Zamanzadeh et al.^20^ found that KNN outperformed STAE in more complex missing scenarios like MNAR. Results from additional studies raised the question of whether complex and highly tailored DL architectures translate into generalizable imputation methods^21,26,30^. Still, previous studies which evaluate STAE against mean or median imputation, MICE, or additional DL models did not consider LOCF or linear imputations^21–23,26,28,30^.

For simulating missing data, the majority of previous authors simply created synthesized or real-world complete case data (CCD), inducing MCAR under varying missing rates^15,17,21,22,25–27,30,32,39^. However, missingness patterns in clinical time series are characterized by spatio-temporal correlations which are not addressed by these MCAR inductions^40^. This study uses multivariate time series, which are described in the temporal dimension over time and in the spatial dimension across selected features. As an example, systolic and diastolic blood pressure (BP) values are likely either both present or absent at any time, since they rely on the same sensor and are measured simultaneously. These spatio-temporal relations have not been simulated before. To compare imputed data and CCD as ground truth (GT) data, the mean squared error (MSE) and the mean absolute error (MAE) are established as performance metrics^26,30,32^. However, these metrics are not suited to measure the faithful reproduction of the aforementioned spatio-temporal data correlations.

Related studies report heterogeneous results, which have not been systematically evaluated under realistic missingness patterns. Therefore, this study addresses these issues by introducing a benchmark for simulating realistic spatio-temporal missingness by sampling from Markov chains. We evaluate 20 different imputation methods, covering MICE, KNN, LOCF, and DL models, which are applied on commonly available vital signs from three datasets. Based on Li et al.^31^, we hypothesize that complex STAE outperforms common linear imputation techniques like LOCF. We further define a clinical downstream prediction task for each dataset, such as predicting postoperative delirium (POD), to assess the impact of imputations. Standard evaluations with MSE and MAE are complemented by assessing the reconstruction of spatio-temporal properties, offering a comprehensive benchmarking toolkit.

## Results

### Experimental setup

**Fig. 1:**
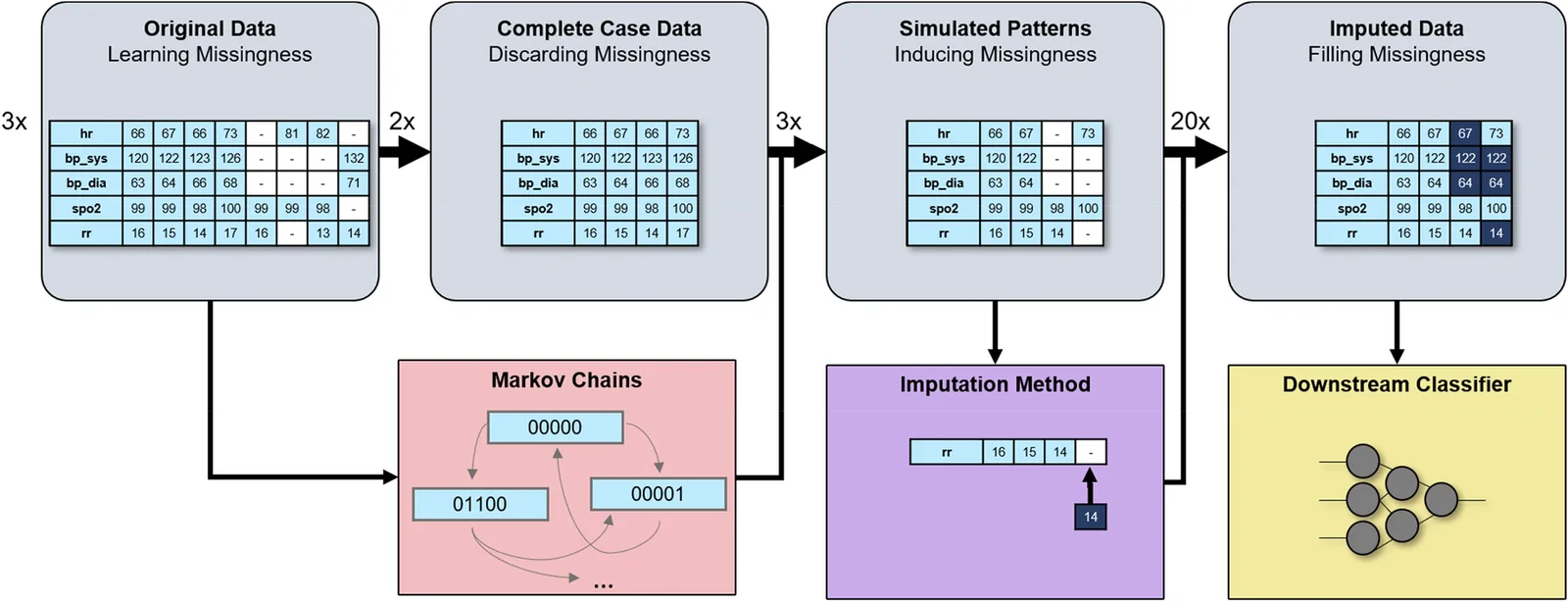
Experimental setup. We analyzed three datasets holding clinical time series as original data, applied two cleaning strategies for building complete case datasets (CCD), and simulated three types of missingness. We then applied twenty methods for imputing data and trained downstream classifiers, resulting in a total of 360 numerical experiments.

Figure 1 outlines our experimental setup. We estimated transition probabilities between consecutive states of spatio-temporal missingness from three original datasets. These datasets comprised the open datasets MIMIC and the high-resolution ICU dataset (HIRID)^41^, as well as the Intraoperative Care DElirium Prediction dataset (ICDEP), which was retrieved from the Charité medical center. We built simulation models for three different types of spatio-temporal missingness, which either reflected the original patterns or additionally amplified MAR or MNAR effects using Markov chains. After applying two types of data cleaning approaches, we discarded missing values to generate CCD for each dataset. Afterwards, we induced missingness patterns on CCD based on the fitted Markov chains, resulting in data with simulated missingness. The application of twenty different imputation methods led to a wide range of imputed datasets. Imputation methods covered simple mean-imputations, MICE, and more complex STAE models. Imputation performances were assessed with metrics using the known CCD as the GT. Among others, performance metrics included the cross-correlation error (CCorE), normalized root-mean squared error (NRMSE), and the normalized Wasserstein distance (NWSD). All methods are thoroughly explained in the Methods section. Finally, the imputed datasets were used to solve one clinical downstream classification task per dataset. Downstream performances were compared with respect to previously applied imputation methods.

### Missingness simulation

**Fig. 2:**
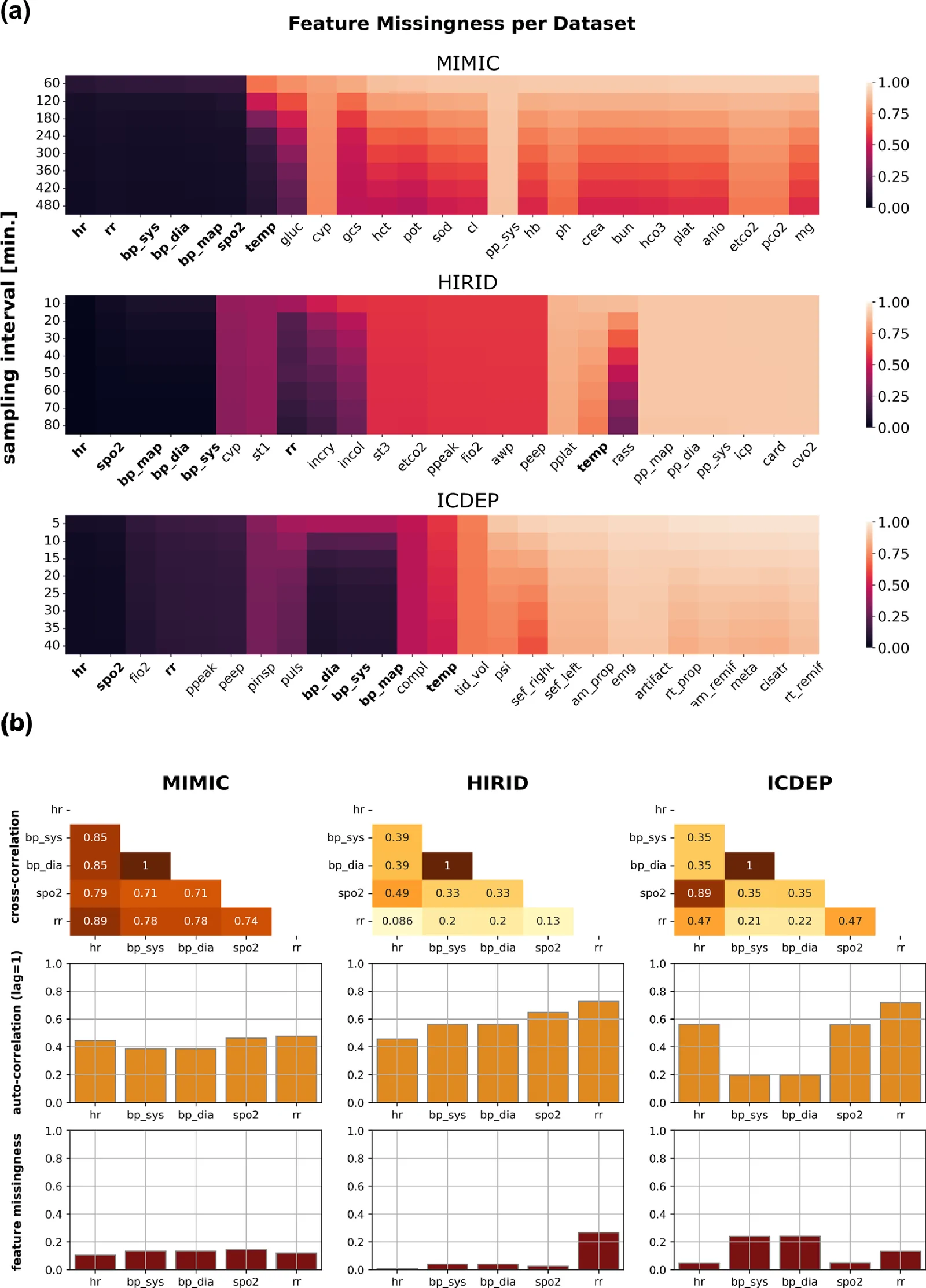
Feature missingness for varying sampling intervals and missing properties for resampled datasets. We included three datasets: Medical Information Mart for Intensiv Care (MIMIC), High-Resolution ICU Dataset (HIRID), Intraoperative Care DEElirium Prediction (ICDEP) data. Datasets were resampled with seven multiples of the original sampling interval of 60, 10, 5 min as {120, 180, …, 480},{20, 30, …, 80},{10, 15, …, 40} min for MIMIC, ICDEP, and HIRID, respectively. Panel **a** contains the top 25 features per dataset expressing the lowest feature missingness (FM) in the original data, sorted ascending (from left to right) by the mean FM across intervals. Features that are available in all three datasets are emphasized in bold. Panel **b** shows missingness properties in the form of cross- (lag = 0) and auto-correlation (lag = 1), which correlate binary missing indicators ${{{{\bf{R}}}}}_{n}$, as well as the FM. Properties are derived for the 60 min (originally) sampled MIMIC, 20 min resampled HIRID, and 10 min resampled ICDEP data for which our simulation model learned spatio-temporal missingness. The heart rate (hr), respiratory rate (rr), systolic, mean, and diastolic blood pressure (bp_sys, bp_map, bp_dia), blood oxygen saturation (spo2), and body temperature (temp) were identified as commonly available features across datasets. Source data are provided as a Source Data file.

To extract CCD from the original data, we searched for a set of common features that jointly examine low missingness (high availability). Time series were resampled with seven multiples 2, 3, . . . , 8× of the original sampling intervals (e.g., {120, 180, …, 480} min. for a 60 min. interval in MIMIC) to decrease their feature missingness (FM) rates (see Fig. 2a). Here, FM is defined as the fraction of unobserved values for one feature across all patient time series. Heart rate (h) and blood oxygen saturation (spo2) expressed low FM < 0.15 across all datasets without resampling. In contrast, the respiratory rate (rr) in HIRID (FM reduction from 0.403 at 10 min to 0.222 at 20 min) and the BP in ICDEP (FM reduction from 0.422 at 5 min to 0.218 at 10 min) benefited from 2× resampling, decreasing the missingness rate. However, temperature (temp) remained highly missing in some datasets (e.g., FM > 0.7 in HIRID for 80 min. sampling) even after 8 × resampling (see Table A.1 in Supplement A). Therefore, we continued our analysis without temp, due to high missingness, and without mean arterial BP (bp_mean), since this feature is a linear combination of systolic (bp_sys) and diastolic (bp_dia) BP.

We resampled HIRID and ICDEP in 20 min as well as 10 min intervals and used the 60 min sampled MIMIC data without resampling. The features hr, bp_sys, bp_dia, spo2, and rr are highly available and therefore selected. After correcting outliers, mean statistics marginally shifted while the standard-deviation (SD) decreased for most of the features (e.g., 86.36 ± 17.46 vs. 86.11 ± 16.80 bpm as mean ± SD for hr in HIRID) (see Table A.2 in Supplement A). In contrast to outlier corrections, we also removed outliers by setting them to missing, which changed spatio-temporal missingness patterns. We report these additional results in the Supplement A, because we aimed to simulate patterns closest to the original data (see Additional Results A.1).

In our resampled data, the cross-correlation (CCor) (lag = 0) of missingness was perfect (CCor = 1) between bp_sys and bp_dia across all datasets and ranged on higher levels in MIMIC (CCor > 0.71) than in other sets (see Fig. 2b). The auto-correlation (ACor) (lag = 1) was highest for rr in HIRID (ACor = 0.73) as well as in ICDEP for the same feature (ACor = 0.72). In contrast, BP features (ACor = 0.20 for bp_sys and bp_dia) showed decreased temporal correlations. FM rates were consistently low between features in MIMIC (FM = [0.10–0.12]). Measures of rr were increasingly missing in HIRID (FM = 0.27) while BP was more absent in ICDEP (FM = 0.24 for bp_sys and bp_dia) compared to other features (see Fig. 2b).

For the generation of CCD, resampled time series were reduced to blocks of at least three consecutive available values across all features. 14,286, 11,286, and 17,811 patients who complied with these conditions remained in MIMIC, HIRID, and ICDEP, respectively. Original patient characteristics were preserved (e.g., age of 64.1 ± 17.68 vs. 63.88 ± 17.59 years in MIMIC) while the lengths of sequences were reduced (e.g. 250.75 ± 456.49 vs. 129.35 ± 191.04 for HIRID) (see Table 1 and Fig. A.1 in Supplement A).

**Table 1:** Patient characteristics for original - and complete case data

	MIMIC		HIRID		ICDEP	
	Original data	CCD	Original data	CCD	Original data	CCD
#Patients	34,472	14,286	36,098	11,286	41,832	17,811
Sampling interval	1 h	1 h	10 min	20 min	5 min	10 min
Sequence length	57.21 ± 45.19	21.32 ± 19.64	250.75 ± 456.49	129.35 ± 191.04	14.06 ± 7.39	9.48 ± 3.71
age [years]	64.1 ± 17.68	63.88 ± 17.59	63.52 ± 15.20	64.24 ± 14.74	53.45 ± 18.47	54.25 ± 18.11
In-hospital death (%)	9.58	10.44	6.78	9.98	1.29	1.32
sex [F:female, M:male]	43.47% F 56.53% M	44.46% F 55.54% M	35.80% F 64.20% M	31.46% F 68.54% M	46.56% F 53.44% M	49.76% F 50.24% M

The number of patients (#patients), the sampling interval, and further statistics are provided per dataset for the original data and the complete case data (CCD) after resampling and removing missingness. Data were cleaned so that the original spatio-temporal aspects are preserved. Numerical statistics are reported as mean ± standard-deviation. We included three datasets: Medical Information Mart for Intensive Care (MIMIC), High-Resolution ICU Dataset (HIRID), Intraoperative Care DElirium Prediction (ICDEP) data.

**Fig. 3:**
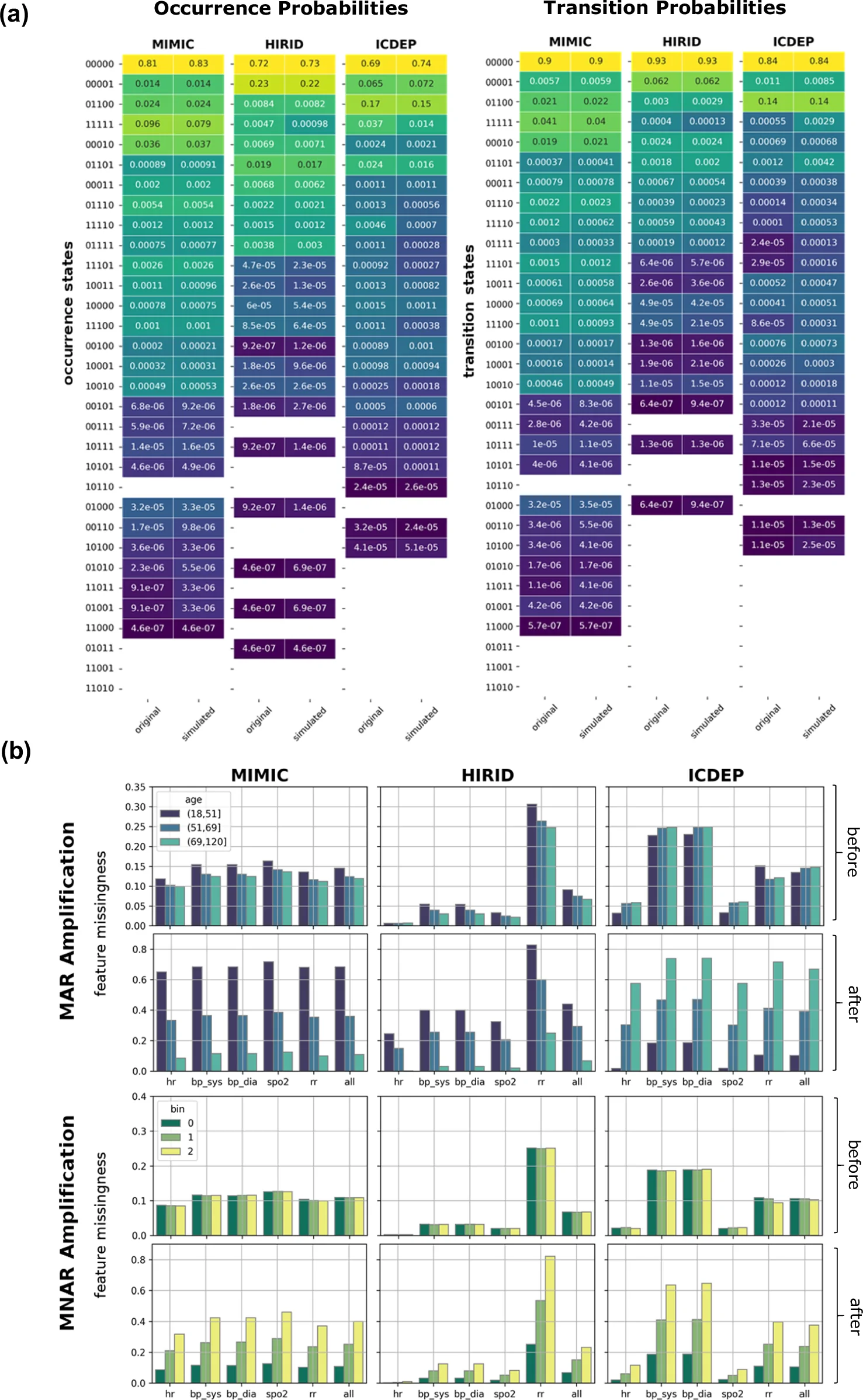
Occurrence and transition probabilities learned by a Markov chain simulation model, amplification of missing at random and missing not at random effects. We included three datasets: Medical Information Mart for Intensive Care (MIMIC), High-Resolution ICU Dataset (HIRID), and Intraoperative Care DEElirium Prediction (ICDEP) data. In panel **a**, we display the occurrence probabilities of 32 states encoding if one of the five features (hr: heart rate, bp_sys: systolic blood pressure, bp_dia: diastolic blood pressure, spo2: blood oxygen saturation, rr: respiratory rate) is missing (1) or observed (0) at one time step. In addition, transition probabilities are displayed from one state to a consecutive one (as From_To) for the top 32 most frequent transitions. Probabilities are displayed for the original resampled data (original) and the data with simulated missingness (simulated) to visualize the quality of our simulation model. In panel **b**, the feature missingness (per age or value bin) before and after missing at random (MAR) or missing not at random (MNAR) amplification is shown for each feature as well as across all features. In MAR, missingness depends on observed data (here, age). In MNAR, missingness depends on unobserved data, here the ground truth (GT) values of vital signs, which are not known after missing induction. Equally sized age bins are used in the MAR setup. Value edges for bins (0, 1, 2 in MNAR setting) are included in Table A.4 in Supplement A. Source data are provided as a Source Data file.

To induce missingness in CCD, a Markov chain simulation model learned occurrence and transition probabilities for the five selected features. The state space covered 2^5^ = 32 possible states, where each state vector of length 5 indicated if one of each feature was present (0) or absent (1) at a single time step. For each state, binary indicators were concatenated in the order of hr, bp_sys, bp_dia, spo2, rr. We observed the actual occurrence of 28, 23, and 24 states for MIMIC, HIRID, and ICDEP, respectively (see Fig. 3a). The total present state (TPS) (**s** = [0, 0, 0, 0, 0]), encoding that all features were available, was most frequent (*f* > 0.70) across all original datasets while the isolated absence of rr measures (**s** = [0, 0, 0, 0, 1]) was occurring predominantly in HIRID (*f* = 0.23) than in other sets (*f* < 0.07). The simultaneous missingness of bp_dia and bp_sys alone (**s** = [0, 1, 1, 0, 0]) occurred more often in ICDEP (*f* = 0.17) than in MIMIC or HIRID (*f* < 0.024). The transition from one TPS to another TPS was most frequent in all datasets (*f* > 0.55). The transitions from the TPS to the simultaneous absence of bp_sys and bp_dia values and vice versa were about equally frequent in ICDEP (*f* = 0.11, 0.12) and occurred rarely in other datasets (*f* < 0.018). We found minor differences in the occurrence and transition probabilities (frequencies) between original and simulated missingness (see Fig. 3a). We further detected simulated missingness patterns that were similar to patterns presented by the original resampled data (see Fig. A.2, Table A.3 in Supplement A). The absolute frequencies of occurrence states were retrieved and normalized globally for the entire population across all time series per dataset, resulting in a set of occurrence probabilities.

The absolute count of transitions from every single state to all other possible states was retrieved and normalized so that the resulting relative frequencies, which translate to transition probabilities, sum up to one. Consequently, the Markov chain model uses these normalized transition probabilities to sample the next consecutive state (at *t* + 1) for the selected state (at *t*).

When analyzing existing MAR effects of original ICU data from MIMIC and HIRID, we observed that clinical parameters were more absent for younger (age bin [18,51] years: FM = 0.15, 0.09 in MIMIC, HIRID) than for elderly patients (age bin (69,120] years: FM = 0.12, 0.07 in MIMIC, HIRID) across all features (see Fig. 3b). In ICDEP, we detected a reversed effect (FM = 0.15, 0.13 for age bins [18,51], (69,120] years). After the multiplication of occurrence and transition probabilities with factors according to three equally sized age bins (see the “Methods” section), we amplified these effects with our simulation model (age bin [18,51] years: FM = 0.15,0.64 before and after MAR amplification in MIMIC for all features). We found no ad-hoc association of GT feature values and the simulated missingness by our Markov model, which was also initially not conditioned on these relations. Thus, we simply assumed a positive relationship between GT value levels and their missingness to amplify MNAR effects (FM = 0.11, 0.60 for the value bin 0 before and after amplification) (see Fig. 3b). We also show further amplifications on simulated occurrence states (see Fig. A.3 in Supplement A).

### Imputation benchmark

After simulating spatio-temporal missingness on CCD, we applied 20 imputation methods comprising averaging techniques like running median (RMEDI), interpolation methods like linear interpolation, extrapolation methods like LOCF, MICE (e.g., MICE using tree-based estimations, defined as MICTRE), and DL architectures like bidirectional LSTM autoencoder (BILSTAE) (see the “Methods” section). We initially conducted our analysis with no amplification for MAR and MNAR. Imputation techniques were categorized into time-causal and non-time-causal methods, with the latter having the advantage of accessing future data points (see Table 2). Time-causal methods are suitable for an online prediction setting, whereas non-time-causal methods offer increased performance for retrospective studies.

**Table 2:** Performance of 20 different imputation methods on spatio-temporal missingness

	MIMIC			HIRID			ICDEP		
Name	${{{{\rm{CCorE}}}}}_{loc}\downarrow$	NRMSE_loc_*↓*	NWSD_glob_*↓*	${{{{\rm{CCorE}}}}}_{{\rm {loc}}}\downarrow$	NRMSE_loc_*↓*	NWSD_glob_*↓*	${{{{\rm{CCorE}}}}}_{{\rm {loc}}}\downarrow$	NRMSE_loc_*↓*	NWSD_glob_*↓*
*Non-time-causal methods*									
PMEAN	9.769 ± 0.195	16.256 ± 0.135	0.529 ± 0.105	6.610 ± 0.105	43.133 ± 0.596	0.379 ± 0.334	8.789 ± 0.155	15.561 ± 0.128	0.438 ± 0.216
PMEDI	9.666 ± 0.192	15.967 ± 0.131	0.527 ± 0.105	4.893 ± 0.091	27.152 ± 0.305	0.322 ± 0.272	8.388 ± 0.151	15.359 ± 0.130	0.432 ± 0.213
LINPOL	6.581 ± 0.164	10.578 ± 0.103	0.090 ± 0.016	3.384 ± 0.090	17.824 ± 0.296	0.026 ± 0.024	7.555 ± 0.156	12.539 ± 0.128	0.184 ± 0.069
NEAPOL	7.383 ± 0.175	12.280 ± 0.116	0.106 ± 0.019	3.596 ± 0.093	20.892 ± 0.337	0.021 ± 0.019	8.108 ± 0.156	14.244 ± 0.135	0.215 ± 0.078
QUAPOL	8.060 ± 0.179	13.429 ± 0.132	0.096 ± 0.013	4.726 ± 0.097	30.984 ± 0.567	0.218 ± 0.246	8.012 ± 0.157	13.951 ± 0.135	0.197 ± 0.072
MICTRE	12.738 ± 0.211	23.687 ± 0.184	0.014 ± 0.003	8.288 ± 0.115	94.193 ± 1.828	0.008 ± 0.007	12.211 ± 0.184	22.102 ± 0.182	0.017 ± 0.005
MICKNN	10.048 ± 0.198	16.641 ± 0.135	0.471 ± 0.104	6.060 ± 0.103	47.676 ± 0.777	0.145 ± 0.115	9.484 ± 0.170	16.223 ± 0.135	0.301 ± 0.143
SAE	9.858 ± 0.198	16.251 ± 0.135	0.529 ± 0.105	6.597 ± 0.105	43.020 ± 0.595	0.379 ± 0.333	8.769 ± 0.154	15.578 ± 0.128	0.438 ± 0.216
MIDA	9.846 ± 0.197	16.265 ± 0.135	0.518 ± 0.103	6.662 ± 0.107	43.127 ± 0.596	0.378 ± 0.333	9.250 ± 0.164	15.538 ± 0.128	0.436 ± 0.215
BILSTAE	6.581 ± 0.159	10.840 ± 0.097	0.234 ± 0.041	3.348 ± 0.091	18.685 ± 0.364	0.337 ± 0.385	8.166 ± 0.156	13.040 ± 0.119	0.281 ± 0.132
*Time-causal methods*									
1MEAN	9.852 ± 0.195	16.695 ± 0.139	0.541 ± 0.112	4.719 ± 0.092	26.046 ± 0.276	0.333 ± 0.281	9.064 ± 0.153	17.423 ± 0.133	0.491 ± 0.242
1MEDI	9.696 ± 0.192	16.333 ± 0.134	0.535 ± 0.108	4.618 ± 0.093	25.769 ± 0.277	0.338 ± 0.285	8.735 ± 0.149	16.783 ± 0.131	0.472 ± 0.228
RMEAN	6.663 ± 0.162	11.798 ± 0.104	0.131 ± 0.023	3.370 ± 0.079	19.542 ± 0.233	0.116 ± 0.112	7.945 ± 0.152	14.870 ± 0.134	0.210 ± 0.075
RMEDI	6.692 ± 0.162	11.873 ± 0.107	0.117 ± 0.020	3.617 ± 0.082	20.406 ± 0.256	0.141 ± 0.144	7.970 ± 0.153	14.815 ± 0.136	0.196 ± 0.067
MICET	12.739 ± 0.211	9.283 ± 0.081	0.032 ± 0.010	6.600 ± 0.108	17.280 ± 0.242	0.160 ± 0.172	12.371 ± 0.184	11.825 ± 0.130	0.051 ± 0.020
LOCF	7.041 ± 0.164	11.862 ± 0.114	0.062 ± 0.012	3.164 ± 0.081	17.080 ± 0.242	0.010 ± 0.008	7.764 ± 0.154	14.247 ± 0.139	0.161 ± 0.050
LINEXT	9.390 ± 0.181	17.145 ± 0.173	0.131 ± 0.023	4.188 ± 0.090	25.686 ± 0.399	0.137 ± 0.148	8.609 ± 0.157	18.455 ± 0.180	0.182 ± 0.066
QUAEXT	13.658 ± 0.223	29.242 ± 0.331	0.459 ± 0.092	5.870 ± 0.101	46.389 ± 0.796	0.393 ± 0.423	9.898 ± 0.164	25.231 ± 0.288	0.324 ± 0.163
LSTAE	7.107 ± 0.168	11.775 ± 0.105	0.265 ± 0.045	4.588 ± 0.096	23.791 ± 0.317	0.177 ± 0.168	8.553 ± 0.161	13.877 ± 0.122	0.319 ± 0.146
STGAE	9.843 ± 0.194	16.544 ± 0.138	0.454 ± 0.096	5.892 ± 0.100	35.975 ± 0.471	0.452 ± 0.439	9.125 ± 0.159	16.225 ± 0.135	0.417 ± 0.209
*Method fit with static features*									
MICTRE	12.738 ± 0.211	23.687 ± 0.184	0.014 ± 0.003	8.288 ± 0.115	94.193 ± 1.828	0.008 ± 0.007	12.211 ± 0.184	22.102 ± 0.182	0.017 ± 0.005
MICKNN	10.629 ± 0.208	17.368 ± 0.144	0.014 ± 0.003	6.055 ± 0.104	47.294 ± 0.770	0.144 ± 0.116	9.429 ± 0.169	16.183 ± 0.135	0.282 ± 0.150
MICET	11.394 ± 0.210	9.134 ± 0.082	0.033 ± 0.010	6.621 ± 0.108	17.278 ± 0.242	0.161 ± 0.170	12.370 ± 0.182	11.753 ± 0.130	0.052 ± 0.021
SAE	9.843 ± 0.197	16.248 ± 0.135	0.529 ± 0.105	6.670 ± 0.107	43.110 ± 0.596	0.382 ± 0.338	8.756 ± 0.154	15.545 ± 0.128	0.437 ± 0.215
MIDA	9.833 ± 0.196	16.263 ± 0.135	0.522 ± 0.104	6.659 ± 0.106	43.817 ± 0.609	0.377 ± 0.332	9.220 ± 0.163	15.563 ± 0.128	0.432 ± 0.213
BILSTAE	6.442 ± 0.156	10.765 ± 0.096	0.216 ± 0.036	4.548 ± 0.094	24.204 ± 0.335	0.183 ± 0.175	8.683 ± 0.160	13.862 ± 0.124	0.298 ± 0.150
LSTAE	7.278 ± 0.170	12.010 ± 0.106	0.269 ± 0.047	4.783 ± 0.096	25.560 ± 0.338	0.224 ± 0.214	8.302 ± 0.156	13.699 ± 0.121	0.310 ± 0.146

Averaging methods, including imputation with a population mean or median (PMEAN/PMEDI), with running means or medians (RMEAN/RMEDI) or with means and medians from the first time step (1MEAN/1MEDI), are tested. Linear, nearest, and quadratic interpolation (LINPOL, NEAPOL, QUAPOL) as well as linear and quadratic extrapolation methods (LINEXT, QUAEXT) are included. Multivariate imputations by chained equations (MICE) are configured with tree, k-nearest neighbor, or a temporal component (MICTRE, MICKNN, MICET). More complex denoising autoencoder comprise stacked autoencoder (SAE), multiple denoising autoencoder (MIDA), (bidirectional) LSTM autoencoder as (BI-)LSTAE, and spatio-temporal denoising graph autoencoder (STGAE) (see Table 3). Methods were either non-time-causal, consuming time points from the future, or time-causal, being limited to previous measurements only. As a separate analysis, we investigated the change in imputation performance when fitting methods with static attributes like age. All spatio-temporal autoencoders such as (BI) LSTAE or STGAE were trained on LOCF pre-imputed ground truth (GT) values. Cross-correlation errors (${{{{\rm{CCorE}}}}}_{{\rm {loc}}}$) and the normalized root mean squared error (NRMSE_loc_) were calculated per time series (locally), the normalized Wasserstein distance (NWSD_glob_) was calculated across all measurements and time series (globally). We included three datasets: Medical Information Mart for Intensive Care (MIMIC), High-Resolution ICU Dataset (HIRID), Intraoperative Care DElirium Prediction (ICDEP) data. We report all errors in % with mean ± 95%CI across time series (14,286, 11,286, and 17,811 for MIMIC, HIRID, ICDEP), 5 features, and 3 validation folds.

Among non-time-causal methods, LINPOL achieved the smallest local NRMSE across all datasets (NRMSE_loc_ = 10.578 ± 0.103, 17.824 ± 0.296, 12.539 ± 0.128 as mean ± 95%CI for MIMIC, HIRID, ICDEP, calculated in %). The method also resulted in the smallest local CCorE in MIMIC (${{{{\rm{CCorE}}}}}_{{\rm {loc}}}$= 6.581 ± 0.164) and ICDEP (${{{{\rm{CCorE}}}}}_{{\rm {loc}}}$= 7.555 ± 0.156). BILSTAE yielded similar errors in MIMIC (${{{{\rm{CCorE}}}}}_{{\rm {loc}}}$= 6.581 ± 0.159) and was distinctly beneficial for reconstructing cross-correlations in HIRID (${{{{\rm{CCorE}}}}}_{loc}$= 3.348 ± 0.091) (see Table 2). MICE yielded the lowest globally computed NWSD using tree estimators (NWSD_glob_ = 0.014 ± 0.003, 0.010 ± 0.007, 0.017 ± 0.005 for MIMIC, HIRID, ICDEP).

Among time-causal methods, LOCF imputations yielded the lowest error margins in HIRID (${{{{\rm{CCorE}}}}}_{loc}$= 3.164 ± 0.081, NRMSE_loc_ = 17.280 ± 0.242, NWSD_glob_ = 0.010 ± 0.008) compared to other imputation methods (see Table 2). The LSTM autoencoder LSTAE resulted in smaller NRMSE in contrast to LOCF for MIMIC (NRMSE_loc_ = 11.775 ± 0.105) as well as ICDEP (NRMSE_loc_ = 13.877 ± 0.122). However, temporal multivariate imputations by chained equations (MICET), which was implemented as a time-causal version of MICE, resulted in significantly lower error margins for these two datasets (NRMSE_loc_ = 9.283 ± 0.081, 11.825 ± 0.130 in MIMIC, ICDEP). Running mean imputation (RMEAN) was particularly preserving cross-correlations in MIMIC (${{{{\rm{CCorE}}}}}_{{\rm {loc}}}$: 6.663 ± 0.162). Non-time-causal models yielded overall better performances than time-causal ones. LOCF and linear imputation resulted in overall small errors across additional performance metrics (see Table A.5 in Supplement A).

We aimed to improve imputations by fitting methods on static data (age, gender) alongside vital sign time series. In MIMIC, the autoencoder BILSTAE that was trained with static attributes could improve the preservation of cross-correlations (*CCorD*_loc_ = 6.442 ± 0.156) (see Table 2). We did not see a pronounced improvement in the NWSD for MICE imputations. Further ablations of trained STAE are integrated in the Supplement A (see Table A.6 and Additional Results A.2). Here, we note that performances varied across ablation parameters without highlighting a single one that boosted performances across all datasets.

While LINPOL achieved the overall lowest local RMSE scores across all datasets for methods that can access future points in time (10.578 ± 0.103, 17.824 ± 0.296, 2.539 ± 0.128 for MIMIC, HIRID, ICDEP), different rankings were observed for time-causal imputers. In MIMIC, MICET yielded the smallest RMSE of 9.283 ± 0.081, followed by the LSTAE with a RMSE of 11.775 ± 0.105. In contrast, linear LOCF imputations (RMSE of 17.280 ± 0.242) as well as RMEAN (RMSE of 19.542 ± 0.233) and RMEDI (RMSE of 20.406 ± 0.256) methods were better suited for HIRID than DL imputers. In ICDEP, the methods MICET, LSTAE, and LOCF yielded the lowest RMSE of 11.825 ± 0.130, 13.877 ± 0.122, and 14.247 ± 0.139. Similar performance results were found for amplified MAR and MNAR effects (see Tables A.7 and A.8 in Supplement A). We highly suggest considering additional performance scores (e.g., CCorD, NWSD) to fully assess the imputation quality of benchmarked methods.

**Fig. 4:**
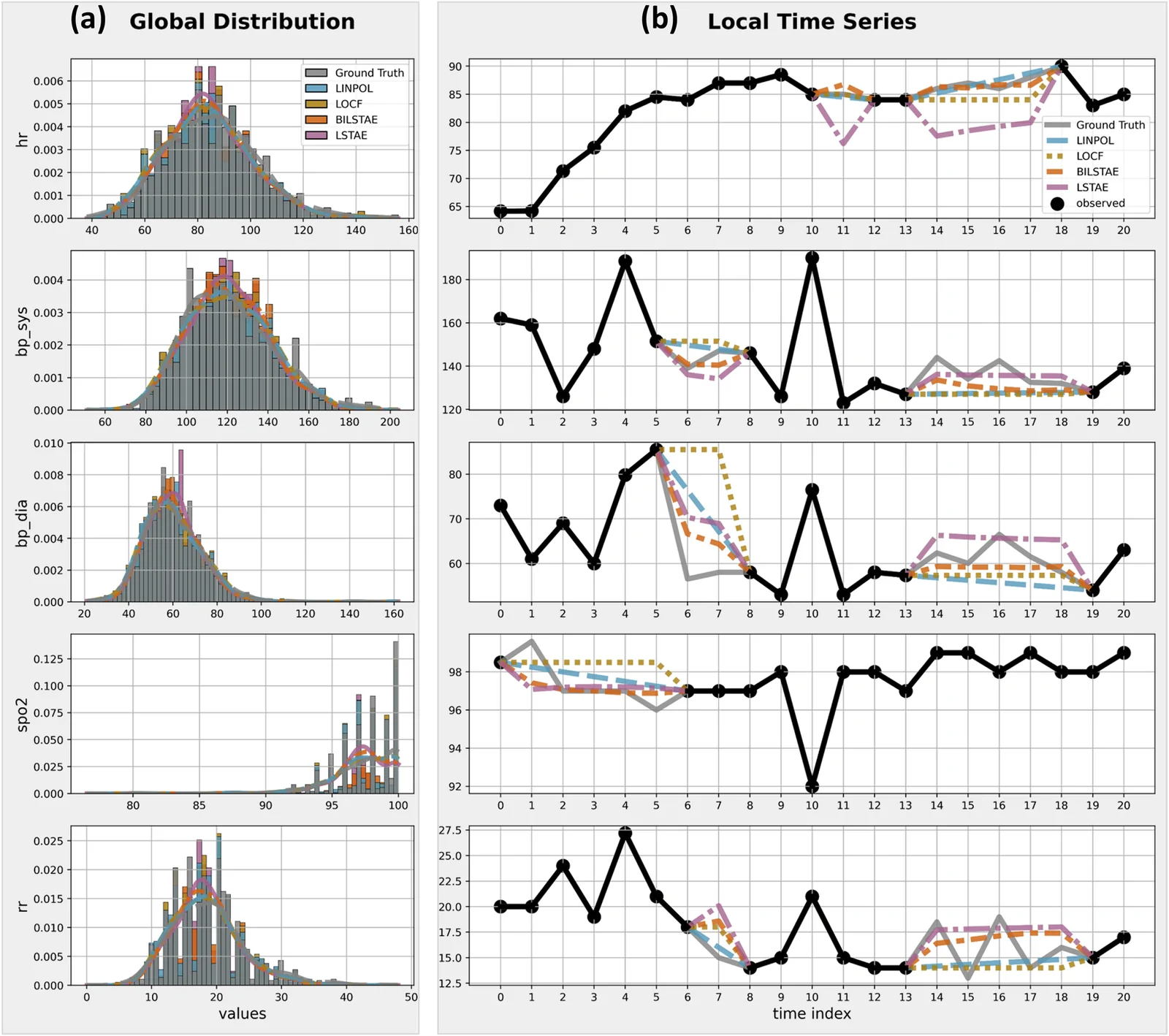
Global distributions and one selected local time series comparing four imputation methods per feature. We display imputation results from linear interpolation (LINPOL), last observation carried forward (LOCF), bidirectional (BILSTM), and unidirectional autoencoder with long-short term memory (LSTM) modules (LSTAE) compared to the ground truth (GT) data for the Medical Information Mart in Intensive Care (MIMIC). In panel **a**, the global distribution of feature values across all patient time series is shown. Panel **b** shows an example of one local time series for one patient, where observed values that did not require imputation are marked as black dots. Source data are provided as a Source Data file.

We visualize results from LINPOL and LOCF as strong baseline models, alongside LSTAE and BILSTAE architectures, as these performed well across all datasets (see Fig. 4). The distribution of imputed heart rate values was shifted to the left (median: 83) for all four methods in comparison to GT data (median: 91) in MIMIC. For bp_sys, BILSTAE introduced two peaks around 112 and 133 mmHg in contrast to the GT distribution expressing one single peak at 119 mmHg (see Fig. 4a). As one example patient time series, BILSTAE and LSTAE improved temporal signals of bp_sys between 5 and 8 h as well as between 13 and 19 h for bp_dia and rr outperforming baselines (see Fig. 4b). BILSTAE alone could capture the temporal spike between 5 and 8 h for bp_dia closest to GT data. For other occasions (e.g., between 6 and 8 h for rr), simple baselines like LINPOL yielded better estimates than autoencoders, which over- or underestimated temporal trends. Though not exclusively, we found differences in distributions and value errors between baselines and autoencoders, explaining divergences in previously reported metrics.

After observing decreased imputation performances of autoencoders under our simulated missingness patterns, we conducted additional analyses on missingness restricted to the MCAR assumption without reflecting spatio-temporal structure. These data did not preserve the spatio-temporal dependencies explicitly simulated by our proposed Markov model. Following prior work, we generated random subsets of CCD by inducing missingness based on original FM rates (see Table A.3 in Supplement A). In contrast to all previous results, autoencoder architectures clearly outperformed simpler models across datasets and performance metrics on this kind of simulated missingness (e.g., NMAE_loc_ = 1.153 ± 0.033, 3.179 ± 0.077, 1.951 ± 0.030 for BILSTAE and NMAE_loc_ = 2.133 ± 0.048, 4.506 ± 0.118, 5.802 ± 0.064 for LINPOL in MIMIC, HIRID, ICDEP). To test the generalizability of these findings, we conducted experiments with well-known DL reference models SAITS, BRITS, CTA, and STGAE (see Table A.9 in Supplement A). Out of all reference models, BRITS yielded the best imputation performances, imputing missingness structure by learning from downstream information (e.g., NRMSE_loc_ = 3.926 ± 0.041). Results indicate that our Markov model created a more demanding imputation benchmark for DL imputation methods than previous work (see Additional Results A.3 in Supplement A). Additionally, we provide a sensitivity analysis of using a variety of scaling factors that amplify MAR and MNAR effects (see Additional Results A.4 in Supplement A).

### Downstream performance

We investigated the influence of imputation performances on downstream classification tasks across amplification scenarios. For our transformer-based downstream prediction model (TRAN), which requires fixed-length sequences, we used the first 12 h after ICU admission for MIMIC and HIRID patients and the first 1.5 h for the perioperative ICDEP cohort. This led to the inclusion of 9233, 8696, and 16,020 patient time series, respectively. Prevalence rates were at 8.71% and 9.73% for in-hospital death in MIMIC and HIRID. In the selected ICDEP cohort, 9.23% patients suffered from POD.

**Fig. 5:**
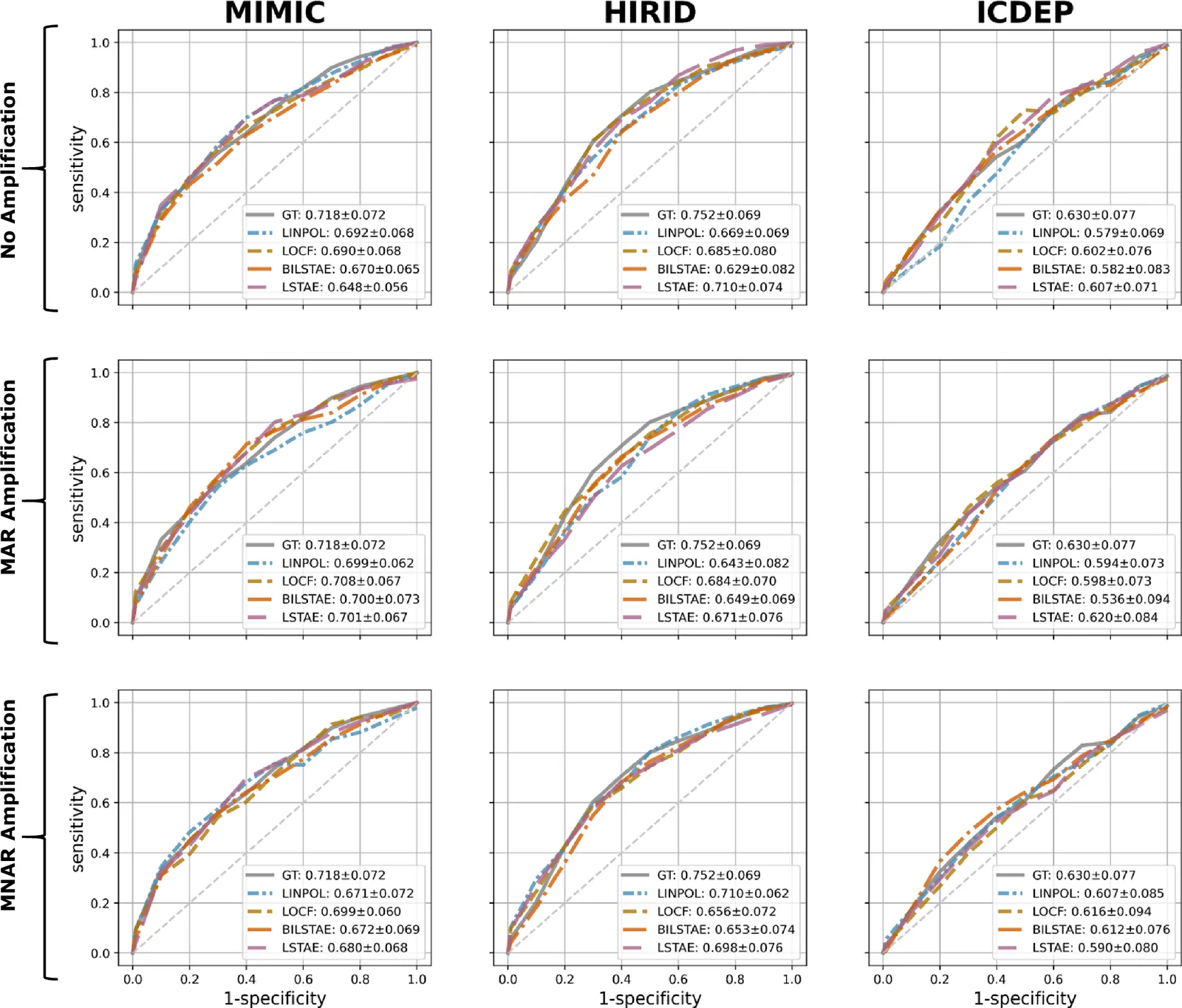
Area under the receiver operating characteristics (AUROC) curves across amplification scenarios. We included three datasets: Medical Information Mart for Intensive Care (MIMIC), High-Resolution ICU Dataset (HIRID), and Intraoperative Care DElirium Prediction (ICDEP) data. We display the area under the receiver operating characteristic curve (AUROC) for ground truth (GT) and imputed datasets. Linear interpolation (LINPOL), last observation carried forward (LOCF), LSTM autoencoder (LSTAE), and bidirectional LSTM autoencoder (BILSTM) were applied for imputations. All results are gained from a downstream transformer model classifying in-hospital death (MIMIC, HIRID) or postoperative delirium (ICDEP). AUROC charts are displayed without any amplification, with missing at random (MAR) amplification, and with missing not at random (MNAR) amplification.

We trained our downstream model on GT data in addition to imputed spatio-temporal missingness patterns by LINPOL, LOCF, and more complex autoencoders, BILSTAE, and LSTAE. We evaluated downstream classification performances by drawing receiver-operating characteristic curves (AUROC) and precision-recall curves (AUPRC) (see Figs. 5 and A.4 in Supplement A). Performances were best for TRAN trained on GT data across all datasets and amplification scenarios, showing elevated AUROC as well as AUPRC metrics. Overall, the prediction of in-hospital mortality (e.g., AUROC = 0.752 ± 0.069 for GT in HIRID) was easier than the postoperative delirium task (AUROC = 0.630 ± 0.077 in ICDEP). We observed minor differences in receiver-operating curves across imputation models and amplification scenarios, with no single method showing a clearly superior performance. Differences were more pronounced for precision-recall curves (e.g., AUPRC = 0.274 ± 0.027, 0.140 ± 0.027 for LINPOL, BILSTAE in MIMIC with no amplification). In MIMIC, downstream models that used LINPOL imputed data expressed higher AUPRC scores than models trained on BILSTAE data. In HIRID, LOCF imputations resulted in better downstream AUPRC metrics than LSTAE (e.g., AUPRC = 0.225 ± 0.096, 0.213 ± 0.096 for LOCF, LSTAE in HIRID with no amplification). Detected trends were widely dispersed across datasets. Most imputation approaches yielded comparable results in downstream classification tasks, suggesting no clear advantage for one method over the others.

## Discussion

Here, we present results from a simulation model that induces realistic spatio-temporal missingness patterns in three real-world clinical longitudinal datasets by sampling from Markov chains, with transition probabilities estimated from real data. Enhancements of simulations led to the amplification of detected MAR effects between missingness and age. MNAR effects were induced using a feature’s missingness with its value levels. The application of 20 imputation methods and their variations showed that commonly used approaches like linear imputations, applicable for study data, or LOCF, eligible for clinical prediction settings, yield good performances on spatio-temporal missingness patterns. MICE imputations estimated global distributions of vital signs within our study population closest to GT data. MICET, which was conditioned on previous observations, led to improved local and distributional errors compared with DL methods, particularly on MIMIC and ICDEP data. When benchmarking imputation performances on simpler MCAR patterns without spatio-temporal aspects, the superior performance of linear methods diminishes in favor of DL models. Finally, we trained a downstream classifier on a variety of datasets where spatio-temporal missingness was imputed by different methods. Classification metrics varied across these datasets, while imputation methods expressed similar downstream performances. High performance of classifying clinical outcomes highlights the potential in temporal vital sign analysis. We applied our Markov chain model to clinical multivariate time series. Additional domains can benefit from our simulation approach when spatio-temporal missingness patterns are a burden (e.g., traffic or geographic data).

Akin to Jäger et al.^38^, we developed an open, accessible benchmarking suite, evaluating temporal imputation methods on clinical time series instead of tabular data, similar to Bertsimas et al.^14^. For baseline comparisons, we replicated previous studies that limited missing simulations on MCAR scenarios with varying missingness rates^17,21,25,29,30,32^. In contrast, our proposed Markov chain model further reflects original spatio-temporal patterns and is capable of simulating MAR or MNAR mechanisms that condition missingness on demographics or the underlying value levels^20,23,42^. Li et al.^43^ and Tan et al.^44^ detected MAR patterns of laboratory values related to illness severity or hospital sites. In our data, age was positively correlated with the fraction of overall missing values per feature in ICDEP. We comprehensively discuss the clinical utility and changes in observed spatio-temporal patterns for MAR and MNAR effects in Additional Discussion A.1 and Additional Discussion A.2 in Supplement A. Here, we extensively describe original missingness patterns, such as the simultaneous absence or presence of systolic and diastolic BP values, and show key differences between real-world clinical datasets. While some of these patterns have been analyzed in MIMIC^17,28,40,45^, we are not aware of any work that has built upon HIRID or an intraoperative dataset like ICDEP. Marginal differences in descriptive statistics between original and simulated spatio-temporal patterns underscore the quality of our simulation model.

Lai et al.^30^ and Qian et al.^26^ presented DL autoencoders that improved temporal imputations of clinical time series over standard statistics. Hence, we hypothesized that trained spatio-temporal autoencoders can outperform simpler methods like linear imputation. Inspired by previous studies^24,26,30,45^, we designed a spatio-temporal autoencoder architecture using (Bi)LSTM modules. Surprisingly, simple approaches such as linear imputation, LOCF, and MICE, widely used by medical studies^11,17,21^, outperformed more complex autoencoders in most scenarios. As previous publications suggested^42,46^, MICE reconstructed value distributions across all patient time series closest to the known distributions. Selected studies claim superior imputation performance of proposed DL methods^20,21,26^. In contrast, we found more generalizable studies that covered a larger variety of datasets, highlighting the benefit of simpler linear imputation models over DL^21,38,42^.

Related work by Jäger et al.^38^ showed that commonly used imputation methods, such as KNN or MICE, outperform DL autoencoder on tabular data for complex MNAR scenarios. Benchmarks by Kazijevs et al.^47^ found slightly improved performance evaluations for spatio-temporal DL imputers on longitudinal health records but questioned the benefit of these methods over simple linear models due to high computational expenses. Jahangiri et al.^48^ imputed systolic and diastolic BP values, recommending mean aggregates instead of more complex linear mixed effects models. None of the related studies simulated spatio-temporal missingness patterns for validating imputation model performances. Similar to Kazijevs et al., baseline analysis with simple MCAR induction, omitting spatio-temporal structure, showed superiority of DL autoencoders over linear models in these more unrealistic scenarios.

We hypothesize that STAEs more easily infer feature values from spatial dependencies when unobserved patterns lack spatio-temporal structure. As an example, systolic BP can be inferred from diastolic BP and vice versa when one of the two features is present, due to a high correlation between these two variables. Specifically, we found Pearson correlation coefficients of 0.55, 0.68, and 0.78 in MIMIC, HIRID, and ICDEP between these two features, similar to Jahangiri et al.^48^, supporting this hypothesis. When analyzing the local NRMSE bp_sys for the oversimplified MCAR scenario, errors were much lower with 0.630 ± 0.007, 1.819 ± 0.017, and 5.510 ± 0.058 compared to the realistic spatio-temporal scenario that resulted in 9.897 ± 0.112, 10.198 ± 0.098, and 12.564 ± 0.108 for MIMIC, HIRID, and ICDEP. Likewise, all trained reference models, such as BRITS, SAITS, or CTA, which learn spatio-temporal structure in clinical time series, yielded more convincing imputation results on simple MCAR inductions. We argue that our simulation model introduces more realistic missingness patterns in clinical time series, such as the joint absence of BP values, leading to complex scenarios that result in diminished performance of STAE autoencoder models. Additional benchmarks, which were restricted to MCAR mechanisms without spatio-temporal structure, suggest that previous studies^24,26,30^ may have overestimated the performance of DL models.

Our findings suggest the usage of linear interpolation for retrospective clinical studies since clinical values were reconstructed sufficiently across datasets and missingness scenarios. If smaller errors from one patient time series can account for larger errors from other patients, the popular method MICE reconstructs global distributions of clinical vital signs^49^. LOCF demonstrated robust imputation performance and remains widely used in clinical ML studies, despite ongoing criticism for its potential to introduce bias and underestimate variability^18,50^. Our findings suggest that using LOCF remains a reasonable approach for imputations prior to prediction tasks. When designing more complex autoencoder architectures, we recommend incorporating static attributes, such as age, as they improve model performance, particularly under more complex MAR scenarios. While sequence lengths were overall reduced for CCD compared to the original data, different strategies for creating complete sets of consecutive available features might have resulted in increased learnable signal for STAE models. However, we showed similar findings across all three datasets that comprised heterogeneously sampled clinical time series, increasing the generalizability of the presented findings.

Related studies demonstrate that identifiability and consistency are guaranteed for simple averaging techniques under restrictive MCAR assumptions^51,52^. These properties remain theoretically elusive for DL architectures like autoencoders. We posit that our Markov missingness model provides a robust method for simulating realistic missingness patterns beyond MCAR assumptions. The model yields consistent estimates of transition probabilities that converge to the true missingness process as the sample size increases. Further research is required to explore the behavior of autoencoders, imputing such realistic missingness patterns in clinical time series.

When assessing MAE and RMSE grouped by feature without normalization, systolic blood pressure (bp_sys) consistently yields the highest errors (max. of 13.58 ± 0.42 MAE and 18.27 ± 0.42 RMSE) (see Table A.10 in Supplement A). Elevated errors for bp_sys occur likely due to high variability and wider physiological range of blood pressure readings compared to more stable vitals (SD of max. 29.99 mmHg in CCD). Conversely, blood oxygen (spo2) exhibits the lowest errors (min. of 0.66 ± 0.01 MAE and 1.34 ± 0.04 RMSE), reflecting its narrower clinical range where even small deviations can be physiologically relevant. When comparing metrics, MAE values are notably higher than RMSE across all features, expressing a large deviation for respiratory rate (rr) recordings in HIRID (9.27 ± 1.01 MAE, 28.45 ± 3.67 RMSE for LINPOL). These deviations confirm increased sensitivity to large outliers for high-frequency vital signs, which are often caused by critical acute changes or sensor noise. We discuss further clinical justification for seldomly occurring missingness patterns in Additional Discussion A.3 in Supplement A.

**Fig. 6:**
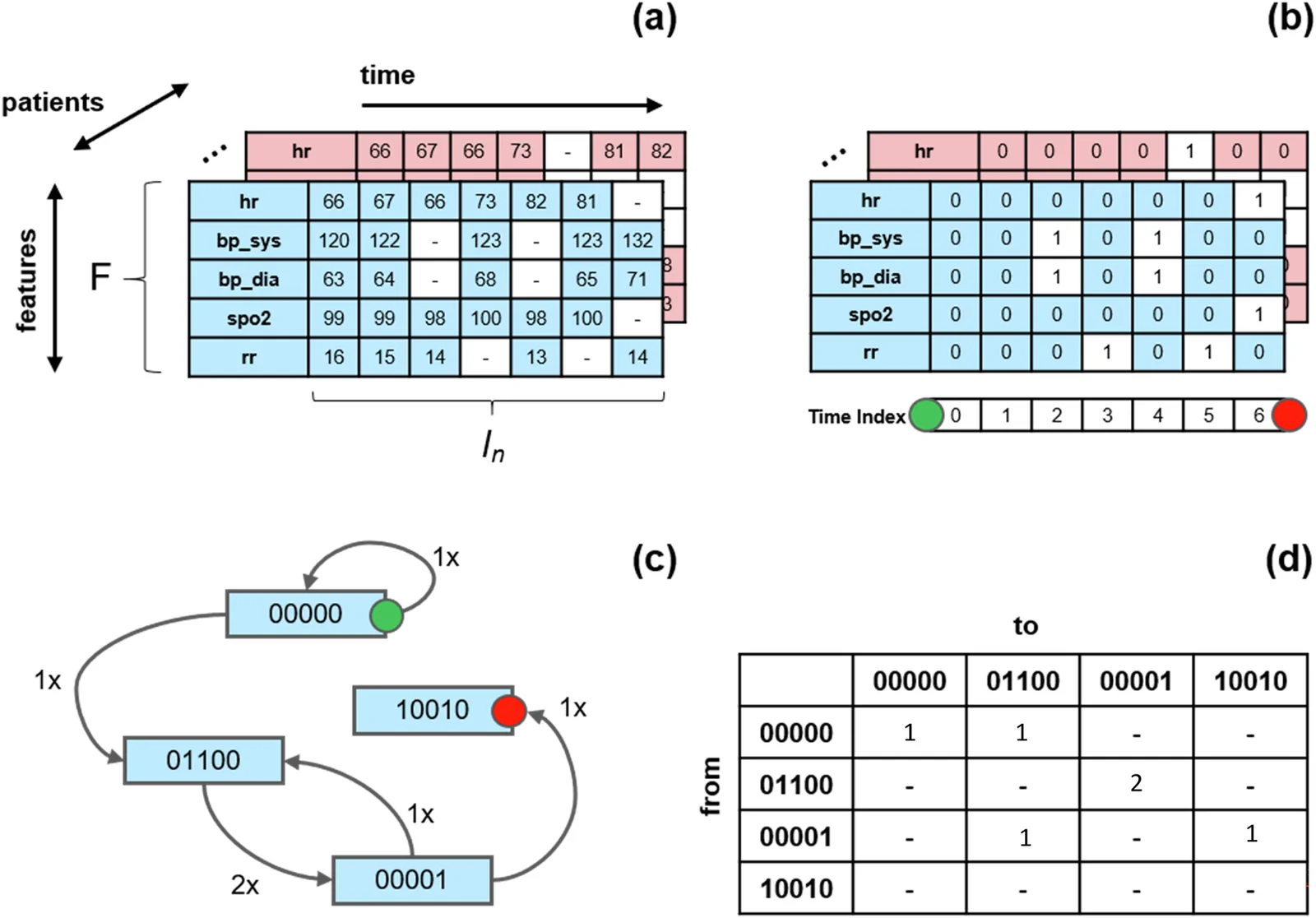
Illustration of spatio-temporal missingness and the Markov chain model. We show an illustrative example with *F* = 5 features: heart rate (hr), systolic (bp_sys) - and diastolic (bp_dia) blood pressure, blood oxygen saturation (spo2), and respiratory rate (rr). A data matrix ${{{{\bf{X}}}}}_{n}\in {{\mathbb{R}}}^{F\times {l}_{n}}$ is stored for each patient *n* holding values for *F* features at time indices *i* ∈ {0, …, *l*_*n*_}. Panel **a** shows two examples for **X**_*n*_ (colorized), dimensions across features, patients, and time are highlighted. Panel **b** shows corresponding binary missing indicator matrices ${{{{\bf{R}}}}}_{n}\in {\{0,1\}}^{F\times {l}_{n}}$ for each **X**_*n*_. In panel **c**, we illustrated all four distinct temporal slices (building the Markov states) that occurred in the blue **R**_*n*_ (of dimensions 5 × 7) as missing vectors in boxes. The occurrences of transitions from one temporal slice to another are counted (shown as numbers next to arrows), resulting in a Markov chain. In panel **d**, the absolute counts of transitions are inserted into a transition table for the observed state space in (**c**). In contrast to this minimal example, we retrieved all absolute counts of transitions covered by the entire population and normalized them across all patient time series per dataset so that rows sum up to one.

Missingness in clinical time series can follow complex periodic patterns. The proposed Markov chain simulation method is intended to approximate the observed missingness structure, rather than to explain the underlying data-generating process of clinical variables or unobserved patterns. A higher-order Markov process would increase the model’s flexibility to learn further oscillations and distinct missingness patterns. Evaluating the order of such a process is beyond the scope of this study. However, Fig. 6 clearly demonstrates that original auto- and cross-correlation patterns were reproduced in the simulated data.

A variety of related studies have evaluated downstream classification tasks, such as ours, predicting in-hospital death^14,23,26^, heart failure^23,25^, or kidney disease^20^. Psychogyios et al. found that DL imputed data improved the prediction of coronary heart disease^23^ while KNN or MICE performed better for small missingness rates. Zamanzadeh et al. showed that the accuracy of kidney disease classification varies a lot across prior imputed sets, independent of their imputation^20^. Luo et al. trained autoencoders, which improved imputation as well as in-hospital death classification^17^. Qian et al. argued that results from downstream classifiers are decoupled from prior imputation performances^26^. In our study, we also found decoupled behavior, resulting in improved downstream performance while imputing time series with more deficits. In this study, we conclude that the downstream performances of simpler methods like LOCF are sufficient.

This study has several limitations. While in-hospital death is less clinically valuable compared to actionable outcomes (e.g., hypotension), this endpoint in MIMIC and HIRID is technically seen as an adequate downstream task. Related literature suggests that patient death can serve as an effective auxiliary target variable for evaluating clinical prediction or imputation models^53,54^. While we acknowledge that utilizing diverse labels would enhance the impact of downstream analysis, the limited availability of clearly defined endpoints in real-world databases like MIMIC and HIRID remains a significant impediment. For the ICDEP dataset, POD is reflecting a clinically actionable and valuable endpoint^16^. When considering the best downstream model, LSTAE imputations resulted in an AUROC of 0.710 ± 0.074 and an AUPRC of 0.213 ± 0.096 in HIRID on simulated data without any further MAR or MNAR amplifications. For a fixed threshold where the Youden index was maximized^55^, this model achieved a maximum sensitivity of 0.65 and a specificity of 0.60 with a corresponding 0.18 precision. Assuming 100 cases, of which 10 are associated with a lethal outcome. Here, 7 out of 10 patients at risk (true positives) would be correctly identified. 54 patients that are not at risk (false positives) would be correctly labeled, whereas 36 cases would be falsely predicted at risk (false positives). This simple numerical example shows that setting clinical thresholds is a non-trivial task. Low prevalence rate and low precision result in overtreatment, expressed by a high number of false positives. As another limitation, multi-purpose DL architectures like BRITS have been designed to learn both downstream and imputation tasks simultaneously, contrasting our approach^6,34,40,56^. In additional experiments, we showed that this learning strategy can be beneficial for imputing clinical time series as well as solving a downstream task. However, we would rather treat these two aspects as independent problems (e.g., no restriction to one ML setting). A majority of proposed architectures are not capable of processing sequences of unequal lengths. We see an extension of models like BRITS^34^ or attention networks^30^ to meet this requirement as potential future work. Our configured LSTAE and BILSTAE models could be successfully trained with a batch size of one and accumulate gradients for addressing this issue (see Methods). The presented simulation model is limited to the selection of five highly available vital signs due to strong availability constraints across features and along the temporal dimension. Prior work evaluated imputation methods on laboratory values^43^, which we did not consider. We described selection biases due to preprocessing for the original, CCD, and imputed sets. Constraints for creating analyses with CCD introduced some shifts in demographics or correlations between features. There is no back-tracing of simulated missingness to individual patients, potentially affecting clinical data analysis. The inclusion of three different datasets increased the generalizability of our results, but other datasets might result in the detection of different spatio-temporal patterns. We foster replicability and transparency of our results by making models and processed data publicly available^57^.

We present a Markov Chain simulation model to successfully induce spatio-temporal missingness patterns in clinical time series. We evaluate commonly used linear imputation methods as an overall robust way to impute unobserved values in time series for sufficient downstream performance. Previous studies, which claimed superior performance of DL methods, lack in spatio-temporal aspects, which were simulated by our proposed model. We provide an open source benchmarking suite, enabling future ML projects to increase their robustness and transparency.

## Methods

### Ethics statement

For processing ICDEP data, the Institutional Review Board (IRB) approval EA4/254/21 was granted by the ethics committee at Charité-Universitätsmedizin Berlin (chair: Prof. Dr. med. R. Morgenstern). For MIMIC, the IRB at the Beth Israel Deaconess Medical Center granted a waiver of informed consent and approved the data-sharing initiative. The IRB of the Canton of Bern approved the collection of patient data for the HIRID database. Further legal information can be found at the PhysioNet (https://physionet.org) platform.

### Data retrieval

This study uses three different clinical datasets consisting of pre-processed EHRs. Two public datasets (MIMIC and HIRID) contain medical records collected during ICU stays, while one in-hospital dataset (ICDEP) contains intraoperative variables recorded during surgeries.

We applied our methods to the third version of MIMIC^58^. This public database from the U.S. contains data of 38,597 patients admitted to ICUs between 2001 and 2012 at the Beth Israel Deaconess Medical Center in Boston. We used data that were pre-processed with a data pipeline provided by Wang et al.^59^. The pipeline semantically integrates features that describe the same physiological parameters (e.g., invasive and non-invasive BP) while resampling time series to an hourly equidistance grid. Wang et al. selected frequently used features related to vital signs, clinical interventions, and laboratory results for clinical prediction tasks. They limited their cohort to adult patients (≥18 years) with a minimum of 12 h of recorded time series. Although multiple ICU admissions for the same patient could be observed, the authors included one distinct time series per patient. The openly accessible pre-processed data on PhysioNet cover 34,472 patients with 104 time-dynamic features^58^.

The high-time-resolution ICU dataset HIRID contains data of 36,098 adult patients admitted to the Bern University Hospital in Switzerland between 2008 and 2016^41,60^. The original dataset stores 681 variables, including bedside monitoring data, settings of medical devices, laboratory values, and administered drugs. In the original publication, Hyland et al.^60^ selected and semantically integrated 209 clinical variables for the prediction of circulatory failure, focusing on cardiac patients. We acquired data from Physionet and manually selected 101 of these 209 features, omitting non-numeric and static ones while integrating identical physiological features similar to Wang et al.^59^ (see Table B.1 in Supplement B). We mean-aggregated and resampled the data into a 10-min equidistant grid as suggested by the original publication^60^. HIRID treats each ICU admission individually and assigns a unique patient identifier to each hospital stay, increasing patient privacy.

Furthermore, this study used data from the internal Intraoperative Care DElirium Prediction dataset (ICDEP) acquired at the German medical center Charité-University Medicine Berlin. During surgical interventions, patients receive high-frequency monitoring for the duration of the surgical procedure^61^. ICDEP was originally created on the basis of monitoring data for the prediction of postoperative delirium^16,62,63^. The dataset comprises 41,832 adult patients admitted between 2017 and 2020. We identified the most predictive features that are available for at least 10% of all patients. We semantically integrated features that described the same physiological parameter. As a result, 61 intraoperative time series features were selected and mean-aggregated into a 5-min equidistant time grid. Due to some patients undergoing multiple surgeries, we reduced our time-series data to the longest sequence per patient to minimize the loss of temporal dynamics.

All three datasets contain demographic information like sex and age. After resampling of time series to increase their availability, we explored the joint missingness of different feature sets. We then selected five vital signs that are highly available across all three datasets (see Results section).

### Spatio-temporal missingness

The data pre-processing pipelines yielded one data matrix ${{{{\bf{X}}}}}_{n}={({x}_{j,t})}_{n}$ with ${{{{\bf{X}}}}}_{n}\in {{\mathbb{R}}}^{F\times {l}_{n}}$ for each *n*-th patient, storing *f* ∈ {1, 2, …, *F*} features at time indexes *t* ∈ {1, 2, …, *l*_*n*_} where the time sequence length *l*_*n*_ varies across patients (see colorized tables in Fig. 6a). Thus, pre-processed data exhibited spatial relationships across features and temporal relationships across time for a population of *N* patients. For each **X**_*n*_, we derived a binary missing indicator matrix ${{{{\bf{R}}}}}_{n}={({r}_{j,t})}_{n}$ where

$${r}_{j,t}=\left\{\begin{array}{ll}1\quad &\,{{\rm{if}}}\,{x}_{j,t}\,{{\rm{is}}}\,\,\,{{\rm{missing}}}\,\\ 0\quad &\,{{\rm{if}}}\,{x}_{j,t}\,{{\rm{is}}}\,\,\,{{\rm{observed}}}\,\end{array}\right. ({{{\rm{see}}}}\; {{{\rm{colorized}}}}\; {{{\rm{tables}}}}\; {{{\rm{in}}}}\; {{{\rm{Fig}}}}.\;6{{{\rm{b}}}})$$

The quantity ${Q}_{n,j}={\sum }_{t=1}^{{l}_{n}}{r}_{j,t}$ calculates the number of missing values per patient *n* for the *j*th feature. We define feature missingness (FM) as the fraction of missing values for feature *j* across all time steps and patients:

1 $${{{{\rm{FM}}}}}_{j}=\frac{{\sum }_{n=1}^{N}{Q}_{n,j}}{\mathop{\sum }_{n=1}^{N}{l}_{n}}.$$

The defined missingness metric FM_*j*_ was used to explore and select features that were available across all datasets and to quantify differences between original and simulated spatio-temporal missingness patterns. Values of FM_*j*_ range between 0 and 1, where levels of 0.2 are considered low due to the inclusion of multiple sequences with varying lengths in Eq. (1). We also report additional missingness metrics, like block missingness, in Supplement A to further prove the quality of our simulated missingness patterns (see Additional Methods A.1).

### Complete case data

For generating CCD without missingness, the resampled original data were initially cleaned with two separate cleansing strategies. We observed some outliers across all datasets holding physiologically impossible values, e.g., spo2 exceeding 100%^2^. Similar to previous studies^16,59,64^, we used data ranges of [5, 400] bpm for hr, [2, 300] bpm for rr, [10, 100]% for spo2, [20, 400] mmHg for bp_sys, and [10, 300] mmHg for bp_dia to detect outliers. Feature values that fall outside these defined ranges are marked as outliers. The first cleaning strategy preserved the original spatio-temporal missingness by setting outliers to the nearest edges of the valid ranges, e.g, 120% Spo2 was set to 100%. Thereby, outliers were corrected instead of being removed from the dataset. We report results from this strategy in the main manuscript. In contrast, the second strategy sets outliers to missing, also altering spatio-temporal missingness patterns that were learned by the proposed simulation model. At this point, no patients were excluded from the cleaned dataset under either of the data cleaning strategies.

For the generation of CCD, we selected the largest continuous block of available data in each data matrix. We therefore reduced every binary missing indicator matrix **R**_*n*_ to the longest sequence of consecutive zeros and kept the corresponding values of **X**_*n*_. As another constraint, resulting sequences should exhibit a minimum length of three values. All other data that did not comply with these conditions were discarded from the CCD. The selection of early available values in multivariate time series allowed the temporal alignment of patient trajectories to the beginning of ICU stays (MIMIC, HIRID) or surgical (ICDEP) intervention across the entire patient cohort. Slicing of different sections of available feature values alongside the temporal dimension did not result in extended sequence lengths. Lastly, the assignment of one unique CCD time series per patient increased the reliability of estimating confidence intervals across independent samples, as well as reducing estimation bias when performing train-test splits. The generation of CCD also resulted in the overall reduction of sequence lengths (see Results section).

### Simulation model

We define a state space *S* where each state *s*^*k*^ in *S* = {*s*^1^, *s*^2^, …, *s*^*K*^} represents a distinct temporal slice ${({{{{\bf{r}}}}}_{:,t})}_{n}$ of the binary missing indicator matrix **R**_*n*_ (see Fig. 6c). We stored the occurrences of unique states in a frequency table FT = {*f*^1^, *f*^2^, …, *f*^*K*^} ∈ [0, 1]^*K*^ where each *f*^*k*^ denotes the marginal probability of the occurrence of each *s*^*k*^ across all patients. Assuming the Markov property^65^, stating that the probability for moving to the next state depends only on the current state, we estimated transition probabilities between states in *S* yielding a transition matrix **P** ∈ [0, 1]^*K*×*K*^, with rows summing up to one (see Fig. 6d).

For the induction of learned spatio-temporal missingness patterns on complete time series, we simulated one binary missing indicator matrix ${\hat{{{{\bf{R}}}}}}_{n}\in {\{0,1\}}^{F\times {\hat{l}}_{n}}$, with ${\hat{l}}_{n}$ as the (potentially) reduced sequence length, for each patient *n* that remained in the CCD, using the following strategy:

- We sampled the initial state *s*_0_ that yields the first temporal slice of ${\hat{{{{\bf{R}}}}}}_{n}$ as ${({\hat{{{{\bf{r}}}}}}_{:,0})}_{n}$ by drawing one state *s*^*k*^ out of *S* with the probability *f*^*k*^ from FT.
- For generating the next state *s*_*i*+1_, we draw a sample using the transition probabilities defined by **P**.
- Step 2 was repeated until all ${\hat{l}}_{n}$ temporal slices are filled resulting in the simulated ${\hat{{{{\bf{R}}}}}}_{n}$.

Due to the state space being finite, we could estimate occurrence as well as transition probabilities for all included states *s*^*k*^, building the basis of our discrete Markov chain model^66^. We describe the change of original and simulated spatio-temporal patterns in the Results section. Due to the artificial induction of missingness on CCD, we have access to the GT values, which allows us to quantitatively assess the quality of imputation methods.

### Amplification of missingness patterns

Extracted clinical time series can potentially exhibit all three missingness types MCAR, MAR, and MNAR^7^. If values are MCAR, the available data is independent from co-variates or other (un)observed data. While we assume that MCAR occurs inherently in our detected spatio-temporal missingness patterns in the original datasets (e.g., random malfunction of Spo2 sensor), we did not further artificially increase these effects unlike previous authors^17,21,27,32^.

We estimated existing age-related MAR effects as *p*(**R**∣age) in the original data that were artificially amplified. To this end, we introduced three equally sized age bins across all datasets (age bins = {[18,51], (51,69], (69,120]} years). We then modified the probability for the state **s** = [1, 1, 1, 1, 1] where all features are missing, denoted as the total absent state (TAS). For a positive MAR effect, the occurrence as well as transition probabilities for the TAS were increased for elderly patients by multiplying probabilities with a factor that corresponds to the previously defined age bins (age factors = {1.2, 2.2, 3.2}, higher factors were applied to bins of advanced age). By increasing the probability of TAS for elderly patients, all subsequent transition states that were sampled from the Markov chain depend on age, reflecting a controlled simulation scenario for induced MAR effects. For a negative MAR effect, probabilities were increased for younger patients by applying factors in a reverse order (age factors^−1^ = {3.2, 2.2, 1.2}). We decreased the probabilities of the total present state (TPS), encoded as **s** = [0, 0, 0, 0, 0] where all features are observed, by the margin that was added to the TAS so that probabilities sum up to one.

Since we know the GT underlying values for the induced spatio-temporal missingness on CCD, we further conditioned missingness on GT value levels after all steps in the Simulation Model to amplify MNAR. We repeated the previously described binning procedure for GT feature values resulting into a *F* × 3 matrix holding one of three bins per feature (see Table A.4 in Supplement A). For a positive MNAR effect, we induced more FM for values falling into higher value bins by multiplying probabilities with increased factors (value factors = {1.8, 2.8, 3.8}). Unlike MAR, MNAR effects cannot be assessed in our original datasets since the reasons for missing values were not documented.

### Imputation methods

#### Simple imputation models

We applied 20 imputation methods (see Table 3) via a 3-fold cross-validation (CV) approach, where statistics are derived from 66% (training data) and applied to 33% (testing data)^67^. The CV splitting of time series and fittings of imputation methods were repeated three times on distinct chunks of simulated patient data. We estimated median and mean statistics per feature *j* on training data across all patients and time (PMEAN, PMEDIAN as population means/median) as well as at the initial time step (*t* = 0) across patients (1MEAN, 1MEDIAN as 1st time step mean / median). Additionally, we retrieved both statistics as running means or medians (RMEAN, RMEDI) within one single patient time series on previously recorded observed values (from time 0, 1, …, *t*) to impute the next missing value (at time *t* + 1) per feature. Hence, the time-causal order of datapoints was preserved.

**Table 3:** Overview of applied imputation methods

ID	Name	Imputation method	Cross population	Cross features	Time causal	Static features
*Averaging methods*						
1	PMEAN	Population mean	True	False	False	False
2	PMEDI	Population median	True	False	False	False
3	1MEAN	Mean initial time steps	True	False	True	False
4	1MEDI	Median initial time steps	True	False	True	False
5	RMEAN	Running mean	False	False	True	False
6	RMEDI	Running median	False	False	True	False
*Interpolation methods*						
7	LINPOL	Linear interpolation	False	False	False	False
8	NEAPOL	Nearest interpolation	False	False	False	False
9	QUAPOL	Quadratic interpolation	False	False	False	False
*Extrapolation methods*						
10	LOCF	Last observation carried forward	False	False	True	False
11	LINEXT	Linear temporal extrapolation	False	False	True	False
12	QUAEXT	Quadratic temporal extrapolation	False	False	True	False
*Multivariate imputations by chained equations*						
13	MICTRE	MICE random forest	True	True	False	True
15	MICKNN	MICE *K*-nearest neighbor	True	True	False	True
14	MICET	MICE Temporal	True	True	True	True
*Denoising autoencoder*						
16	SAE	Stacked DAE	True	True	False	True
17	MIDA	Multiple imputations DAE	True	True	False	True
18	BILSTAE	Spatio-temporal BiLSTM DAE	True	True	False	True
19	LSTAE/GRUAE	Spatio-temporal GRU/LSTM DAE	True	True	True	True
20	STGAE	Spatio-temporal graph DAE	True	True	True	True

We imputed data with 20 different methods, configuring several model variants. We fitted averaging methods like running means, inter- and extrapolation methods, and a denoising autoencoder (DAE). Multivariate chained equations (MICE) and long short-term memory (LSTM) models are included. Methods could infer statistics across patients (cross population), cross features, and time-causal, as preserving the timely order, not taking values from future time points. Models optionally used static feature attributes like age.

Inter- and extrapolation methods^68^ were applied for one single patient time series per feature. LINPOL and nearest neighbor (NEAPOL) imputation required two observed values (at *t* and *t* + *m* with $0 < m < {\hat{l}}_{n}$) to impute missing values in between. To fit a quadratic interpolation (QUAPOL) term, at least three observed values were required within one time series. If fewer values were observed, interpolation fell back to a lower-order linear term. As the most prominent method from related work, LOCF was applied^18,69^, which simply uses the last observed value (at *t*) to impute the next missing one (at *t* + 1). Similar to RMEAN and RMEDI, linear- and quadratic extrapolation methods relied only on previously observed values. If initial values (at *t* = 0) within one time series were unobserved, 1MEDIAN was applied to replace them.

The method MICE^11,13^, originally not designed for learning temporal correlations, fills initial missing values for *F*−1 features with placeholders like PMEAN. Then, the method approximates the unobserved values for one feature *j* from all other features by fitting a function over the entire population. This approach is iteratively repeated across features until imputed values converge. We deployed MICE models using random forests (tree estimators) (MICTRE) and KNN (MICKNN) as approximation functions, respectively, while multivariable linear regression did not yield convincing results (see Table 3). We use the MICE algorithm to iteratively impute missing values using chained equations, applying a conditional regression model for each partially observed variable, conditioned on the remaining variables in the dataset. At each iteration, imputations are updated sequentially, but intermediate imputed datasets were not retained for this study. Rather than performing multiple imputation (MI), resulting in a number of differently imputed datasets, we used the single completed dataset obtained after 40 iterations. Hence, all our methods displayed in Table 3 are single imputation approaches. Downstream tasks were also not compared across different MI imputed sets. Therefore, Rubin’s rule for pooling results was not applied in our experimental setup. In addition to the standard MICE method, we also evaluated an enhanced temporal version, denoted as MICET, where the input space is conditioned on previously measured records for each feature. Tree-based estimators were chosen for MICET because they improved upon linear estimators by modeling non-linear relationships. The models MICE and STAE were fitted on additional static features, which were always observed to potentially improve imputation performances.

#### Autoencoder architectures

Table 4 displays the hyperparameter search space for autoencoder architectures. The autoencoders SAE and MIDA follow an encoder–decoder architecture^15,42^ to reconstruct missing data. SAE was configured with four layers of stacked MLPs with configurable dimensions *d*_sae_, where the initial input dimension was always set to the number of selected features *F* = 5. MIDA stacks three MLPs sequentially and uses a fixed factor *Θ* to determine the dimension *d*_mida_ per *q*th layer with *F* + *Θ* × *q* (see Table 4). SAE uses logistic sigmoid and MIDA hyperbolic tangent functions as activation functions^70^.

**Table 4:** Learnable hyperparameters for autoencoder models

Autoencoder model	Hyperparameter
SAE	*d*_sae_ = {32, 16, 8, 4}, {128, 64, 32, 16}, {256, 128, 64, 32}
MIDA	*d*_mida_ = *F* + *Θ* × *q*, *Θ* = {4, 6, 9}
STAE	*d*_stae_ = {32, 64, 128, 256}
STGAE	*d*_stgae_ = {32, 16}, {64, 32}, {128, 64}
All	lr = {1e^−4^, 1e^−5^, 1e^−6^}
	bz = {32, 64, 128}
	dr = {0.5, 0.75, 0.9}
	wd = {0, 1e^−1^, 1e^−2^, 1e^−3^}

The dimensions *d* are learned per specific model as the size of the latent space in an encoder-decoder architecture. For the stacked autoencoder (SAE) and multiple imputation denoising autoencoder (MIDA), *d* determines the dimensions of stacked multi-layer perceptrons (MLPs), where *F* defines the number of input features. For MIDA, the dimension of each MLP on the *q*th layer is calculated with factors of *Θ*. Spatio-temporal autoencoders (STAEs) comprise either one or two modules of (bidirectional) long-short term memory as (Bi)LSTM modules, or gated recurrent units (GRU) with hidden dimensions of *d*_stae_. The spatio-temporal graph autoencoder (STGAE) has different stacks of layers for applying convolutions. The learning rate (lr), dropout rate (dr), and weight decay (wd) are optimized for all autoencoders, independent of their architecture. We trained STAEs with a batch size of one and accumulated gradients after bz steps. For all other autoencoders, the batch size was directly equal to bz.

We chose the number of nodes per layer for the simple stacked autoencoder (SAE) in a monotonically decreasing manner, halving the number of nodes for each subsequent layer (e.g., 32, 16, 8, 4), as it is common for most DL architectures^71^. We provided three different sets of such node stackings to test small, medium, and high capacity models that compress inputs to a bottleneck dimension on the last layer. Hereby, the model focuses on layer-wise compressed inputs, avoiding overfitting^72^. The authors Gondara et al., who originally proposed the MIDA architecture, suggested a hyperparameter *θ* of 7 and varying it around this value^15^. Consequently, we tested different configurations of *θ* (4, 6, 9) and configured a lower dimensionality for shorter multivariate time series from MIMIC and a higher dimension for longer sequences in HIRID.

Inspired by Li et al.^31^, a vanilla STAE model was designed that uses one or two stacked LSTM or GRU modules. For these modules, four different numbers of hidden states (32, 64, 128, 256) were integrated into the search space, as this selection is commonly chosen for published autoencoder architectures^73,74^. Dropout and weighted decay, as implemented by an L_2_-norm, were implemented as regularization mechanisms, actively reducing the chances that models simply learn samples instead of generalizable trends. By applying Hyperband, we drastically reduced the computational complexity and time for training multiple model configurations in parallel. However, we are aware that a fully exhaustive search, such as Grid search, could have led to different parameter selections, which would have required too many training resources for our experiments. Following established literature, we fitted autoencoder models to real-world data, utilizing empirical heuristics to determine optimal hyperparameter configurations.

SAE and MIDA infer spatial relations, rather than temporal ones, globally for the entire population. In contrast, STAEs learn from both relations using DL models like LSTMs to reconstruct time series (see Fig. A.5 in Supplement A). We configured the STAE architecture, motivated by similar studies^22,30,31^, with one or two stacked GRUs (GRUAE), LSTMs (LSTAE), or bidirectional LSTMs (BILSTAE) (see Table 3). The size of the latent space, *d*_stae_ determined the number of hidden neurons (see Table 4). The latent space was optionally enhanced in the temporal dimension by the output of one perceptron, combining a linear layer with logistic sigmoid activation, to process static features such as a patient’s age. Due to the processing of time series with varying lengths, we used a batch size of one and accumulated gradients after ag = bz steps^75^ (see Table 4).

Fan et al.^33^ developed the so-called STGAE architecture with two stacked CNN modules for the encoding and decoding process. We trained the model with dimensions of *d*_stgae_. In addition to CNNs, the proposed architecture comprises a graph for static attributes. We configured this graph with demographics where Euclidean distances (edges) were calculated between patients (nodes) for the attributes age and sex. For applying the CNN to unequal lengths of sequences, we used padding where the padding token did not contribute to the loss.

We *z*-standardized train and test time series by calculating means and standard deviations on the three training folds for all autoencoders. Afterward, missing values were filled with ones or zeros as noise. Training statistics were used for de-standardization after decoding. As a reconstruction loss, we configured an MSE function^76^ as *l*_MSE_ for SAE, MIDA, and STGAE. For our configured STAE models (GRUAE, LSTAE, BILSTAE), we either used an unmodified MSE loss or configured a combined loss (cl) as a linear combination of *l*_MSE_ and the Wasserstein distance (WSD)^77^ loss *l*_WSD_ as cl = *λ*_1_ × *l*_MSE_ + *λ*_2_ × *l*_WSD_. Empirical scaling set *λ*_1_ = 1.0, *λ*_2_ = 0.445 so that both loss terms contributed equally. The *l*_WSD_ was realized by integrating the Sinkhorn loss^78^ that adds an entropy term to the WSD to accelerate differentiability.

We employed hyperparameter tuning via grid search^79^ and hyperband search^80^ with asynchronous scheduling. For SAE, MIDA, and STGAE, dimensions *d*_sae_, *d*_mida_, *d*_stgae_ were optimized with grid search alongside learning rate (lr), batch size (bz), dropout (dr), and weight decay for Adam optimizer wd. Due to higher computation costs of STAEs, we used hyperband optimization for lr, wd, and dr like before, in conjunction with the latent sizes *d*_stae_ and gradient accumulation steps (ag). Data with simulated missingness were filled with LOCF to retrieve the GT for our models to offer a fair comparison. Using available real GT data for fitting models was possible, but other approaches, like MICE, also assume simple imputations as a starting point. Using LOCF enables STAE to learn spatio-temporal dependencies while generalizing well. For comparison, we also used the known CCD for GT validation while training; these known values are otherwise concealed for all methods. Final model parameters are presented in Table B.2 in Supplement B.

For the training of model variants, graphics processing unit (GPU) jobs on a high-performance cluster allocated a maximum of four NVIDIA RTX A400 and were submitted via a queue manager. The average computation time during hyperparameter search with RayTune was ~7.5 h for one hyperparameter run per CV fold that optimized STAE architectures. The combined loss for STAE, which uses the WSD metric, was implemented via a Sinkhorn loss. Model versions that were configured with this loss were tuned for up to 14h due to the increased complexity of calculating this error metric. Table A.6 in Supplement A shows extensive ablations of model components that were iteratively added and removed across all three datasets. It was important to limit the hyperparameter search space as described before for retrieving tuning results within a moderate time span.

### Evaluation metrics

We assume GT data ${{{{\bf{Z}}}}}_{n}={({z}_{j,t})}_{n}$ and imputed data ${\bar{{{{\bf{X}}}}}}_{n}={({\bar{x}}_{j,t})}_{n}$ per patient *n*, feature *j*, and time *t* where imputations just affected simulated missing data for ${\hat{{{{\bf{R}}}}}}_{n}={({\hat{r}}_{j,t})}_{n}=1$. We calculated the normalized root-mean squared error (NRMSE) and the normalized mean absolute error (NMAE)^81^ for imputed values only with $I=\mathop{\sum }_{t=1}^{{\hat{l}}_{n}}{\hat{r}}_{j,t}$ as

2 $${{{{\rm{NRMSE}}}}}_{j,n}=\frac{\sqrt{\frac{1}{I}\mathop{\sum }_{t=1}^{{\hat{l}}_{n}}{({z}_{j,t}-{\bar{x}}_{j,t})}^{2}}}{{\mu }_{n}({z}_{j})}$$

3 $${{{{\rm{NMAE}}}}}_{j,n}=\frac{\frac{1}{I}\mathop{\sum }_{t=1}^{{l}_{n}}| {z}_{j,t}-{\bar{x}}_{j,t}| }{{\mu }_{n}({z}_{j})}$$

where *μ*(*z*_*j*_) in the denominator calculates the mean over GT feature values from one patient time series as a normalization term. In contrast to these two errors, we applied the normalized WSD (denoted with NWSD)^82^ as a metric comparing two distributions over all data (including non-imputed data) across all patients per single feature with

4 $${{{{\rm{NWSD}}}}}_{j}=\frac{\int_{-\infty }^{\infty }| {{{\rm{CDF}}}}({\bar{{{{\bf{X}}}}}}_{j}^{{\prime} })-{{{\rm{CDF}}}}({{{{\bf{Z}}}}}_{j}^{{\prime} })| }{\max ({{{{\bf{Z}}}}}_{j}^{{\prime} })-\min ({{{{\bf{Z}}}}}_{j}^{{\prime} })}$$

where ${{{{\bf{Z}}}}}_{j}^{{\prime} }$ and ${\bar{{{{\bf{X}}}}}}_{j}^{{\prime} }$ hold GT and imputed values per feature for the entire population with lengths of $\mathop{\sum }_{n=1}^{N}{\hat{l}}_{n}$. The cumulative density function CDF(*x*) = *P*(*X *≤ *x*) projects the probability of a random variable *X* being less than or equal to *x*. We additionally calculated NRMSE and NMAE globally for the entire population (see Additional Methods A.1 in Supplement A). To further quantify the changes in temporal dynamics by our imputations, we used auto-correlation error (ACorE) and cross-correlation error (CCorE) terms^83^. When the function Cov(*x*, *y*) calculates the covariance for two random numbers, we derived errors between ACor coefficients, calculated on GT and imputed data, for *L* time shifts (lags) per patient and feature as

5 $$	{{{{\rm{ACorE}}}}}_{j,n}=\frac{1}{L}\mathop{\sum }_{{\rm {lag}}=1}^{L}| {{{\rm{AC}}}}({z}_{j,t},{z}_{j,t-{\rm {lag}}})-{{{\rm{AC}}}}({\bar{x}}_{j,t},{\bar{x}}_{j,t-{\rm {lag}}})|,\,\\ 	 {{\rm{with}}}\,{{{\rm{AC}}}}(x,y)=\frac{{{{\rm{Cov}}}}(x,y)}{\sigma {(x)}^{2}}.$$

In contrast to the ACorE that describes the temporal correlations of single feature time series values with a time-shifted version of themselves, the CCorE further quantifies differences of spatial correlations between features *j* and ${j}^{{\prime} }$ as

6 $$	{{{{\rm{CCorE}}}}}_{n}=\frac{1}{{F}^{2}}\mathop{\sum}_{j=0}^{F}{\sum}_{{j}^{{\prime} }=0}^{F}\frac{1}{L}{\sum}_{{\rm {lag}}=1}^{L}| {{{\rm{CC}}}}({z}_{j},{z}_{{j}^{{\prime} }})-{{{\rm{CC}}}}({\bar{x}}_{j},{\bar{x}}_{{j}^{{\prime} }})|,\,\\ 	 {{\rm{with}}}\,{{{\rm{CC}}}}({x}_{t},{y}_{t})=\frac{{{{\rm{Cov}}}}({x}_{t},{y}_{t-1})}{\sigma ({x}_{t})\sigma ({y}_{t})}.$$

ACorE and CCorE range between 0 and 1. Metrics that were calculated per patient, such as NMAE, were called local errors. We refer to metrics that were calculated for the entire population, such as the NWSD, as global errors. While error metrics (MSE, RMSE, WSD) are naturally bounded by the valid data ranges that are used to detect and remove outliers in the original data, all reported metrics are normalized so that errors fall into a range between zero and one.

The RMSE, widely used in medical research studies^17,84^, describes the average clinical error magnitude that is directly interpretable for clinicians per single feature to reflect the acceptable range of imputation errors (e.g., RMSE of 500 mmHg for bp_sys). To make RMSE terms comparable across features, a normalization term was applied, so that the overall reported error metrics reflect the average deviation of imputed values from GT values across all five included parameters. ACorE and CCorE quantify the correlation of single time series values with temporally shifted versions, detecting changes in temporal dynamics for one single time series and across multiple ones^85^. Hereby, the temporal change between clinical parameters can be described per single time step (e.g., values of bp_sys depend 90% on its past values). Instead of quantifying pairwise errors such as the RMSE, the WSD^77^ measures the shifts in variance and skewness between distributions. Differences in distributions capture the preservation of clinical characteristics such as outliers and overall mean tendencies across the entire patient population, making them highly useful for descriptively analyzing the patient cohort^86^.

### Downstream classifier

For each dataset, we defined a downstream classification task. Specifically, we predicted in-hospital mortality for MIMIC and HIRID and postoperative delirium for ICDEP. In our previous work, we showed that an encoder-only TRAN architecture successfully predicts binary outcomes on the basis of clinical time series, outperforming LSTM or MLP approaches^16^. The TRAN model applies a linear layer on model inputs to expand the learnable latent space with a dimension of *d*_tran_ = {32, 64, or128}. After the linear layer, a single-head self-attention module learns from the dimensionally expanded input space using a feed-forward neural network (FFNN) with dimension *d*_fn_ = {32, or 64} to combine query, key, and value results. Afterward, another FFNN is applied to focus on temporal aspects with fixed dimensions of 4 nodes on each of the 4 layers. Hyperparameters *d*_tran_ and *d*_fn_ were tuned with Hyperband^80^, including batch sizes, learning rates, and weight decays into a searchable parameter space, similar to the STAE training procedure. We determined initial hyperparameters separately for MIMIC, HIRID, and ICDEP on CCD training data and applied the TRAN with a fixed configuration on various reconstructed sets to measure the importance of imputation methods. We trained and evaluated the model three times on the three imputed cross-validation hold-out sets, splitting them again into 66% train and 33% testing data. We applied 1000× bootstrapping on the testing sets to estimate 95% confidence intervals (CI). Models were trained for 150 epochs, while early stopping interrupted the training process when the testing loss increased by more than 10 epochs. Downstream classification performances were evaluated across amplification scenarios with the area under the receiver operating characteristic curve (AUROC) and the area under the precision-recall curve (AUPRC)^87^. In contrast to the previous study^16^, TRAN was configured as the main downstream classifier without comparison with MLPs or additional model variants. Implementation details are provided in our previous work^16^, final tuned parameters are included in Table B.3 in Supplement B to fully reproduce our results.

### Reporting summary

Further information on research design is available in the Nature Portfolio Reporting Summary linked to this article.

## Supplementary information

Supplementary Information

Peer Review file

Reporting Summary

## Source data

Source Data

## Data Availability

The pre-processed MIMIC-III v1.4^59^ and HIRID v1.1.1^41^ data are available under restricted access for data privacy reasons; access can be obtained by PhysioNet (https://physionet.org/content/mimiciii/1.4/, https://physionet.org/content/hirid/1.1.1/). After completing an ethics course at the PhysioNet platform, MIMIC and HIRID are accessible, requiring the submission of a research proposal. Permissions for secondary analysis of these databases are granted by the official PhysioNet Credentialed Health Data Use Agreement 1.5.0^58^. MIMIC as well as HIRID comply with the Health Insurance Portability and Accountability Act (HIPAA) by removing protected health information (e.g., names, addresses), shifting timestamps by a patient-specific offset, and applying binning techniques to age values. In contrast to MIMIC and HIRID, raw ICDEP data are protected and are not available due to data privacy laws. Here, we cannot fully mitigate all de-identification risks, violating fundamental data privacy rights according to German data privacy regulations under EU law^88^. However, pseudonymous ICDEP data can be requested from the corresponding author via niklas.giesa@charite.de. We require a detailed project description of how ICDEP data are going to be used and processed. The expected time frame for responses to access requests is two weeks. If data access has been granted, data will be available for the duration of the project for a maximum of three months. Source data for Fig. 2b, Figure 3b, and Figure 4a are provided with this paper. Source data are provided with this paper.

## Code availability

We share code, trained downstream models, and instructions for the application of our simulation model via GitHub (https://github.com/ngiesa/mi_icu). A code release file can be found under the DIO 10.5281/zenodo.21356282^57^. After data retrieval, this GitHub code can be used to fully reproduce our results. Data were analyzed with the following Python 3.8 packages: numpy 1.24.4, statsmodels 0.13.5, pandas 2.0.3, sklearn 0.0.1, and Pytorch lightning 1.6.5.0, torch 2.4.1, autograd 1.5. To install the exact versions of packages, please use the provided requirements.txt^57^.
