# Author Correction: Benchmarking imputation methods on real-world clinical time series with simulated spatio-temporal missingness

**DOI:** 10.1038/s41467-026-79464-y

**Published:** 2026-11-06

**Authors:** Niklas Giesa, Rustam Zhumagambetov, Maria Sekutowicz, Sophie K. Piper, Victor Sikora, Felix Balzer, Stefan Haufe, Sebastian D. Boie

**Affiliations:** 1 Institute of Medical Informatics (IMI), Charité - Universitätsmedizin Berlin, corporate member of Freie Universität Berlin and Humboldt-Universität zu Berlin, Berlin, Germany; 2 Mathematical Modelling and Data Analysis Department, Physikalisch-Technische Bundesanstalt (PTB), Berlin, Germany; 3 Department of Anesthesiology and Intensive Care Medicine, Charité - Universitätsmedizin Berlin, corporate member of Freie Universität Berlin and Humboldt-Universität zu Berlin, Berlin, Germany; 4 Institute of Biometry and Clinical Epidemiology, Charité - Universitätsmedizin Berlin, corporate member of Freie Universität Berlin and Humboldt-Universität zu Berlin, Berlin, Germany; 5 Department of Infectious Diseases, Respiratory Medicine and Critical Care Medicine, Charité - Universitätsmedizin Berlin, corporate member of Freie Universität Berlin and Humboldt-Universität zu Berlin, Berlin, Germany; 6 Uncertainty, Inverse Modeling and Machine Learning Group, Technische Universität Berlin, Berlin, Germany

**Keywords:** Outcomes research, Data processing, Predictive medicine

Correction to: *Nature Communications* https://doi.org/10.1038/s41467-026-77303-8, published online 5 October 2026

In the version of the article initially published, the surname of Maria Sekutowicz appeared incorrectly (as Sekutowitcz) and has now been corrected in the HTML and PDF versions of the article.

