# Supplementary material for: Benchmarking imputation methods on real-world clinical time series with simulated spatio-temporal missingness
Source: Nat Commun. 2026 Oct 5;17:10491. doi: 10.1038/s41467-026-77303-8 (PMC13639038; doi:10.1038/s41467-026-77303-8)
Supplement: Supplementary file 1 [file 41467_2026_77303_MOESM1_ESM.pdf]

A Supplementary Information

Benchmarking imputation methods on real-world clinical time series with simulated spatio-temporal missingness

by Niklas Giesa, Rustam Zhumagambetov, Maria Sekutowitcz, Victor Sikora, Sophie K. Piper, Felix Balzer, Stefan Haufe, Sebastian D. Boie

Dataset	Feature	Orig. Sampling	2x Sampling	3x Sampling	4x Sampling	5x Sampling	6x Sampling	7x Sampling	8x Sampling
MIMIC	hr	0.104	0.062	0.053	0.048	0.046	0.044	0.042	0.041
	rr	0.118	0.074	0.063	0.056	0.053	0.051	0.048	0.047
	bp_sys	0.133	0.072	0.059	0.052	0.049	0.047	0.045	0.044
	bp_dia	0.133	0.072	0.059	0.052	0.049	0.047	0.045	0.044
	bp_map	0.137	0.077	0.063	0.056	0.052	0.050	0.048	0.047
	spo2	0.144	0.088	0.071	0.062	0.057	0.054	0.051	0.050
	temp	0.705	0.499	0.320	0.170	0.134	0.115	0.103	0.095
HIRID	hr	0.011	0.008	0.007	0.006	0.006	0.006	0.006	0.005
	spo2	0.043	0.034	0.029	0.025	0.023	0.020	0.019	0.017
	bp_map	0.074	0.054	0.041	0.033	0.027	0.021	0.019	0.017
	bp_dia	0.074	0.054	0.041	0.033	0.027	0.021	0.019	0.017
	bp_sys	0.075	0.054	0.041	0.033	0.027	0.021	0.019	0.017
	rr	0.403	0.222	0.206	0.190	0.200	0.167	0.139	0.114
	temp	0.872	0.849	0.827	0.805	0.784	0.763	0.743	0.724
ICDEP	hr	0.058	0.042	0.040	0.039	0.038	0.037	0.037	0.036
	spo2	0.065	0.047	0.044	0.043	0.041	0.041	0.041	0.039
	rr	0.155	0.136	0.129	0.124	0.120	0.118	0.115	0.112
	bp_dia	0.422	0.218	0.140	0.108	0.090	0.085	0.082	0.076
	bp_sys	0.423	0.224	0.150	0.120	0.104	0.100	0.096	0.089
	bp_map	0.425	0.227	0.152	0.122	0.106	0.101	0.097	0.090
	temp	0.575	0.563	0.552	0.543	0.532	0.523	0.512	0.502

Table A.1. Feature missingness rates per dataset and feature across original and resampled datasets For each dataset, the fraction of all missing values per feature across all patient time series was calculated as feature missingness FM per original (Orig.) as well as for seven resampled sets that used (2, 3, ..., 8x) multiples of the original sampling intervals 60, 10, and 5 min. for MIMIC, HIRID, or ICDEP (e.g., {120, 180, ..., 480} min. for MIMIC). Seven common features as heart rate (hr), systolic, diastolic and mean blood pressure (bp_sys, bp_dia, bp_map), respiratory rate (rr), blood oxygen (spo2), and the body temperature (temp) were selected. The table is sorted ascending by dataset and original FM rates.

Dataset	Feature	Affected	Original data	CCD outlier corr.	CDD outlier rem.	Sim. data outlier corr.	Sim. data outlier rem.
MIMIC	hr	10.41%	84.95±17.26	84.94±17.70	84.88±17.68	84.92±17.70	84.89±17.7
	bp_sys	13.25%	121.82±22.00	120.54±21.71	120.56±21.60	120.54±21.72	120.58±21.60
	bp_dia	13.27%	60.89±14.13	60.44±13.85	60.47±13.84	60.44±13.86	60.48±13.88
	spo2	14.38%	96.72±3.59	96.98±3.19	96.98±3.14	96.98±3.20	96.98±3.20
	rr	11.83%	19.09±5.72	18.65±5.69	18.69±5.62	18.66±5.70	18.69±5.60
HIRID	hr	0.66%	86.36±17.46	86.11±16.80	86.08±16.73	86.11±16.81	86.11±16.72
	bp_sys	3.93%	116.73±25.62	109.78±23.24	109.48±22.99	109.78±23.25	109.74±29.99
	bp_dia	3.93%	57.80±12.82	55.09±11.19	55.09±11.13	55.08±11.20	55.09±11.12
	spo2	2.54%	96.42±3.39	97.03±3.29	97.15±3.19	97.04±3.30	97.15±3.19
	rr	20.54%	24.68±25.63	25.42±29.60	21.36±24.23	25.42±29.60	21.35±24.19
ICDEP	hr	4.22%	65.50±14.28	64.42±13.71	64.23±13.59	63.74±13.71	64.22±13.58
	bp_sys	22.44%	112.59±20.27	111.54±20.17	111.22±20.00	111.81±20.66	111.20±19.99
	bp_dia	21.76%	65.91±13.15	77.53±14.03	77.38±13.89	65.13±13.70	77.35±13.83
	spo2	4.74%	98.87±1.61	99.01±1.41	99.01±1.42	99.07±1.39	99.07±1.42
	rr	13.60%	11.94±4.89	11.81±4.67	11.87±3.06	11.62±5.48	11.88±3.08

Table A.2. Summary statistics across original, complete, and simulated sets with two cleansing strategies. Statistics are reported as mean±std globally for the entire population per feature and dataset. The fraction of feature values that were affected by our cleaning is included (affected), here values did not fall into clinical valid ranges. If so, these values were either corrected (corr.) via setting them to valid range edges or removed (rem.) from the set by setting them to missing (see Methods in main manuscript). Results are cited for resampled original, complete case (CCD), and simulated (sim.) data.

	MIMIC			HIRID			ICDEP		
	Feature Missingness	Block Missingness	Single Missingness	Feature Missingness	Block Missingness	Single Missingness	Feature Missingness	Block Missingness	Single Missingness
Original data									
hr	0.104	0.079±0.116	0.101±0.137	0.006	0.013±0.027	0.014±0.029	0.047	0.028±0.065	0.029±0.067
bp_sys	0.132	0.084±0.117	0.175±0.148	0.039	0.023±0.034	0.041±0.076	0.235	0.133±0.099	0.197±0.137
bp_dia	0.132	0.084±0.117	0.175±0.148	0.039	0.023±0.034	0.041±0.076	0.235	0.134±0.101	0.198±0.139
spo2	0.143	0.099±0.122	0.121±0.175	0.025	0.023±0.044	0.036±0.072	0.049	0.030±0.080	0.021±0.083
rr	0.118	0.094±0.138	0.178±0.172	0.266	0.115±0.127	0.241±0.341	0.113	0.103±0.344	0.121±0.357
Simulated data									
hr	0.098	0.072±0.113	0.087±0.126	0.003	0.012±0.013	0.012±0.013	0.023	0.021±0.099	0.022±0.100
bp_sys	0.115	0.089±0.119	0.161±0.136	0.033	0.023±0.043	0.037±0.051	0.218	0.143±0.140	0.190±0.169
bp_dia	0.115	0.089±0.119	0.162±0.136	0.033	0.023±0.043	0.037±0.051	0.218	0.143±0.142	0.190±0.161
spo2	0.125	0.100±0.132	0.127±0.151	0.021	0.018±0.044	0.033±0.051	0.022	0.023±0.102	0.023±0.103
rr	0.118	0.086±0.124	0.120±0.139	0.251	0.118±0.135	0.253±0.155	0.114	0.109±0.246	0.111±0.249

Table A.3. Missingness statistics for resampled original and simulated data. Differences between resampled original and complete case data (CDD) is shown for feature missingness FM as global fraction of all missing values, block missingness BM as consecutive unit of missing values within one local time series, and single missingness SM as missing values from all within one local time series. For FM and BM, we report mean±standard-deviation calculated across patient time series.

feature	unit	bin 0	bin 1	bin 2
hr	1/min	(10,77]	(77,91]	(91,300]
bp_sys	mmHg	(20,104]	(104,127]	(127,300]
bp_dia	mmHg	(10,51]	(51,62]	(62,200]
spo2	%	(20,96]	(98,100]	(98,100]
rr	1/min	(5,15]	(15,22]	(22,200]

Table A.4. Value bins per feature calculated on complete case data. Equally sized bins were derived from complete case data (CCD) across all datasets MIMIC, HIRID, ICDEP. Each data point could be assigned to one bin. Afterwards, missingness could be induced according to the bin levels for amplifying MNAR effects.

	ACorE$_{loc}$	ACorWSD$_{loc}$	CCorE$_{loc}$	NMAE$_{loc}$	NRMSE$_{loc}$	NWSD$_{loc}$	NMAE$_{glob}$	NRMSE$_{glob}$	NWSD$_{glob}$
MIMIC									
Non Time Causal Methods									
PMEAN	9.373±0.065	4.191±0.028	9.769±0.195	15.166±0.131	16.256±0.135	49.597±0.775	14.933±4.030	19.106±5.105	0.529±0.105
PMEDI	9.320±0.065	4.170±0.027	9.666±0.192	14.853±0.126	15.967±0.131	48.629±0.749	14.842±3.989	19.202±5.120	0.527±0.105
LINPOL	6.869±0.060	3.237±0.025	6.581±0.164	9.375±0.094	10.578±0.103	24.798±0.329	9.213±2.981	13.369±4.039	0.090±0.016
NEAPOL	7.411±0.061	3.345±0.024	7.383±0.175	10.877±0.107	12.280±0.116	29.650±0.468	10.550±3.315	15.193±4.496	0.106±0.019
QUAPOL	8.107±0.066	3.682±0.026	8.060±0.179	12.019±0.121	13.429±0.132	32.012±0.438	12.444±4.098	18.137±5.681	0.096±0.013
MICTRE	11.334±0.067	4.842±0.032	12.738±0.211	21.278±0.171	23.687±0.184	64.169±1.051	21.045±5.737	26.969±7.229	0.014±0.003
MICKNN	9.508±0.065	4.209±0.028	10.048±0.198	15.435±0.130	16.641±0.135	48.552±0.768	15.331±4.107	19.760±5.212	0.471±0.104
SAE	9.387±0.065	4.191±0.027	9.858±0.198	15.160±0.130	16.251±0.135	49.623±0.777	14.935±4.029	19.115±5.107	0.529±0.105
MIDA	9.395±0.065	4.185±0.027	9.846±0.197	15.172±0.131	16.265±0.135	49.440±0.780	14.937±4.032	19.111±5.107	0.518±0.103
BILSTAE	7.180±0.059	3.290±0.023	6.581±0.159	9.794±0.090	10.840±0.097	28.718±0.383	9.822±2.869	13.442±3.777	0.234±0.041
Time Causal Methods									
1MEAN	9.444±0.065	4.219±0.028	9.852±0.195	15.608±0.135	16.695±0.139	50.775±0.808	15.248±4.079	19.397±5.188	0.541±0.112
1MEDI	9.360±0.064	4.189±0.028	9.696±0.192	15.237±0.130	16.333±0.134	48.492±0.734	15.111±4.083	19.406±5.218	0.535±0.108
RMEAN	7.436±0.057	3.514±0.024	6.663±0.162	10.631±0.096	11.798±0.104	28.893±0.334	10.727±3.098	14.682±4.084	0.131±0.023
RMEDI	7.494±0.058	3.530±0.025	6.692±0.162	10.644±0.098	11.873±0.107	28.886±0.338	10.752±3.118	14.930±4.162	0.117±0.020
MICET	11.385±0.067	4.892±0.032	12.739±0.211	11.581±0.042	9.283±0.081	12.295±0.289	21.207±5.732	27.211±7.244	0.032±0.010
LOCF	7.256±0.060	3.377±0.025	7.041±0.164	10.557±0.105	11.862±0.114	27.868±0.346	10.468±3.326	14.999±4.528	0.062±0.012
LINEXT	8.916±0.065	3.958±0.026	9.390±0.181	15.546±0.163	17.145±0.173	39.636±0.398	15.449±5.159	22.142±7.132	0.131±0.023
QUAEXT	10.954±0.066	4.884±0.032	13.658±0.223	26.730±0.312	29.242±0.331	65.566±0.595	26.979±9.326	40.376±13.442	0.459±0.092
LSTAE	7.539±0.060	3.434±0.023	7.107±0.168	10.684±0.098	11.775±0.105	31.616±0.440	10.645±3.058	14.429±4.002	0.265±0.045
STGAE	9.456±0.065	4.214±0.028	9.843±0.194	15.449±0.134	16.544±0.138	49.672±0.772	15.123±4.073	19.293±5.129	0.454±0.096
HIRID									
Non Time Causal Methods									
PMEAN	8.287±0.100	7.198±0.119	6.610±0.105	40.518±0.579	43.133±0.596	55.141±9.350	24.308±14.025	43.705±31.908	0.379±0.334
PMEDI	6.557±0.076	5.855±0.090	4.893±0.091	23.726±0.261	27.152±0.305	33.815±2.385	21.524±11.096	44.773±32.921	0.322±0.272
LINPOL	3.758±0.065	2.837±0.048	3.384±0.090	13.670±0.241	17.824±0.296	17.304±9.159	9.973±7.695	27.846±24.283	0.026±0.024
NEAPOL	4.075±0.069	2.956±0.049	3.596±0.093	15.986±0.278	20.892±0.337	20.571±9.174	11.092±8.305	31.412±27.134	0.021±0.019
QUAPOL	5.719±0.094	4.336±0.081	4.726±0.097	24.553±0.448	30.984±0.567	25.203±9.169	23.055±21.469	76.982±77.908	0.218±0.246
MICTRE	11.162±0.125	11.202±0.194	8.288±0.115	54.458±0.904	94.193±1.828	65.536±5.559	33.370±18.598	61.462±44.623	0.008±0.007
MICKNN	8.260±0.102	7.834±0.144	6.060±0.103	34.432±0.509	47.676±0.777	47.712±13.231	24.681±13.844	44.382±31.728	0.145±0.115
SAE	8.274±0.100	7.187±0.119	6.597±0.105	40.401±0.577	43.020±0.595	54.978±9.308	24.281±13.999	43.704±31.908	0.379±0.333
MIDA	8.286±0.100	7.180±0.119	6.662±0.107	40.511±0.579	43.127±0.596	55.099±9.434	24.314±14.031	43.711±31.915	0.378±0.333
BILSTAE	3.900±0.075	3.274±0.080	3.348±0.091	16.193±0.322	18.685±0.364	18.403±1.760	12.626±11.408	37.102±34.858	0.337±0.385
Time Causal Methods									
1MEAN	6.435±0.075	5.932±0.095	4.719±0.092	22.620±0.226	26.046±0.276	32.732±0.802	22.175±11.476	45.406±33.669	0.333±0.281
1MEDIAN	6.361±0.077	5.964±0.099	4.618±0.093	22.329±0.225	25.769±0.277	29.536±0.652	22.284±11.664	45.754±33.788	0.338±0.285
RMEAN	4.369±0.058	3.811±0.061	3.370±0.079	16.341±0.187	19.542±0.233	18.137±0.438	15.132±8.942	31.033±23.402	0.116±0.112
RMEDI	4.613±0.064	4.081±0.071	3.617±0.082	16.249±0.197	20.406±0.256	17.906±0.442	15.182±9.226	33.394±26.014	0.141±0.144
MICET	9.397±0.101	9.103±0.152	6.600±0.108	5.878±0.104	17.047±0.278	6.765±2.692	28.247±13.037	52.265±33.994	0.160±0.172
LOCF	3.795±0.060	2.899±0.044	3.164±0.081	13.033±0.180	17.280±0.242	12.302±0.438	10.859±7.545	30.560±25.862	0.010±0.008
LINEXT	5.157±0.080	3.893±0.063	4.188±0.090	20.315±0.320	25.686±0.399	17.699±0.467	18.449±14.867	52.612±48.338	0.137±0.148
QUAEXT	7.397±0.102	6.051±0.102	5.870±0.101	37.689±0.651	46.389±0.796	31.540±0.594	36.160±31.275	104.830±100.300	0.393±0.423
LSTAE	5.161±0.074	4.151±0.071	4.588±0.096	20.389±0.274	23.791±0.317	25.173±5.186	16.087±10.664	34.695±28.093	0.177±0.168
STGAE	7.571±0.089	6.559±0.101	5.892±0.100	32.813±0.444	35.975±0.471	42.911±4.480	22.932±12.255	44.873±32.911	0.452±0.439
ICDEP									
Non Time Causal Methods									
PMEAN	11.924±0.095	4.998±0.050	8.789±0.155	14.384±0.122	15.561±0.128	43.754±0.444	12.558±3.369	17.200±4.865	0.438±0.216
PMEDI	11.379±0.085	4.591±0.033	8.388±0.151	14.137±0.123	15.359±0.130	42.229±0.423	12.443±3.361	17.383±4.884	0.432±0.213
LINPOL	10.048±0.098	4.549±0.052	7.555±0.156	11.264±0.118	12.539±0.128	30.163±0.326	10.701±3.036	15.941±4.876	0.184±0.069
NEAPOL	10.724±0.096	4.655±0.051	8.108±0.156	12.824±0.125	14.244±0.135	34.553±0.367	11.573±3.175	16.867±4.942	0.215±0.078
QUAPOL	10.812±0.099	4.729±0.052	8.012±0.157	12.637±0.125	13.951±0.135	34.213±0.359	11.799±3.300	17.214±5.155	0.197±0.072
MICTRE	13.530±0.090	4.864±0.031	12.211±0.184	20.017±0.161	22.102±0.182	58.045±0.737	17.731±4.820	24.253±7.096	0.017±0.005
MICKNN	12.163±0.092	4.768±0.040	9.484±0.170	14.880±0.126	16.223±0.135	43.153±0.479	13.462±3.704	18.269±5.237	0.301±0.143
SAE	11.914±0.095	4.985±0.049	8.769±0.154	14.401±0.122	15.578±0.128	43.830±0.446	12.559±3.368	17.199±4.865	0.438±0.216
MIDA	11.763±0.086	4.723±0.033	9.250±0.164	14.359±0.121	15.538±0.128	43.657±0.443	12.552±3.366	17.211±4.867	0.436±0.215
BILSTAE	10.319±0.081	4.340±0.033	8.166±0.156	11.885±0.110	13.040±0.119	33.173±0.318	10.655±2.956	15.187±4.562	0.281±0.132
Time Causal Methods									
1MEAN	12.380±0.092	5.009±0.043	9.064±0.153	16.357±0.128	17.423±0.133	51.232±0.538	14.401±4.005	18.489±5.356	0.491±0.242
1MEDIAN	11.913±0.087	4.749±0.034	8.735±0.149	15.696±0.125	16.783±0.131	48.459±0.508	14.065±3.992	18.300±5.338	0.472±0.228
RMEAN	10.992±0.088	4.702±0.043	7.945±0.152	13.681±0.127	14.870±0.134	36.815±0.366	12.857±3.749	17.716±5.505	0.210±0.075
RMEDI	10.915±0.089	4.679±0.043	7.970±0.153	13.584±0.129	14.815±0.136	36.594±0.373	12.728±3.672	17.774±5.485	0.196±0.067
MICET	13.553±0.090	4.874±0.031	12.371±0.184	5.829±0.075	11.825±0.130	16.444±0.401	18.393±5.102	25.431±7.807	0.051±0.020
LOCF	10.265±0.092	4.482±0.044	7.764±0.154	12.953±0.130	14.247±0.139	34.899±0.381	12.289±3.520	17.668±5.406	0.161±0.050
LINEXT	11.547±0.095	4.795±0.044	8.609±0.157	17.020±0.172	18.455±0.180	45.241±0.439	14.673±4.056	21.042±6.139	0.182±0.066
QUAEXT	12.733±0.093	5.140±0.043	9.898±0.164	23.447±0.277	25.231±0.288	61.778±0.645	18.172±5.052	27.542±8.029	0.324±0.163
LSTAE	10.794±0.084	4.484±0.033	8.553±0.161	12.697±0.114	13.877±0.122	36.231±0.370	11.472±3.127	16.153±4.691	0.319±0.146
STGAE	12.028±0.090	4.870±0.040	9.125±0.159	15.050±0.128	16.225±0.135	46.145±0.524	13.184±3.530	17.811±5.014	0.417±0.209

Table A.5. Performance metrics for imputation methods with no amplification. The performance of 20 imputation methods is shown for the no amplification. We display the local autocorrelation error (ACorE$_{loc}$), Wasserstein distance thereof (ACorWSD$_{loc}$), cross correlation divergence (CCorE$_{loc}$), normalized mean absolute error (NMAE$_{loc}$), normalized root-mean squared error (NRMSE$_{loc}$), and the local Wasserstein distance (NWSD$_{loc}$). NMAE, NRMSE, and WSD were also derived globally (NMAE$_{glob}$, NRMSE$_{glob}$). We report all errors in % with mean±95%CI across time series (14,286, 11,286, and 17,811 for MIMIC, HIRID, ICDEP), 5 features, and 3 validation folds.

Ablation Parameters				Performance Metrics								
Static Attrib.	Comb. Loss	LOCF pre-imp.		$ACorE_{loc}$	$ACorWSD_{loc}$	$CCorE_{loc}$	$NMAE_{loc}$	$NRMSE_{loc}$	$NWSD_{loc}$	$NMAE_{glob}$	$NRMSE_{glob}$	$NWSD_{glob}$
			MIMIC									
			Non Time Causal Methods									
			LINPOL (baseline)	6.869±0.060	3.237±0.025	6.581±0.164	9.375±0.094	10.578±0.103	24.798±0.329	9.213±2.981	13.369±4.039	0.090±0.016
			BILSTAE	7.263±0.059	3.323±0.022	6.555±0.156	9.987±0.091	11.035±0.098	29.432±0.388	9.967±2.895	13.548±3.804	0.229±0.042
X			BILSTAE_STAT	7.067±0.058	3.253±0.022	6.351±0.154	9.556±0.087	10.594±0.094	27.598±0.343	9.656±2.832	13.205±3.725	0.231±0.039
		X	BILSTAE_LOC	7.180±0.059	3.290±0.023	6.581±0.159	9.794±0.090	10.840±0.097	28.718±0.383	9.822±2.869	13.442±3.777	0.234±0.041
	X		BILSTAE_WSD	7.144±0.059	3.275±0.023	6.465±0.154	9.633±0.088	10.686±0.095	27.457±0.348	9.778±2.887	13.339±3.781	0.230±0.039
X	X		BILSTAE_STAT_WSD	7.123±0.059	3.267±0.023	6.429±0.155	9.647±0.088	10.690±0.096	27.788±0.351	9.796±2.861	13.383±3.767	0.216±0.039
X		X	BILSTAE_LOC_STA	7.140±0.058	3.274±0.022	6.442±0.156	9.721±0.089	10.765±0.096	28.364±0.364	9.733±2.842	13.301±3.741	0.216±0.036
	X	X	BILSTAE_LOC_WSD	7.296±0.060	3.325±0.023	6.718±0.160	9.952±0.090	11.014±0.097	28.963±0.392	9.966±2.894	13.565±3.798	0.212±0.037
			Time Causal Methods									
			1LOCF (baseline)	7.256±0.060	3.377±0.025	7.041±0.164	10.557±0.105	11.862±0.114	27.868±0.346	10.468±3.326	14.999±4.528	0.062±0.012
			LSTAE	7.463±0.059	3.404±0.023	6.893±0.162	10.462±0.095	11.544±0.102	30.870±0.422	10.891±3.095	14.711±4.057	0.263±0.045
X			LSTAE_STAT	7.678±0.060	3.487±0.023	7.262±0.170	10.933±0.100	12.023±0.106	32.794±0.453	10.831±3.095	14.616±4.042	0.266±0.047
			LSTAE_LOC	7.539±0.060	3.434±0.023	7.107±0.168	10.684±0.098	11.775±0.105	31.616±0.440	10.645±3.058	14.429±4.002	0.265±0.045
	X		LSTAE_WSD	7.433±0.060	3.385±0.023	6.906±0.163	10.384±0.096	11.468±0.103	30.295±0.412	10.607±3.031	14.408±3.983	0.232±0.040
X	X		LSTAE_STAT_WSD	7.854±0.061	3.536±0.024	7.462±0.171	11.375±0.103	12.491±0.110	33.267±0.440	11.259±3.315	14.981±4.205	0.283±0.059
X		X	LSTAE_LOC_STAT	7.676±0.060	3.482±0.023	7.278±0.170	10.912±0.100	12.010±0.106	32.709±0.457	10.845±3.089	14.617±4.038	0.269±0.047
	X	X	LSTAE_LOC_WSD	7.944±0.061	3.586±0.024	7.631±0.173	11.456±0.102	12.573±0.108	34.360±0.485	11.261±3.177	15.047±4.129	0.242±0.045
			GRUAE	7.868±0.061	3.560±0.024	7.640±0.176	11.324±0.102	12.419±0.109	34.699±0.485	11.296±3.223	15.104±4.192	0.315±0.055
			HIRID									
			Non Time Causal Methods									
			LINPOL (baseline)	3.758±0.065	2.837±0.048	3.384±0.090	13.670±0.241	17.824±0.296	17.304±9.159	9.973±7.695	27.846±24.283	0.026±0.024
			BILSTAE	4.731±0.067	3.852±0.065	3.914±0.086	18.303±0.240	21.338±0.279	24.261±6.578	15.678±10.010	35.302±28.653	0.182±0.164
X			BILSTAE_STAT	5.048±0.072	4.121±0.070	4.300±0.093	19.808±0.276	22.924±0.314	27.486±9.153	16.255±10.606	35.781±29.190	0.193±0.172
		X	BILSTAE_LOC	3.900±0.075	3.274±0.080	3.348±0.091	16.193±0.322	18.685±0.364	18.403±1.760	12.626±11.408	37.102±34.858	0.337±0.385
	X		BILSTAE_WSD	4.354±0.063	3.444±0.055	3.707±0.085	16.800±0.215	19.642±0.252	21.642±4.153	14.587±9.212	32.827±26.459	0.154±0.143
X	X		BILSTAE_STAT_WSD	4.768±0.072	3.770±0.063	4.124±0.089	18.591±0.257	21.706±0.297	22.817±4.948	14.945±10.093	33.015±26.974	0.177±0.169
X		X	BILSTAE_LOC_STA	5.202±0.076	4.236±0.076	4.548±0.094	20.858±0.293	24.204±0.335	27.167±7.225	16.123±11.165	35.179±29.083	0.183±0.175
	X	X	BILSTAE_LOC_WSD	4.663±0.068	3.687±0.060	4.109±0.091	18.325±0.244	21.381±0.283	23.217±5.156	15.205±10.027	33.262±27.010	0.165±0.155
			Time Causal Methods									
			1LOCF (baseline)	3.795±0.060	2.899±0.044	3.164±0.081	13.033±0.180	17.280±0.242	12.302±0.438	10.859±7.545	30.560±25.862	0.010±0.008
			LSTAE	4.950±0.070	3.929±0.063	4.380±0.095	19.468±0.257	22.662±0.298	24.673±4.754	15.586±9.964	34.132±27.410	0.182±0.170
X			LSTAE_STAT	5.576±0.075	4.626±0.077	4.654±0.094	21.968±0.290	25.200±0.329	28.735±4.753	17.527±11.099	37.314±30.134	0.219±0.203
		X	LSTAE_LOC	5.161±0.074	4.151±0.071	4.588±0.096	20.389±0.274	23.791±0.317	25.173±5.186	16.087±10.664	34.695±28.093	0.177±0.168
	X		LSTAE_WSD	4.794±0.067	3.796±0.058	4.386±0.096	18.679±0.237	21.964±0.278	25.287±7.816	15.167±9.595	33.455±26.695	0.146±0.143
X	X		LSTAE_STAT_WSD	5.110±0.073	4.074±0.068	4.472±0.096	20.271±0.270	23.541±0.312	25.164±4.738	15.982±10.371	34.436±27.751	0.192±0.181
X		X	LSTAE_LOC_STAT	5.656±0.077	4.667±0.079	4.783±0.096	22.357±0.302	25.560±0.338	28.837±5.064	17.347±10.814	36.909±29.404	0.224±0.214
	X	X	LSTAE_LOC_WSD	4.778±0.068	3.786±0.060	4.226±0.094	18.594±0.237	21.691±0.276	24.377±6.418	15.233±9.554	33.270±26.487	0.166±0.154
			GRUAE	5.567±0.078	4.579±0.080	4.718±0.094	22.115±0.312	25.439±0.351	26.151±2.212	17.281±11.331	36.930±29.874	0.197±0.183
			ICDEP									
			Non Time Causal Methods									
			LINPOL (baseline)	10.048±0.098	4.549±0.052	7.555±0.156	11.264±0.118	12.539±0.128	30.163±0.326	10.701±3.036	15.941±4.876	0.184±0.069
			BILSTAE	9.777±0.079	4.122±0.030	7.531±0.146	11.220±0.105	12.352±0.114	30.775±0.292	10.193±2.832	14.686±4.440	0.258±0.120
X			BILSTAE_STAT	10.189±0.082	4.290±0.033	8.062±0.157	11.596±0.108	12.755±0.117	32.037±0.304	10.533±2.922	15.103±4.525	0.271±0.125
		X	BILSTAE_LOC	10.319±0.081	4.340±0.033	8.166±0.156	11.885±0.110	13.040±0.119	33.173±0.318	10.655±2.956	15.187±4.562	0.281±0.132
	X		BILSTAE_WSD	10.390±0.085	4.390±0.035	8.134±0.159	11.675±0.109	12.846±0.118	32.080±0.309	10.647±2.961	15.223±4.552	0.262±0.119
X	X		BILSTAE_STAT_WSD	10.549±0.084	4.396±0.033	8.105±0.152	12.189±0.110	13.324±0.118	34.444±0.347	10.981±3.033	15.429±4.582	0.279±0.133
X		X	BILSTAE_LOC_STA	10.777±0.085	4.459±0.033	8.683±0.160	12.692±0.116	13.862±0.124	35.559±0.351	11.173±3.112	15.739±4.657	0.298±0.150
	X	X	BILSTAE_LOC_WSD	10.491±0.084	4.309±0.032	7.969±0.148	12.243±0.109	13.411±0.118	34.480±0.363	10.926±3.012	15.407±4.572	0.253±0.116
			Time Causal Methods									
			1LOCF (baseline)	10.265±0.092	4.482±0.044	7.764±0.154	12.953±0.130	14.247±0.139	34.899±0.381	12.289±3.520	17.668±5.406	0.161±0.050
			LSTAE	10.578±0.082	4.383±0.032	8.236±0.156	12.472±0.113	13.634±0.121	35.528±0.367	11.274±3.081	15.907±4.641	0.309±0.145
X			LSTAE_STAT	10.471±0.081	4.312±0.030	8.117±0.153	12.446±0.112	13.595±0.120	35.511±0.368	11.220±3.060	15.812±4.613	0.303±0.145
		X	LSTAE_LOC	10.794±0.084	4.484±0.033	8.553±0.161	12.697±0.114	13.877±0.122	36.231±0.370	11.472±3.127	16.153±4.691	0.319±0.146
	X		LSTAE_WSD	10.619±0.084	4.417±0.033	8.227±0.156	12.486±0.112	13.666±0.121	35.118±0.365	11.354±3.113	15.968±4.660	0.283±0.129
X	X		LSTAE_STAT_WSD	10.432±0.082	4.229±0.030	8.048±0.150	12.526±0.112	13.685±0.120	35.744±0.387	11.287±3.084	15.859±4.621	0.271±0.124
X		X	LSTAE_LOC_STAT	10.613±0.083	4.394±0.032	8.302±0.156	12.547±0.113	13.699±0.121	35.725±0.364	11.319±3.086	15.919±4.635	0.310±0.146
	X	X	LSTAE_LOC_WSD	10.559±0.083	4.345±0.032	8.350±0.159	12.521±0.114	13.698±0.122	34.988±0.367	11.346±3.117	15.987±4.669	0.274±0.122
			GRUAE	11.001±0.084	4.540±0.033	8.642±0.162	13.075±0.115	14.244±0.123	38.006±0.391	11.706±3.180	16.335±4.719	0.343±0.161

Table A.6. Ablations for STAE architectures. We trained different spatio-temporal denoising autoencoder (STAE) comprising (bidirectional) long-short term memory (Bi)LSTM modules for capturing temporal dynamics or gated recurrent units (GRUs). We compared model performances against linear interpolation (LINPOL) and last observation carried forward (LOCF) as baselines. Model were either trained with real complete case data or pre-imputed (LOCF pre-imp.) data for ground truth (GT) validations. We also integrated a combined loss term (Comp. Loss) jointly calculating the mean absolute error (MAE) and the Wasserstein distance (WSD). Models were also fitted with static attributes like gender and age (Static Attrib.) that were used in the encoder only to potentially improve imputation performance. We explained performance metrics inMethods of the main manuscript. We report all errors in % with mean±95%CI across time series (14,286, 11,286, and 17,811 for MIMIC, HIRID, ICDEP), 5 features, and 3 validation folds.

	$ACorE_{loc}$	$ACorWSD_{loc}$	$CCorE_{loc}$	$NMAE_{loc}$	$NRMSE_{loc}$	$NWSD_{loc}$	$NMAE_{glob}$	$NRMSE_{glob}$	$NWSD_{glob}$
MIMIC									
Non Time Causal Methods									
PMEAN	13.419±0.064	5.787±0.037	20.072±0.295	15.367±0.109	16.947±0.115	54.111±0.737	14.935±4.111	19.206±5.285	1.613±0.335
PMEDI	13.268±0.063	5.681±0.034	19.680±0.288	15.063±0.105	16.675±0.111	53.182±0.714	14.923±4.080	19.401±5.320	1.615±0.338
LINPOL	11.833±0.073	5.617±0.038	15.088±0.272	9.891±0.078	11.727±0.089	26.682±0.349	9.497±3.104	13.691±4.178	0.295±0.053
NEAPOL	15.682±0.066	6.017±0.038	19.897±0.238	21.262±0.131	24.755±0.148	64.699±0.791	20.836±5.729	26.730±7.243	0.255±0.135
QUAPOL	13.255±0.075	6.120±0.039	17.158±0.277	12.599±0.100	14.773±0.115	33.541±0.409	12.839±4.298	18.608±6.057	0.335±0.052
MICTRE	15.682±0.066	6.017±0.038	19.897±0.238	21.262±0.131	24.755±0.148	64.699±0.791	20.836±5.729	26.730±7.243	0.255±0.135
MICKNN	13.675±0.064	5.743±0.036	21.528±0.308	16.069±0.108	17.791±0.114	55.954±0.745	16.552±4.533	21.558±5.876	1.699±0.415
SAE	13.432±0.062	5.690±0.033	20.887±0.305	15.369±0.109	16.950±0.115	54.137±0.739	14.939±4.112	19.211±5.286	1.609±0.334
MIDA	13.443±0.065	5.808±0.037	20.106±0.295	15.383±0.109	16.964±0.115	54.129±0.737	14.937±4.114	19.203±5.285	1.613±0.335
BILSTAE	12.335±0.062	5.190±0.028	18.293±0.293	12.516±0.091	14.108±0.098	42.954±0.564	12.103±3.555	16.020±4.657	0.967±0.140
BILSTAE_STAT	11.465±0.061	4.910±0.027	15.785±0.275	10.636±0.078	12.211±0.086	33.824±0.446	10.440±3.074	14.235±4.115	0.721±0.120
Time Causal Methods									
1MEAN	13.472±0.064	5.809±0.037	20.048±0.292	15.726±0.112	17.305±0.117	55.077±0.765	15.001±4.078	19.240±5.300	1.624±0.343
1MEDI	13.472±0.064	5.809±0.037	20.048±0.292	15.726±0.112	17.305±0.117	55.077±0.765	15.001±4.078	19.240±5.300	1.624±0.343
RMEAN	11.661±0.063	5.333±0.033	15.300±0.272	11.053±0.081	12.832±0.090	30.966±0.348	10.920±3.249	15.006±4.333	0.391±0.065
RMEDI	11.796±0.064	5.434±0.035	15.202±0.270	11.070±0.081	12.925±0.092	30.913±0.351	10.968±3.270	15.222±4.410	0.356±0.061
MICET	15.686±0.066	6.043±0.038	21.218±0.244	8.319±0.070	14.868±0.100	27.143±0.438	21.019±5.753	26.983±7.277	0.244±0.121
LOCF	11.861±0.069	5.543±0.036	15.688±0.271	11.038±0.086	13.036±0.098	29.180±0.351	10.775±3.436	15.345±4.639	0.211±0.045
LINEXT	13.472±0.064	5.809±0.037	20.048±0.292	15.726±0.112	17.305±0.117	55.077±0.765	15.001±4.078	19.240±5.300	1.624±0.343
QUAEXT	13.268±0.063	5.681±0.034	19.680±0.288	15.063±0.105	16.675±0.111	53.182±0.714	14.923±4.080	19.401±5.320	1.615±0.338
LSTAE	12.421±0.063	5.259±0.028	18.134±0.294	12.658±0.090	14.287±0.097	42.365±0.548	12.326±3.503	16.339±4.596	1.045±0.212
LSTAE_STAT	11.975±0.062	5.055±0.027	16.924±0.284	11.766±0.086	13.407±0.094	37.647±0.513	11.484±3.356	15.395±4.420	0.866±0.182
HIRID									
Non Time Causal Methods									
PMEAN	13.233±0.098	12.448±0.159	12.710±0.189	33.093±0.436	35.728±0.450	61.710±6.942	24.714±14.288	44.965±33.146	1.177±0.593
PMEDI	12.123±0.092	11.573±0.149	10.915±0.182	20.679±0.198	23.983±0.230	43.103±2.542	22.212±11.563	46.190±34.298	1.066±0.480
LINPOL	6.382±0.077	4.840±0.058	7.098±0.159	11.340±0.177	15.467±0.223	12.856±0.946	10.291±8.143	28.387±25.349	0.080±0.054
NEAPOL	6.465±0.076	4.667±0.054	7.320±0.162	13.679±0.217	18.485±0.264	20.829±6.613	11.423±8.779	31.950±28.175	0.068±0.044
QUAPOL	8.055±0.092	6.207±0.080	8.758±0.161	20.153±0.348	26.819±0.466	19.845±1.093	26.105±25.054	95.367±99.426	0.574±0.625
MICTRE	14.016±0.102	13.520±0.176	13.446±0.211	27.822±0.332	36.942±0.492	61.424±5.519	25.043±13.246	46.272±32.780	0.752±0.219
MICKNN	14.016±0.102	13.520±0.176	13.446±0.211	27.822±0.332	36.942±0.492	61.424±5.519	25.043±13.246	46.272±32.780	0.752±0.219
SAE	13.226±0.098	12.442±0.159	12.697±0.189	32.972±0.434	35.611±0.448	61.616±6.958	24.685±14.255	44.969±33.153	1.175±0.591
MIDA	12.335±0.062	5.190±0.028	18.293±0.293	12.516±0.091	14.108±0.098	42.954±0.564	12.103±3.555	16.020±4.657	0.967±0.140
BILSTAE	9.372±0.081	8.016±0.106	9.523±0.169	20.526±0.265	23.588±0.290	37.102±5.767	18.721±12.468	39.888±32.937	0.697±0.462
BILSTAE_STAT	7.592±0.072	6.201±0.078	7.843±0.157	14.911±0.179	17.846±0.209	25.894±5.111	15.668±10.633	35.724±29.876	0.531±0.315
Time Causal Methods									
1MEAN	12.113±0.091	11.689±0.151	10.843±0.183	19.890±0.172	23.160±0.208	42.742±1.968	22.632±11.952	46.622±35.071	1.086±0.498
1MEDI	12.034±0.095	11.823±0.157	10.528±0.176	19.620±0.172	22.966±0.208	36.301±1.970	22.818±12.125	47.045±35.165	1.104±0.500
RMEAN	8.093±0.068	7.239±0.082	7.212±0.144	13.883±0.137	17.201±0.172	18.597±0.390	15.497±9.329	31.560±23.928	0.298±0.210
RMEDI	8.496±0.074	7.776±0.096	7.488±0.146	13.784±0.142	17.844±0.187	18.200±0.392	15.577±9.561	33.968±26.520	0.337±0.267
MICET	16.463±0.110	16.330±0.205	13.875±0.193	9.771±0.136	20.772±0.264	15.270±1.625	52.693±34.738	28.285±13.192	0.420±0.388
LOCF	6.465±0.076	4.667±0.054	7.320±0.162	13.679±0.217	18.485±0.264	20.829±6.613	11.423±8.779	31.950±28.175	0.068±0.044
LINEXT	7.658±0.081	5.805±0.064	8.048±0.151	15.862±0.216	21.084±0.278	16.564±0.408	17.329±13.818	49.135±44.873	0.233±0.218
QUAEXT	9.944±0.089	8.196±0.092	10.170±0.159	26.668±0.410	34.669±0.517	26.317±0.470	29.971±25.060	86.902±81.484	0.648±0.590
LSTAE	8.915±0.078	7.475±0.095	9.401±0.172	18.536±0.227	21.943±0.260	33.017±5.314	17.613±11.774	38.550±32.016	0.605±0.397
LSTAE_STAT	8.650±0.075	7.194±0.087	9.129±0.169	17.463±0.196	20.651±0.227	29.522±3.595	17.524±11.084	37.263±30.014	0.607±0.341
ICDEP									
Non Time Causal Methods									
PMEAN	14.960±0.082	6.865±0.055	18.345±0.260	13.003±0.087	14.665±0.094	47.330±0.404	12.385±3.290	16.684±4.475	1.510±0.380
PMEDI	13.730±0.065	5.845±0.029	16.698±0.249	12.713±0.086	14.447±0.094	45.217±0.381	12.271±3.251	16.845±4.475	1.495±0.385
LINPOL	13.953±0.096	6.826±0.061	15.899±0.262	10.687±0.085	12.563±0.097	31.518±0.245	10.745±2.948	15.832±4.456	0.559±0.157
NEAPOL	14.253±0.088	6.676±0.057	17.184±0.260	11.722±0.085	13.738±0.097	37.191±0.322	11.463±3.055	16.348±4.468	0.968±0.247
QUAPOL	15.838±0.089	7.229±0.055	18.921±0.267	18.543±0.172	20.627±0.181	55.173±0.439	16.656±4.537	24.933±6.829	0.729±0.192
MICTRE	16.780±0.068	5.686±0.025	22.736±0.242	18.293±0.116	21.369±0.132	58.851±0.604	17.589±4.771	23.438±6.421	0.253±0.130
MICKNN	14.638±0.071	5.966±0.036	20.253±0.287	13.429±0.090	15.259±0.099	47.270±0.478	13.386±3.942	18.287±5.224	1.425±0.382
SAE	14.417±0.064	5.966±0.028	20.552±0.277	13.004±0.087	14.672±0.095	47.269±0.405	12.387±3.293	16.693±4.480	1.505±0.379
MIDA	15.184±0.085	7.032±0.057	18.769±0.266	13.000±0.087	14.660±0.094	47.316±0.404	12.385±3.290	16.684±4.475	1.510±0.380
BILSTAE	14.253±0.067	5.902±0.029	19.765±0.278	11.733±0.079	13.377±0.088	39.703±0.321	11.396±3.049	15.600±4.248	1.159±0.317
BILSTAE_STAT	13.764±0.067	5.652±0.029	19.110±0.276	10.982±0.075	12.634±0.085	35.778±0.276	10.763±2.865	14.957±4.072	1.041±0.271
Time Causal Methods									
1MEAN	15.448±0.079	6.879±0.051	18.891±0.263	15.261±0.098	16.725±0.103	55.469±0.484	14.360±4.010	18.188±5.064	1.684±0.420
1MEDI	14.478±0.071	6.084±0.036	17.264±0.245	14.712±0.095	16.218±0.100	53.118±0.469	14.044±3.923	18.019±4.977	1.637±0.387
RMEAN	14.693±0.086	6.787±0.055	15.889±0.253	12.967±0.095	14.677±0.104	38.528±0.270	12.782±3.585	17.527±5.024	0.670±0.161
RMEDI	14.518±0.088	6.773±0.056	15.870±0.253	12.784±0.096	14.564±0.104	37.995±0.276	12.632±3.503	17.512±4.979	0.637±0.152
MICET	16.689±0.068	5.689±0.025	22.860±0.243	9.639±0.078	15.483±0.120	28.815±0.356	18.173±5.003	25.113±7.573	0.307±0.115
LOCF	13.879±0.091	6.677±0.057	16.051±0.260	12.199±0.096	14.072±0.105	36.146±0.283	12.186±3.352	17.330±4.890	0.550±0.135
LINEXT	15.061±0.092	6.941±0.056	17.120±0.260	15.212±0.126	17.131±0.134	44.840±0.323	14.631±4.051	20.865±5.948	0.546±0.136
QUAEXT	15.838±0.089	7.229±0.055	18.921±0.267	18.543±0.172	20.627±0.181	55.173±0.439	16.656±4.537	24.933±6.829	0.729±0.192
LSTAE	14.164±0.067	5.846±0.029	19.221±0.267	11.760±0.079	13.422±0.088	39.421±0.320	11.382±3.016	15.600±4.203	1.154±0.303
LSTAE_STAT	13.652±0.065	5.563±0.027	18.561±0.262	11.400±0.077	13.058±0.087	36.932±0.293	11.064±2.938	15.253±4.119	0.993±0.258

Table A.7. Performance metrics for imputation methods with MAR amplification The performance of 20 imputation methods is shown for the no amplification. We display the local autocorrelation error ($ACorE_{loc}$), Wasserstein distance thereof ($ACorWSD_{loc}$), cross correlation divergence ($CCorE_{loc}$), normalized mean absolute error ($NMAE_{loc}$), normalized root-mean squared error ($NRMSE_{loc}$), and the local Wasserstein distance ($NWSD_{loc}$). NMAE, NRMSE, and WSD were also derived globally ($NMAE_{glob}$, $NRMSE_{glob}$). We report all errors in % with mean±95%CI across time series (14,286, 11,286, and 17,811 for MIMIC, HIRID, ICDEP), 5 features, and 3 validation folds.

Name	ACorE$_{loc}$	ACorWSD$_{loc}$	CCorE$_{loc}$	NMAE$_{loc}$	NRMSE$_{loc}$	NWSD$_{loc}$	NMAE$_{glob}$	NRMSE$_{glob}$	NWSD$_{glob}$
				MIMIC					
Non Time Causal Methods									
PMEAN	11.546±0.059	4.807±0.028	18.882±0.177	3.970±0.052	6.363±0.069	13.532±0.278	4.073±1.227	9.914±2.770	1.345±0.359
PMEDI	11.513±0.058	4.797±0.028	18.733±0.176	3.939±0.051	6.341±0.067	13.381±0.272	4.049±1.218	9.971±2.786	1.339±0.358
LINPOL	9.117±0.057	4.143±0.024	15.381±0.184	2.628±0.040	4.331±0.054	7.203±0.172	2.475±0.878	6.762±2.165	0.191±0.047
NEAPOL	9.418±0.056	4.038±0.022	16.369±0.182	3.034±0.044	5.002±0.061	8.675±0.208	2.848±0.982	7.777±2.429	0.213±0.055
QUAPOL	10.075±0.059	4.449±0.025	16.894±0.181	3.113±0.045	5.179±0.063	8.300±0.181	3.083±1.090	8.411±2.712	0.163±0.036 hoch
MICTRE	13.799±0.063	5.456±0.034	23.684±0.182	5.721±0.073	9.423±0.099	18.313±0.481	5.737±1.739	13.968±3.895	0.020±0.005
MICKNN	11.868±0.059	4.835±0.028	19.066±0.188	4.112±0.053	6.685±0.071	13.246±0.277	4.158±1.230	10.232±2.806	0.793±0.184
SAE	11.547±0.059	4.808±0.028	18.884±0.177	3.971±0.052	6.364±0.069	13.531±0.278	4.073±1.227	9.914±2.770	1.345±0.359
MIDA	11.556±0.058	4.805±0.028	19.045±0.181	3.973±0.052	6.367±0.069	13.555±0.279	4.074±1.227	9.916±2.771	1.341±0.358
BILSTAE	8.969±0.052	3.895±0.021	14.883±0.174	2.684±0.038	4.379±0.051	8.309±0.174	2.639±0.847	6.874±2.029	0.513±0.134
BILSTAE_STAT	9.379±0.053	4.032±0.022	15.749±0.180	2.839±0.039	4.618±0.053	9.011±0.194	2.799±0.893	7.190±2.110	0.526±0.122
Time Causal Methods									
1MEAN	11.580±0.059	4.816±0.028	18.991±0.177	4.038±0.053	6.447±0.070	13.637±0.283	4.166±1.252	10.074±2.820	1.375±0.373
1MEDI	11.515±0.058	4.799±0.028	18.821±0.176	3.980±0.052	6.382±0.069	12.962±0.255	4.131±1.250	10.082±2.840	1.359±0.365
RMEAN	9.436±0.051	4.173±0.023	15.756±0.173	2.948±0.041	4.833±0.056	8.189±0.171	2.914±0.935	7.557±2.215	0.294±0.072
RMEDI	9.542±0.052	4.231±0.024	15.890±0.173	2.955±0.042	4.869±0.057	8.166±0.172	2.917±0.938	7.671±2.251	0.259±0.063
MICET	13.783±0.063	5.447±0.034	23.775±0.184	5.827±0.045	12.214±0.082	16.524±0.253	5.784±1.739	14.116±3.915	0.077±0.022
LOCF	9.321±0.056	4.131±0.023	16.072±0.179	2.948±0.044	4.877±0.060	7.866±0.173	2.839±0.988	7.723±2.463	0.109±0.028
LINEXT	10.963±0.060	4.616±0.025	18.989±0.179	4.119±0.059	6.878±0.084	10.647±0.193	4.085±1.465	11.126±3.735	0.298±0.073
QUAEXT	12.721±0.060	5.317±0.030	23.088±0.180	6.341±0.094	10.902±0.141	15.706±0.239	6.724±2.464	19.143±6.575	0.989±0.236
LSTAE	10.073±0.055	4.254±0.023	16.939±0.182	3.167±0.043	5.130±0.057	10.366±0.234	3.137±0.974	7.967±2.282	0.734±0.231
LSTAE_STAT	10.019±0.055	4.248±0.024	16.613±0.182	3.203±0.044	5.187±0.059	10.381±0.228	3.229±1.039	8.146±2.434	0.845±0.325
				HIRID					
Non Time Causal Methods									
PMEAN	8.492±0.090	7.833±0.122	10.020±0.112	12.319±0.259	17.914±0.336	15.189±6.431	8.197±8.696	24.982±24.764	0.815±0.697
PMEDI	7.683±0.080	7.169±0.107	8.523±0.104	6.489±0.124	10.680±0.186	7.019±1.619	6.751±7.001	25.687±25.511	0.692±0.559
LINPOL	4.362±0.069	3.187±0.050	5.933±0.113	4.633±0.113	8.145±0.170	6.466±6.396	4.060±4.475	17.440±18.531	0.055±0.051
NEAPOL	4.434±0.068	3.101±0.047	6.056±0.111	5.349±0.130	9.392±0.192	7.386±6.403	4.416±4.831	19.592±20.666	0.038±0.037
QUAPOL	5.334±0.082	3.921±0.066	16.852±0.111	7.148±0.167	12.413±0.269	8.154±6.402	7.774±8.687	37.337±40.930	0.246±0.275
MICTRE	11.559±0.109	11.543±0.166	13.089±0.123	16.966±0.391	41.793±0.986	17.561±4.427	11.060±11.619	35.541±35.209	0.015±0.013
MICKNN	8.441±0.093	7.987±0.131	9.408±0.112	10.888±0.244	21.225±0.452	19.283±18.679	8.140±8.669	24.912±24.986	0.286±0.244
SAE	8.503±0.090	7.842±0.122	10.039±0.112	12.379±0.260	17.991±0.337	15.260±6.460	8.216±8.716	24.981±24.765	0.817±0.699
MIDA	8.469±0.089	7.790±0.121	10.093±0.116	12.037±0.253	17.549±0.329	14.895±6.419	8.117±8.612	24.990±24.773	0.807±0.689
BILSTAE	5.249±0.068	4.330±0.070	7.255±0.113	5.765±0.119	9.304±0.173	7.150±4.277	5.624±6.107	21.265±22.003	0.353±0.323
BILSTAE_STAT	5.156±0.069	4.319±0.073	6.788±0.107	5.803±0.119	9.345±0.174	7.917±6.178	5.604±6.055	20.354±21.128	0.380±0.355
Time Causal Methods									
1MEAN	7.663±0.080	7.260±0.110	8.411±0.102	6.311±0.116	10.451±0.179	6.365±0.318	6.977±7.234	26.167±26.064	0.715±0.578
1MEDI	7.578±0.081	7.331±0.114	8.197±0.103	6.430±0.117	10.596±0.181	6.140±0.277	7.074±7.358	26.325±26.198	0.727±0.587
RMEAN	5.145±0.060	4.515±0.067	6.440±0.100	5.189±0.102	8.716±0.157	4.100±0.254	5.188±5.483	18.230±18.310	0.242±0.229
RMEDI	5.452±0.067	4.883±0.079	6.825±0.103	5.376±0.108	9.330±0.171	4.186±0.255	5.313±5.640	19.958±20.292	0.293±0.293
MICET	10.620±0.098	10.318±0.148	11.559±0.119	8.592±0.142	18.842±0.297	7.131±0.825	8.299±8.475	28.388±27.007	0.334±0.352
LOCF	4.331±0.065	3.115±0.046	5.488±0.102	4.322±0.092	7.842±0.151	2.905±0.252	4.136±4.486	19.000±19.909	0.017±0.016
LINEXT	5.207±0.072	3.829±0.055	6.506±0.104	5.881±0.122	10.527±0.204	3.822±0.256	6.467±7.064	29.343±31.013	0.193±0.200
QUAEXT	7.118±0.083	5.858±0.085	8.505±0.110	8.970±0.191	16.342±0.331	5.796±0.272	11.012±12.017	51.700±54.785	0.520±0.526
LSTAE	5.740±0.072	4.795±0.078	7.986±0.121	6.882±0.154	10.784±0.212	10.289±8.724	5.989±6.565	21.241±21.763	0.422±0.384
LSTAE_STAT	5.449±0.070	4.378±0.067	7.871±0.120	6.416±0.128	9.982±0.182	7.049±3.950	6.104±6.631	21.283±22.028	0.375±0.341
				ICDEP					
Non Time Causal Methods									
PMEAN	9.624±0.089	3.741±0.036	13.005±0.164	3.525±0.067	4.888±0.081	10.591±0.216	2.222±1.064	6.339±2.280	0.665±0.350
PMEDI	9.456±0.087	3.643±0.033	12.657±0.162	3.493±0.066	4.868±0.081	10.331±0.207	2.193±1.055	6.366±2.303	0.656±0.347
LINPOL	8.335±0.089	3.542±0.038	11.483±0.172	2.973±0.064	4.131±0.077	7.849±0.162	1.791±0.856	5.747±2.097	0.207±0.108
NEAPOL	8.748±0.088	3.510±0.036	12.203±0.167	3.275±0.066	4.587±0.082	8.929±0.188	2.003±0.960	6.186±2.274	0.278±0.137
QUAPOL	5.334±0.082	3.921±0.066	6.852±0.111	7.148±0.167	12.413±0.269	8.154±6.402	7.774±8.687	37.337±40.930	0.246±0.275
MICTRE	9.456±0.089	3.477±0.031	11.827±0.160	3.430±0.066	4.805±0.081	9.969±0.217	2.227±1.004	6.543±2.250	0.319±0.149
MICKNN	9.456±0.089	3.477±0.031	11.827±0.160	3.430±0.066	4.805±0.081	9.969±0.217	2.227±1.004	6.543±2.250	0.319±0.149
SAE	9.595±0.088	3.714±0.035	12.937±0.163	3.528±0.067	4.892±0.081	10.605±0.216	2.223±1.065	6.340±2.282	0.666±0.351
MIDA	9.456±0.089	3.477±0.031	11.827±0.160	3.430±0.066	4.805±0.081	9.969±0.217	2.227±1.004	6.543±2.250	0.319±0.149
BILSTAE	8.054±0.079	3.253±0.030	10.547±0.150	2.838±0.057	3.944±0.070	7.891±0.159	1.757±0.824	5.307±1.913	0.374±0.194
BILSTAE_STAT	8.066±0.079	3.241±0.030	10.513±0.148	2.854±0.058	3.971±0.070	7.960±0.160	1.763±0.834	5.318±1.927	0.385±0.204
Time Causal Methods									
1MEAN	10.127±0.091	3.857±0.036	13.481±0.164	3.986±0.074	5.449±0.088	12.354±0.261	2.561±1.196	6.969±2.447	0.746±0.392
1MEDI	9.890±0.090	3.766±0.034	13.163±0.162	3.844±0.072	5.272±0.086	11.712±0.246	2.484±1.145	6.860±2.406	0.715±0.371
RMEAN	9.018±0.085	3.622±0.035	12.231±0.166	3.504±0.071	4.827±0.085	9.318±0.188	2.215±1.011	6.585±2.359	0.238±0.114
RMEDI	8.968±0.086	3.615±0.035	12.243±0.167	3.496±0.071	4.822±0.085	9.315±0.189	2.185±1.010	6.593±2.349	0.215±0.102
MICET	11.169±0.097	3.784±0.033	17.120±0.187	4.998±0.058	9.498±0.099	13.343±0.215	3.271±1.526	9.391±3.367	0.067±0.031
LOCF	8.539±0.087	3.536±0.036	11.938±0.170	3.428±0.071	4.737±0.086	9.099±0.190	2.094±0.989	6.509±2.342	0.160±0.075
LINEXT	9.580±0.094	3.777±0.037	13.128±0.174	4.300±0.089	5.961±0.109	11.314±0.224	2.683±1.283	8.220±3.054	0.209±0.110
QUAEXT	10.429±0.095	4.015±0.037	14.951±0.180	5.295±0.113	7.478±0.143	13.930±0.280	3.437±1.681	10.968±4.212	0.409±0.231
LSTAE	8.679±0.082	3.413±0.031	11.723±0.159	3.121±0.061	4.339±0.075	8.884±0.181	1.955±0.916	5.780±2.065	0.452±0.234
LSTAE_STAT	8.585±0.082	3.368±0.030	11.266±0.153	3.082±0.061	4.283±0.074	8.807±0.180	1.924±0.914	5.671±2.037	0.455±0.246

Table A.8. Performance metrics for imputation methods with MNAR amplification The performance of 20 imputation methods is shown for the MNAR amplification. We display the local autocorrelation error (ACorE$_{loc}$), Wasserstein distance thereof (ACorWSD$_{loc}$), cross correlation divergence (CCorE$_{loc}$), normalized mean absolute error (NMAE$_{loc}$), normalized root-mean squared error (NRMSE$_{loc}$), and the local Wasserstein distance (NWSD$_{loc}$). NMAE, NRMSE, and WSD were also derived globally (NMAE$_{glob}$, NRMSE$_{glob}$). We report all errors in % with mean±95%CI across time series (14,286, 11,286, and 17,811 for MIMIC, HIRID, ICDEP), 5 features, and 3 validation folds.

	MCAR without spatio-temporal missingness patterns			MC induced spatio-temporal missingness patterns		
Method	$CCorD_{loc}$	$NRMSE_{loc}$	$NWSD_{glob}$	$CCorD_{loc}$	$NRMSE_{loc}$	$NWSD_{glob}$
MIMIC						
LINPOL	14.024±0.172	3.136±0.060	0.140±0.038	6.581±0.164	10.578±0.103	0.090±0.016
BILSTAE	10.143±0.131	1.947±0.046	0.248±0.043	6.581±0.159	10.840±0.097	0.234±0.041
SAITS (ref)	13.954±0.145	5.394±0.053	0.489±0.096	10.002±0.196	6.399±0.060	0.424±0.084
BRITS (ref)	9.067±0.125	3.003±0.033	0.169±0.030	6.220±0.156	3.926±0.041	0.172±0.029
CTA (ref)	13.875±0.178	5.205±0.065	0.623±0.157	9.916±0.245	6.369±0.077	0.532±0.135
STGAE (ref)	9.125±0.159	16.225±0.135	0.417±0.209	13.730±0.256	24.327±0.202	0.554±0.160
HIRID						
LINPOL	4.524±0.095	8.337±0.183	0.043±0.040	3.384±0.090	17.824±0.296	0.026±0.024
BILSTAE	3.729±0.074	6.694±0.138	0.152±0.138	3.348±0.091	18.685±0.364	0.337±0.385
SAITS (ref)	8.887±0.100	31.965±0.600	0.427±0.407	7.510±0.115	24.537±0.441	0.388±0.387
BRITS (ref)	3.080±0.069	6.577±0.108	0.101±0.100	3.116±0.082	7.095±0.126	0.102±0.102
CTA (ref)	7.101±0.109	18.595±0.382	0.424±0.468	6.573±0.130	18.280±0.386	0.392±0.455
STGAE (ref)	7.861±0.103	13.951±0.142	0.390±0.135	5.892±0.100	35.975±0.471	0.452±0.439
ICDEP						
LINPOL	19.836±0.176	10.462±0.103	0.283±0.138	7.555±0.156	12.539±0.128	0.184±0.069
BILSTAE	9.389±0.135	4.244±0.059	0.239±0.148	8.166±0.156	13.040±0.119	0.281±0.132
SAITS (ref)	14.941±0.145	6.446±0.064	0.449±0.200	9.718±0.169	8.201±0.077	0.328±0.157
BRITS (ref)	10.583±0.123	4.550±0.052	0.268±0.138	7.563±0.151	6.242±0.069	0.249±0.118
CTA (ref)	19.276±0.205	18.285±0.756	1.622±1.245	12.646±0.232	22.069±0.798	1.371±1.429
STGAE (ref)	9.521±0.159	16.225±0.135	0.417±0.209	16.529±0.378	23.033±0.213	0.496±0.561

Table A.9. Performance metrics for reference baseline models (ref) on data with and without spatio-temporal patterns. Either data are following the MCAR mechanisms without spatio-temporal patterns, or data are induced with spatio-temporal pattern by applying the Markov Chain (MC) simulation model. We report all errors in % with mean±95%CI across time series (14,286, 11,286, and 17,811 for MIMIC, HIRID, ICDEP), 5 features, and 3 validation folds.

		MAE$_{loc}$			RMSE$_{loc}$		
feature	method	MIMIC	HIRID	ICDEP	MIMIC	HIRID	ICDEP
bp_dia	LOCF	7.93±0.21	4.03±0.12	9.85±0.23	11.52±0.37	7.77±0.16	13.81±0.29
	BILSTAE	7.15±0.32	5.58±0.82	8.75±0.38	9.91±0.52	8.23±0.6	11.74±0.59
	LINPOL	6.92±0.28	3.12±0.11	8.53±0.2	10.18±0.48	6.64±0.54	12.11±0.33
	LSTAE	7.93±0.16	5.29±0.19	9.46±0.18	10.85±0.29	8.07±0.19	12.5±0.21
bp_sys	LOCF	12.09±0.11	7.24±0.2	14.44±0.31	16.63±0.24	11.41±0.24	20.14±0.36
	BILSTAE	11.15±0.05	10.96±1.55	12.71±0.81	14.85±0.15	14.78±1.42	17.37±0.83
	LINPOL	10.49±0.16	5.35±0.2	12.38±0.3	14.75±0.35	8.99±0.44	17.71±0.34
	LSTAE	12.53±0.19	10.84±0.52	13.58±0.42	16.56±0.41	14.81±0.35	18.27±0.42
hr	LOCF	6.86±0.12	2.44±0.2	10.57±0.37	10.25±0.17	5.48±1.19	13.76±0.43
	BILSTAE	7.44±0.19	6.26±0.7	8.92±0.48	10.22±0.14	9.46±0.92	11.95±0.9
	LINPOL	5.9±0.1	1.82±0.19	9.46±0.29	9.18±0.17	4.64±1.23	12.9±0.55
	LSTAE	9.07±0.1	6.34±0.23	9.98±0.29	12.25±0.15	9.71±0.42	13.2±0.51
rr	LOCF	3.6±0.05	9.31±1.03	2.22±0.05	5.01±0.04	30.51±3.9	3.55±0.55
	BILSTAE	3.12±0.03	13.25±1.15	1.83±0.03	4.19±0.06	34.72±2.56	2.98±0.37
	LINPOL	3.23±0.03	9.27±1.01	1.9±0.07	4.49±0.02	28.45±3.67	3.21±0.43
	LSTAE	3.43±0.11	12.62±1.96	1.86±0.05	4.57±0.14	32.82±5.51	3.01±0.4
spo2	LOCF	1.68±0.06	0.83±0.01	0.86±0.02	2.97±0.28	1.69±0.16	1.38±0.04
	BILSTAE	1.55±0.02	1.38±0.09	0.84±0.03	2.6±0.13	2.28±0.24	1.29±0.01
	LINPOL	1.53±0.06	0.66±0.01	0.86±0.04	2.7±0.22	1.48±0.13	1.34±0.04
	LSTAE	1.72±0.05	1.38±0.07	0.96±0.02	2.74±0.12	2.25±0.19	1.4±0.03

Table A.10. Mean absolute error (MAE) and root-mean squared error (RMSE) per feature. For each feature, the local MAE and RMSE is displayed as mean±standard-deviation grouped by the imputation method and per dataset (MIMIC, HIRID, ICDEP). The reported summary statistics are calculated across all patient time series (14,286, 11,286, and 17,811 for MIMIC, HIRID, ICDEP) and across all 3 cross-validation folds.

Amplification	Method	CCorE$_{loc}$	NRMSE$_{loc}$	NWSD$_{glob}$
		MIMIC		
MNAR reverse	LINPOL	15.381±0.184	4.331±0.054	0.191±0.047
	BILSTAE	21.324±0.201	6.357±0.060	0.878±0.172
	BILSTAE_STAT	20.853±0.199	6.083±0.058	0.761±0.138
	LOCF	16.072±0.179	4.877±0.060	0.109±0.028
	LSTAE	22.126±0.203	6.698±0.063	0.890±0.168
	LSTAE_STAT	22.073±0.201	6.721±0.063	0.935±0.149
MNAR low	LINPOL	14.553±0.180	5.119±0.043	0.171±0.029
	BILSTAE	14.906±0.180	5.369±0.042	0.452±0.074
	BILSTAE_STAT	14.734±0.178	5.292±0.041	0.446±0.075
	LOCF	15.251±0.177	5.784±0.047	0.100±0.021
	LSTAE	15.671±0.183	5.758±0.044	0.542±0.093
	LSTAE_STAT	15.460±0.182	5.694±0.044	0.522±0.090
MAR low	LINPOL	12.267±0.212	4.924±0.048	0.199±0.035
	BILSTAE	20.603±0.300	6.268±0.063	0.990±0.169
	BILSTAE_STAT	20.509±0.300	6.195±0.062	0.958±0.174
	LOCF	13.003±0.212	5.538±0.053	0.128±0.026
	LSTAE	21.236±0.300	6.676±0.065	0.923±0.198
	LSTAE_STAT	21.054±0.299	6.614±0.065	0.901±0.194
		HIRID		
MNAR reverse	LINPOL	5.933±0.113	8.145±0.170	0.055±0.051
	BILSTAE	8.834±0.121	11.346±0.202	0.448±0.433
	BILSTAE_STAT	8.352±0.118	11.321±0.203	0.460±0.444
	LOCF	5.488±0.102	7.842±0.151	0.017±0.016
	LSTAE	8.408±0.122	10.602±0.186	0.413±0.377
	LSTAE_STAT	8.223±0.120	10.643±0.190	0.438±0.399
MNAR low	LINPOL	5.305±0.098	8.264±0.133	0.017±0.016
	BILSTAE	7.668±0.108	12.777±0.217	0.553±0.564
	BILSTAE_STAT	7.383±0.105	12.283±0.208	0.552±0.560
	LOCF	5.615±0.105	8.555±0.152	0.053±0.049
	LSTAE	8.759±0.120	13.285±0.222	0.526±0.529
	LSTAE_STAT	7.840±0.110	12.486±0.209	0.547±0.541
MAR low	LINPOL	6.763±0.125	8.620±0.127	0.021±0.012
	BILSTAE	9.543±0.173	11.549±0.205	0.794±0.545
	BILSTAE_STAT	9.043±0.122	10.196±0.205	0.689±0.522
	LOCF	6.898±0.134	8.954±0.156	0.064±0.039
	LSTAE	9.294±0.181	11.465±0.216	0.815±0.531
	LSTAE_STAT	9.047±0.180	11.414±0.216	0.800±0.541
		ICDEP		
MNAR reverse	LINPOL	11.483±0.172	4.131±0.077	0.160±0.075
	BILSTAE	19.611±0.175	8.483±0.096	1.040±0.545
	BILSTAE_STAT	19.375±0.173	8.510±0.095	1.071±0.554
	LOCF	11.938±0.170	4.737±0.086	0.207±0.108
	LSTAE	23.471±0.175	8.553±0.096	1.016±0.504
	LSTAE_STAT	24.535±0.172	8.928±0.098	1.231±0.605
MNAR low	LINPOL	18.391±0.183	7.584±0.070	0.247±0.118
	BILSTAE	17.576±0.160	7.461±0.062	0.601±0.292
	BILSTAE_STAT	17.259±0.159	7.388±0.062	0.584±0.283
	LOCF	18.965±0.174	8.745±0.079	0.324±0.166
	LSTAE	18.130±0.162	7.862±0.064	0.683±0.323
	LSTAE_STAT	17.807±0.160	7.813±0.064	0.661±0.312
MAR low	LINPOL	12.787±0.217	5.655±0.064	0.278±0.067
	BILSTAE	19.327±0.272	6.987±0.068	1.014±0.252
	BILSTAE_STAT	19.300±0.273	6.965±0.068	1.006±0.250
	LOCF	12.256±0.218	6.397±0.070	0.312±0.092
	LSTAE	19.734±0.277	7.251±0.069	1.103±0.270
	LSTAE_STAT	19.918±0.285	7.246±0.069	1.083±0.269

Table A.11. **Performance metrics for MAR and MNAR amplifications using different scaling factors** Three scenarios are constructed: MNAR reverse, MNAR low, MAR low using scaling factors of {3.4, 2.4, 1.4}, {1, 1.5, 2}, and {1, 1.1, 1.2}, respectively. We report all errors in % with mean±95%CI across time series (14,286, 11,286, and 17,811 for MIMIC, HIRID, ICDEP), 5 features, and 3 validation folds.

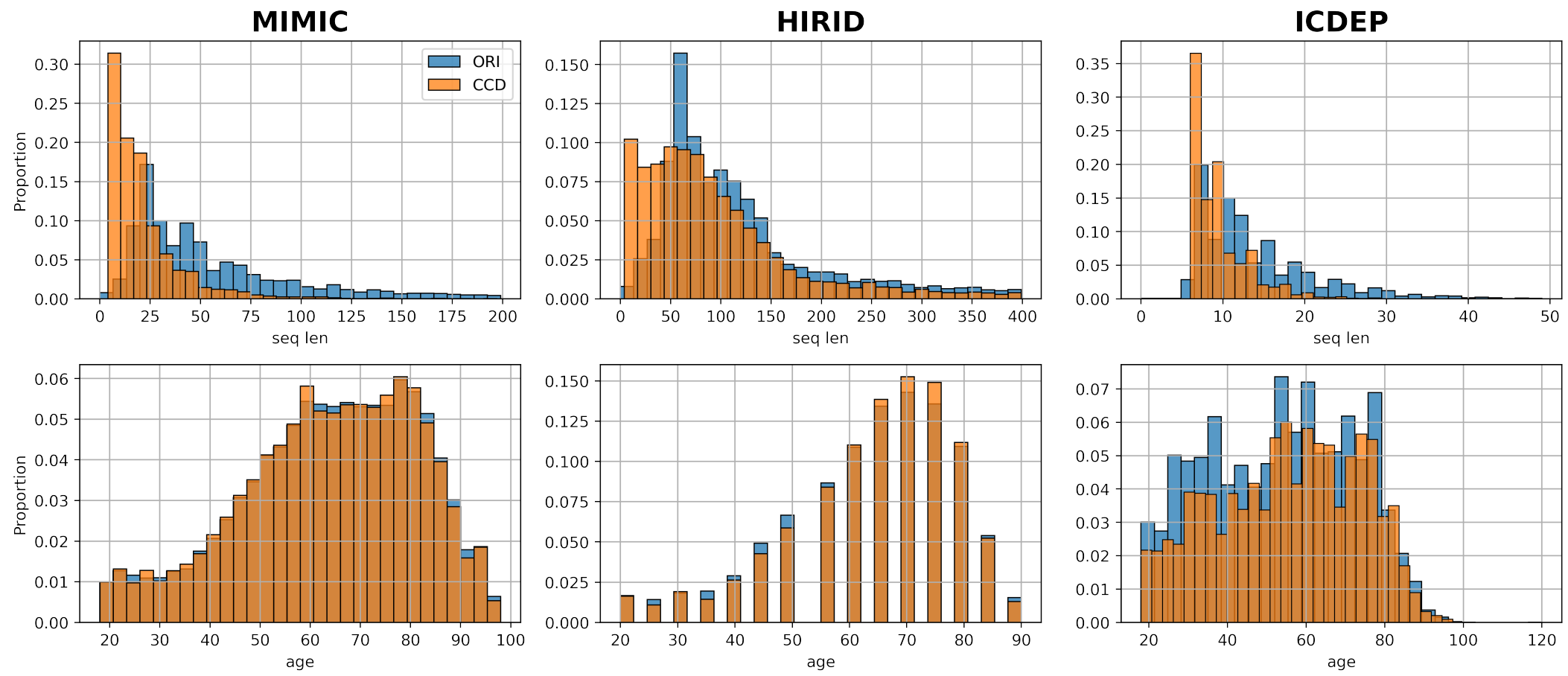

Figure A.1. Visualization of patient characteristics for original - and complete case data. Differences between original (ORI) and complete case data (CDD), preserving spatio-temporal missingness after cleansing, are shown for the sequence length (seq len) and age [in years]. N=14,286, N=11,286, and N=17,811 subjects are included in MIMIC, HIRID, and ICDEP, respectively.

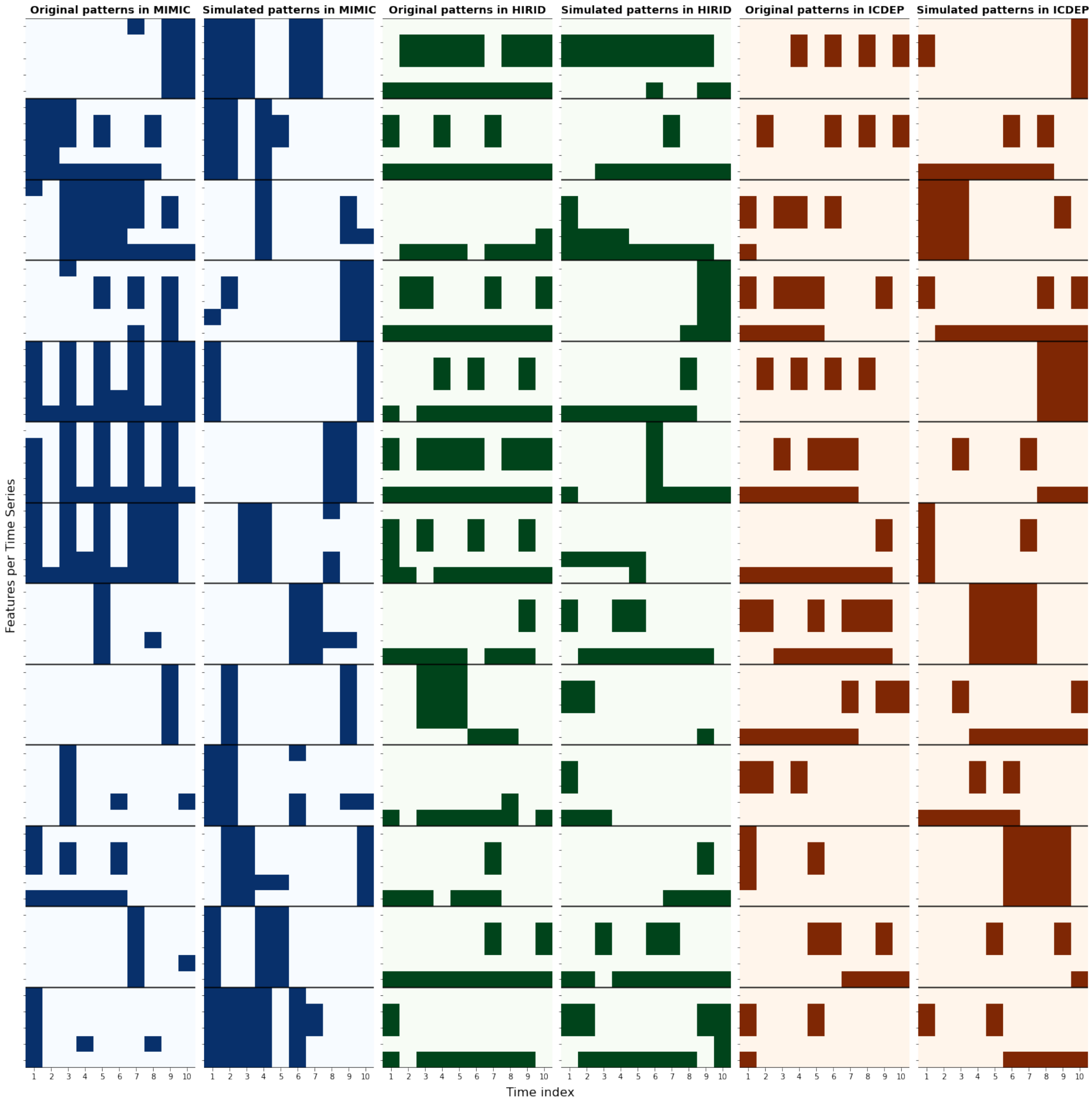

Figure A.2. Original and simulated spatio-temporal missingness patterns per dataset. We present a random selection of 13 observation windows (5 features $\times$ 10 time indexes), bounded by black lines, for original and simulated data per set MIMIC, HIRID, ICDEP. Colorization indicates missing values, feature values are present in white planes. We could find similar patterns of simulated missingness also present in the original data indicating that our simulation model captured the temporal structure thereof.

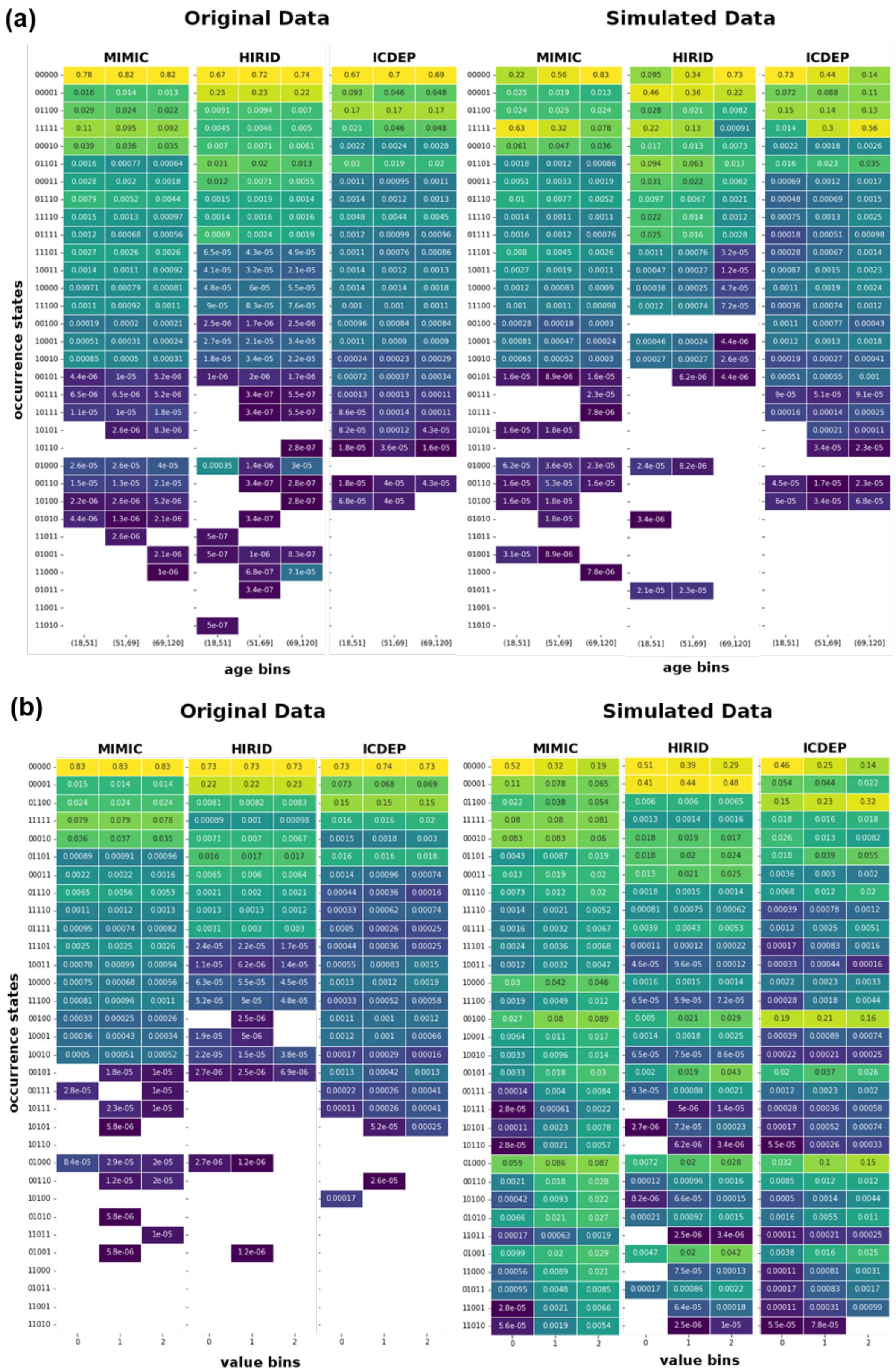

Figure A.3. Occurrence probabilities before and after missing at random (a) or missing not at random (b) amplification. Occurrence states were either conditioned on equally sized age bins (panel a) to amplify the missing at random (MAR) effect, or on ground truth (GT) value bins (panel b) to amplify a missing not at random (MNAR) effect. Value bins per feature are included in Table A.4.

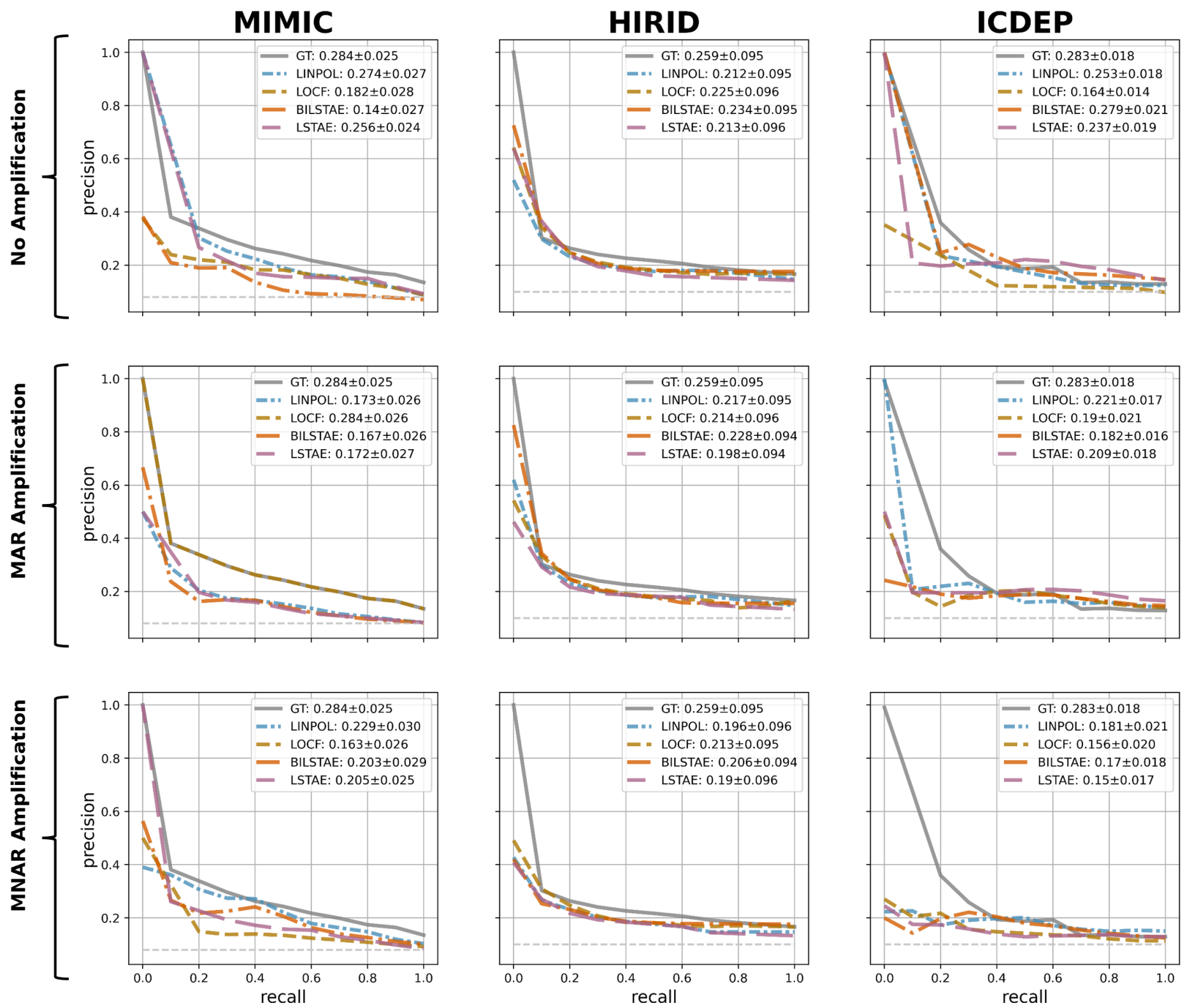

Figure A.4. Area under the precision-recall (AUPRC) curves across amplification scenarios. We display the area under the precision-recall curve (AUPRC) for ground truth (GT) and imputed datasets. Linear interpolation (LINPOL), last observation carried forward (LOCF), LSTM autoencoder (LSTAE), and bidirectional LSTM autoencoder (BILSTM) were applied for imputations. All results are gained from a downstream transformer model classifying in-hospital death (MIMIC, HIRID) or postoperative delirium (ICDEP).

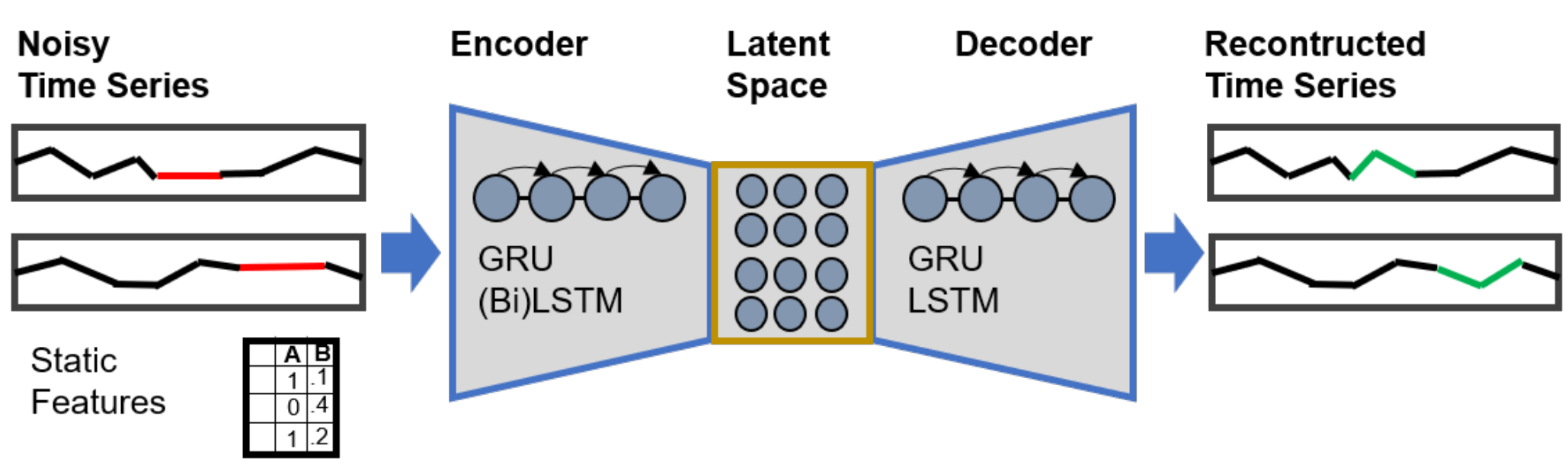

Figure A.5. Spatio-temporal autoencoder architecture. Noisy time series, initially filled with constants, are compressed via DL methods like LSTMs to a latent space and decoded for reconstructing time series, imputing missing longitudinal values. The encoding to the latent space was optionally extended by a linear layer that added static data.

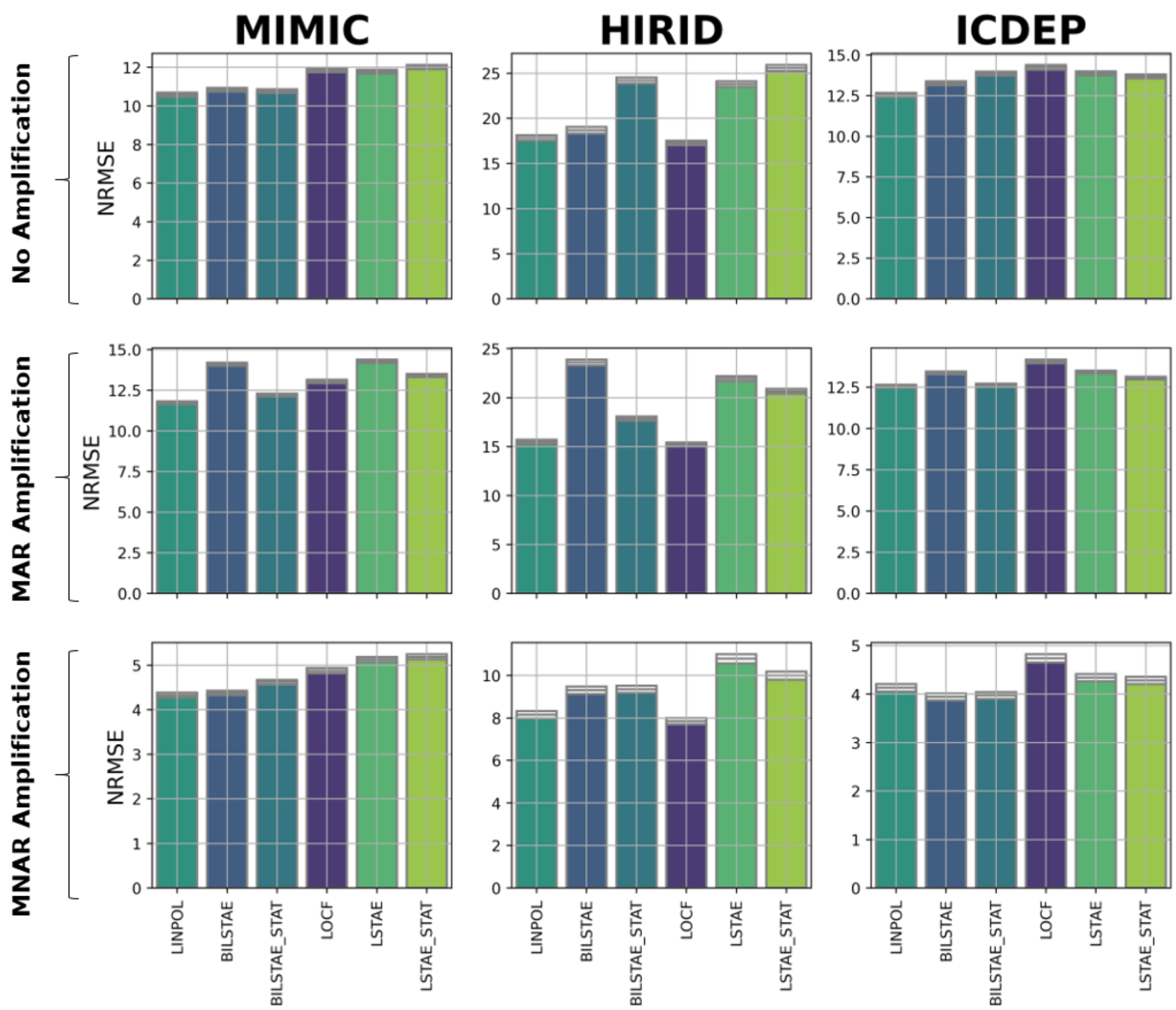

Figure A.6. NRMSE for selected imputation methods across amplification scenarios. We selected linear interpolation (LINPOL), LOCF, bi-directional LSTM (BILSTAE) and unidirectional LSTM (LSTAE) autoencoder, these autoencoders were optionally trained with static features (_STAT). We display the mean of the local normalized root mean squared error (NRMSE) with 95%CI error bars on top calculated across patients and features in %. Training with additional static features was especially beneficial for autoencoders under the missing at random (MAR) amplification across all datasets and for the LSTAE models in HIRID and ICDEP under amplified missing not at random (MNAR) effects.

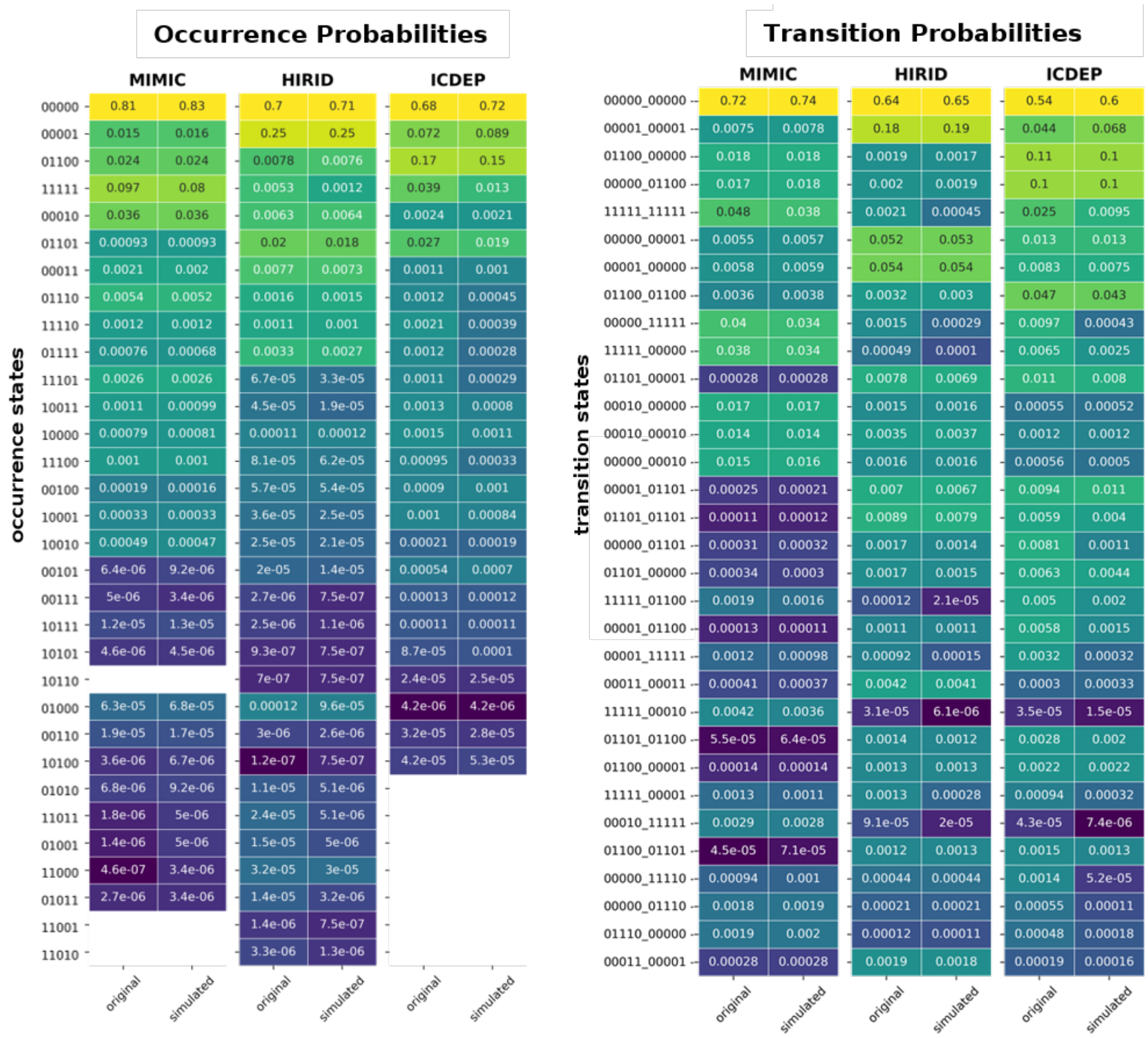

Figure A.7. Occurrence and transition probabilities after data cleaning with outlier removal. For each dataset MIMIC, HIRID, ICDEP, occurrence as well as transition probabilities from one state to the subsequent one are shown. Outliers were set to missing also altering the underlying spatio-temporal missingness pattern we aim to simulate.

Additional Methods A.1

In addition to feature missingness FM, we described missingness in time series data by defining the metrics single missingness (SM) and block missingness (BM). SM calculates the fraction of missing values within one single time series per individual patient n and feature j with

$$\mathrm{SM}_{n,j} = \frac{Q_{n,j}}{l_n} \tag{1}$$

where $Q_{n,j} = \sum_{t=1}^{l_n} r_{j,t}$ calculates the number of missing values per feature time series of length l_n. BM describes the fraction of the largest sequential adjacent number of consecutive missing values from all time series values. In equation 1, the term $Q_{n,j}$ is simply limited to consecutive values only for calculating BM. In Table A.3, we report SM and BM in addition to FM to outline the quality of our simulation model.

For quantifying global errors between imputed data (denoted as $\bar{\mathbf{X}}$) and ground truth data (defined by $\mathbf{Z}$), we calculated the normalized RMSE and the normalized MAE per feature j for the entire patient population, instead of retrieving one error per patient time series locally as

$$\mathrm{NRMSE}_j = \frac{\sqrt{\frac{1}{I}\sum_{i=1}^{I}(z_{j,i} - \bar{x}_{j,i})^2}}{\mu_n(z_j)}, \tag{2}$$

$$\text{NMAE}_j = \frac{\frac{1}{I}\sum_{i=1}^{I} |z_{j,i} - \bar{x}_{j,i}|}{\mu_n(z_j)} \tag{3}$$

where I is the number of all missing feature values across the entire population and $\mu_n(z_j)$ calculates the mean value of all ground truth values for which imputation was applied. Examples of reported scores are presented in Table A.5.

Additional Results A.1

After setting outlier values that did not fall into valid value ranges to missing (alternative preprocessing to the main manuscript), we trained our Makov simulation model on occurrence and transition probabilities from the resulting missingness patterns (see Figure A.7). We noticed the occurrence of more states (29, 32, 25 for MIMIC, HIRID, ICDEP) compared to the preservation of original missingness (28, 23, 24 for MIMIC, HIRID, ICDEP), reported in the main manuscript. The transitions from one total present state (TPS), where all features are observed with $s = [0,0,0,0,0]$, to another TPS were most frequent ($f = 0.72$, 0.64, 0.54 for MIMIC, HIRID, ICDEP). Transitions that included states for the simultaneous presence of bp_sys and bp_dia ($\mathbf{s} = [0,1,1,0,0]$) or the isolated presence of respiratory rate ($\mathbf{s} = [0,0,0,0,1]$) were relatively frequent across datasets, similar to results reported in the main manuscript. When applying imputation methods on simulated missingness patterns following outlier removal, deep learning autoencoder did also not outperform simple baseline models, similar to presented results in the main manuscript. In MIMIC, the local NRMSE$_{loc}$ was at 1.588±0.047 (as mean±standard-deviation) and 1.798±0.053 for LOCF and LINPOL as baseline methods and increased with 1.813±0.027 and 1.963±0.028 for BILSTAE and LSTAE for autencoder models.

Additional Results A.2

We conducted an extensive ablation study as seen in Table A.6. We trained multiple spatio-temporal denoising autoencoder (STAE) architectures iterating over two different loss functions (MSE loss v.s. combined loss joining WSD, that was implemented with a Sinkhorn[1] loss, and MSE), the inclusion of static attributes (in contrast to stand-alone models), or the construction of GT values with known CDD data. In contrast, the main manuscript reports STAE results from training with LOCF pre-imputed time series because the underlying GT values are usually not known. In ICDEP, performance of the BILSTAE trained on CDD was superior to training on pre-imputed values (CCorE$_{loc}$= 7.531±0.146 v.s. 8.166±0.156, NRMSE$_{loc}$= 12.352±0.114 v.s. 13.040±0.119). The contrary effect was observed for the same model in MIMIC (NRMSE$_{loc}$= 10.840±0.097 for pre-imputed v.s. 11.035±0.098 for CDD GT values). We found improved RMSE scores for LSTAE trained with a combined loss function in MIMIC (11.468±0.103 v.s. 11.544±0.102) and in HIRID (NRMSE$_{loc}$= 22.662±0.298 v.s. 21.964±0.278) in comparison of using a single MSE loss function. In ICDEP, the BILSTAE models that were trained on known CCD could improve standard modules by the inclusion of static attributes as well as by training with a combined loss function (NRMSE$_{loc}$= 3.324±0.118 v.s. 12.352±0.114). Due to the superior performance of LSTM modules over GRU modules, we did further training and optimization with LSTMs. We used padding and packing to process sequences of uneven lengths but using raw sequences with (Bi)LSTM autoencoder and batch size 1 seemed to be more promising. Overall, results showed no clear advantage of one ablation parameter over the others, with performances varying a lot across datasets (see Table A.6).

Additional Results A.3

We also compared our models LSTAE and BILSTAE to published and well-known DL imputation models. Due to the configuration of LSTM modules in BRITS, this model was trained with batch size of one and accumulating gradients, similar to the STAE models reported in the main manuscript. STGAE and SAITS were trained on time series that were padded to the longest sequence within one batch. CTA was adopted so that the model could also handle time series of unequal lengths. To inherently learn to impute time series via masking, 10% of the actual observed values inside the training set were masked out. Hereby, no LOCF pre-imputation was required for learning, while only the imputed values contributed to the MSE loss. All reference model architectures were trained with published hyperparameter configurations[2–5].

Additional Results A.4

We assessed the sensitivity of imputation methods under varying missing intensities for simulation scenarios with amplified MAR and MNAR effects. For additionally evaluating MNAR effects in the reverse direction, scaling factors of 3.4, 2.4, 1.4 were used in the MNAR reverse setting. The main manuscript reports experiments using large shifts in missing rates with respect to age-bins for MAR as well as to value-bins for MNAR amplifications. Hence, the scenarios MAR low as well as MNAR low were constructed using smaller scaling factors of 1, 1.5, 2 and 1, 1.1, 1.2, respectively. Table A.11 displays sensitivity results with respect to six imputation methods evaluated with three performance metrics across amplification scenarios and databases. As a result, linear imputation methods were evaluated as robust imputer in contrast to DL autoencoders, supporting the findings of the main manuscript.

The Table A.9 displays results from trained reference models (ref), which were all considered non-time causal since they infer statistics from future time points, in comparison to linear interpolation (LINPOL) and BiLSTM autoencoder (LSTM). In scenario A), models imputed patterns that strictly follow a MCAR mechanism without spatio-temporal structure, as done by previous authors that claim superiority of DL imputers over linear ones. In scenario B), models are applied on more complex and realistically simulated spatio-temporal missingness patterns that we detected in real-world clinical time series. For the oversimplified MCAR scenario, LINPOL yielded lowest globally computed NWSD scores for MIMIC and HIRID. However, more advanced models comprising the reference BRITS and the BILSTAE architecture resulted in lowest CCorD and NRMSE scores across all three datasets. In ICDEP, BILSTAE imputed with best evaluations in comparison to LINPOL as well as all reference models.

Overall, results support findings from the main manuscript, indicating that the simulated missingness patterns from Markov chains pose more complex scenarios for DL imputation methods than simple MCAR inductions lacking in spatio-temporal structure. The improved performance of BRITS through jointly learning imputation as well as classification tasks suggests that downstream labels contain valuable auxiliary information for time series imputation. Similar claims have been made by benchmarking studies before[6,7]. However, treating these two tasks as independent problems, as reported in the main manuscript, has the advantage of freely selecting any downstream task. Also, since other reference models (SAITS, CTA, STGAE) do not have access to the labels, BRITS evaluations must be interpreted carefully. The BILSTAE trained on ICDEP could prevail over all reference models for the MCAR scenario. Here we showed that an autoencoder were successfully trained to model heterogeneous multivariate time series imputations.

Additional Discussion A.1

Figure A.3 quantifies changes in occurrence probabilities before and after MAR (panel a) or MNAR (panel b) amplifications across either age-bins or value-bins, respectively. For the amplification of MAR effects, we changed the proposed sampling procedure so that proportions of probabilities for the total present state (**TPS** = [0,0,0,0,0]) are shifted to the total absent state (**TAS** = [1,1,1,1,1]) to induce missingness, conditioned on a patient's age. As expected, Figure A.4a reflect this behavior (e.g., probabilities of 0.22, 0.56, 0.83 for **TSP** across three age-bins in MIMIC) without major changes in the order of more complex missing states (e.g., probability of 0.024±0.005 for the original and simulated state **s** = [0,1,1,0,0]). Due to shifts in overall availability probabilities, minor changes in seldom states occurred for MAR amplifications.

Figure A.4b shows that after simulating spatio-temporal missingness patterns on CCD, no ad-hoc MNAR was detected since the resampling procedure was not conditioned on feature value levels. After amplification of these effects, seldom states became more frequent in the simulated data. The sampling procedure was adapted so that after simulating original spatio-temporal missingness, feature values were removed according to missingness rates that depend on the GT value level. Hence, overall states where the individual feature was encoded as absent became more likely depending on the underlying true GT value distributions. While the main manuscript includes results from a negative MNAR effect (higher missingness for lower values), ablations with a positive effect direction led to the same benchmarking findings. Overall, results across all amplification scenarios suggest that DL imputers excel over linear models primarily in the simplistic unrealistic MCAR case where spatio-temporal pattens are not present, a method that has been used by previous benchmarking studies.

Additional Discussion A.2

When investigating existing effects between covariates and missingness, a general trend of increased missingness was observed among elderly patients in ICDEP. Several studies indicate that low peripheral perfusion, which is more common in older adults, can lead to higher rates of missing values in pulse oximetry signals[8]. Analyses also confirmed that low values of Spo2 < 70%, common in elderly patients, can lead to sensor malfunction and ultimately to data loss[9]. As findings from Haliduola et al. and Sondhi et al.[10,11] suggest, the post-hoc simulation of MNAR effects bases on these assumptions that less values are available for more extreme levels of clinical parameters. Hence, negative and positive MNAR effects were amplified with no change in general results, while the main manuscript reports imputation results where more unobserved values are present for lower value bins. In MIMIC and HIRID, missing values appeared more frequently in younger intensive care patients. A multi-center study[12] found that older adults ($\geq$ 75 years) were less likely to receive certain interventions (e.g., imaging or drainage) compared to younger patients. Similarly, younger patients ($\leq$ 65 years) made up the largest proportion in a study cohort investigating ventilated patients[13].

Either way, missingness patterns in clinical time series often have multi-factorial reasons which are impossible to tease out retrospectively. All three datasets MIMIC, HIRID, and ICDEP were also restricted to a selection of routinely collected EHRs, not providing the full cascade of actions resulting in sensor removal or medical interventions (complex inherent MNAR effects). As a limitation, we did not investigate transition probabilities conditional on workflow events. It is plausible that certain procedures affect missingness but we did not have fully access to these data or procedures (e.g. medical codes for surgical interventions) are documented after the hospital stay, discarding temporal dependencies. Real-world clinical missingness patterns might also depend on monitoring guidelines, the structure of the health-care system, staffing levels, hospital routines,

as well as additional patient related factors such as diagnosis, disease progression, and comorbidities[14]. Due to the selection of clinical data from Germany, the US, and Switzerland, detected missingness patterns are also limited to health systems in these countries. We are open for collaborations across other globally distributed medical centers to additionally validate our findings.

Additional Discussion A.3

We observed missing states where respiratory rate is abundantly unavailable ($\mathbf{s}$ = [0,0,0,0,1]), commonly found in the ICU dataset HIRID ($f = 0.23$). For patients in ICUs, who are mechanically ventilated, the configured respiratory rate is measured rather than the spontaneous breaths per minute[15]. These configurations do not change frequently, so clinical information systems store discontinuous mechanically configured respiratory rates, expressing data gaps that are often present in HIRID. The simultaneous absence of systolic and diastolic blood pressure (BP) values ($\mathbf{s}$ = [0,1,1,0,0]), primary observed in ICDEP ($f = 0.17$), is caused by measuring these two variables with the same sensor[16]. Since we dealt with a narrow equidistance time grid in ICDEP of 5 min intervals, more data gaps occurred (e.g., due to the BP cuff being inflated every 15 min) than in other ICU data sets. Spo2 values are found to be uniquely missing ($\mathbf{s}$ = [0,0,0,1,0]) with lower occurrence frequencies (e.g., $f = 0.036$ in MIMIC) than previous described missing patterns. Pulse oximetry is used for Spo2 measurements by attaching a clip to a patient's finger, these clips are oftentimes accidently detached or patients remove the sensor in agitated states[17,18]. Additional missingness patterns that are seldomly present in the extracted data (e.g., $\mathbf{s}$ = [1,0,1,0,1] with $f = 4.6E-6$ in MIMIC) could have occurred through monitoring transition issues, system errors, or fragmented extractions[19]. Hence, multiple authors call for primary source systems that are error robust, interoperable, and designed for secondary data analysis[20]. Besides, patient treatment in a bustling hospital environment also hampers continuous monitoring procedures that might have led to some rarely observed missing states[21].

References

1. Patrini, G. *et al.* Sinkhorn autoencoders. In *Uncertainty in Artificial Intelligence*, 733–743 (PMLR).
2. Cao, W. *et al.* BRITS: Bidirectional recurrent imputation for time series. 1805.10572[cs,stat].
3. Du, W., Côté, D. & Liu, Y. Saits: Self-attention-based imputation for time series. *Expert. Syst. with Appl.* **219**, 119619 (2023).
4. Wi, H., Shin, Y. & Park, N. Continuous-time autoencoders for regular and irregular time series imputation. In *Proceedings of the 17th ACM International Conference on Web Search and Data Mining*, 826–835 (2024).
5. Fan, Y. *et al.* Spatio-temporal denoising graph autoencoders with data augmentation for photovoltaic data imputation. **1**, 1–19, DOI: 10.1145/3588730.
6. Wang, X., Zhang, R., Sun, Y. & Qi, J. Doubly robust joint learning for recommendation on data missing not at random. In Chaudhuri, K. & Salakhutdinov, R. (eds.) *Proceedings of the 36th International Conference on Machine Learning*, vol. 97 of *Proceedings of Machine Learning Research*, 6638–6647 (PMLR, 2019).
7. Le Morvan, M., Josse, J., Scornet, E. & Varoquaux, G. What's a good imputation to predict with missing values? In Ranzato, M., Beygelzimer, A., Dauphin, Y., Liang, P. & Vaughan, J. W. (eds.) *Advances in Neural Information Processing Systems*, vol. 34, 11530–11540 (Curran Associates, Inc., 2021).
8. Poorzargar, K. *et al.* Accuracy of pulse oximeters in measuring oxygen saturation in patients with poor peripheral perfusion: A systematic review. **36**, 961–973, DOI: 10.1007/s10877-021-00797-8.
9. Elgendi, M. *et al.* The Striking Need for Age Diverse Pulse Oximeter Databases. **8**, 782422, DOI: 10.3389/fmed.2021.782422.
10. Haliduola, H. N., Bretz, F. & Mansmann, U. Missing data imputation using utility-based regression and sampling approaches. *Comput. Methods Programs Biomed.* **226**, 107172, DOI: https://doi.org/10.1016/j.cmpb.2022.107172 (2022).
11. Sondhi, A. *et al.* A systematic approach towards missing lab data in electronic health records: A case study in non-small cell lung cancer and multiple myeloma. **12**, 1201–1212, DOI: 10.1002/psp4.12998.
12. Margalit, I. *et al.* Presentation, management, and outcomes of older compared to younger adults with hospital-acquired bloodstream infections in the intensive care unit: A multicenter cohort study. **52**, 2435–2443, DOI: 10.1007/s15010-024-02304-y.
13. Ma, J.-G., Zhu, B., Jiang, L., Jiang, Q. & Xi, X.-M. Gender- and age-based differences in outcomes of mechanically ventilated ICU patients: A Chinese multicentre retrospective study. **22**, 18, DOI: 10.1186/s12871-021-01555-8.
14. Ayilara, O. F. *et al.* Impact of missing data on bias and precision when estimating change in patient-reported outcomes from a clinical registry. **17**, 106, DOI: 10.1186/s12955-019-1181-2.

15. Esteban, A. *et al.* How Is Mechanical Ventilation Employed in the Intensive Care Unit?: An International Utilization Review. **161**, 1450–1458, DOI: 10.1164/ajrccm.161.5.9902018.

16. Gibson, L. E. *et al.* Comparison of Invasive and Noninvasive Blood Pressure Measurements for Assessing Signal Complexity and Surgical Risk in Cardiac Surgical Patients. **130**, 1653–1660, DOI: 10.1213/ANE.0000000000003894.

17. Bowes, W. A., Corke, B. C. & Hulka, J. Pulse oximetry: A review of the theory, accuracy, and clinical applications. **74**, 541–546. 2668828.

18. Nguyen, H. *et al.* Reducing pulse oximetry false alarms without missing life-threatening events. **9–10**, 287–296, DOI: 10.1016/j.smhl.2018.07.002.

19. Kramer, O., Even, A., Matot, I., Steinberg, Y. & Bitan, Y. The impact of data quality defects on clinical decision-making in the intensive care unit. **209**, 106359, DOI: 10.1016/j.cmpb.2021.106359.

20. Vorisek, C. N. *et al.* Towards an Interoperability Landscape for a National Research Data Infrastructure for Personal Health Data. **11**, 772, DOI: 10.1038/s41597-024-03615-3.

21. Wilson, M. Common errors in clinical measurement. **22**, 197–201, DOI: 10.1016/j.mpaic.2021.01.001.
