# Supplementary material for: Benchmarking imputation methods on real-world clinical time series with simulated spatio-temporal missingness
Source: Nat Commun. 2026 Oct 5;17:10491. doi: 10.1038/s41467-026-77303-8 (PMC13639038; doi:10.1038/s41467-026-77303-8)
Supplement: Supplementary file 2 [file 41467_2026_77303_MOESM2_ESM.pdf]

nature portfolio

Peer Review File

Benchmarking imputation methods on real-world clinical time series with simulated spatio-temporal missingness

Corresponding Author: Mr Niklas Giesa

This file contains all reviewer reports in order by version, followed by all author rebuttals in order by version.

Version 0:

Reviewer comments:

Reviewer #1

(Remarks to the Author)
The authors introduced a novel approach to generating spatio-temporal missing data using Markov chains. Using three real-world applications, they induced missing data and compared the performance of various single imputation methods on these datasets. While this manuscript could be of interest to those applying machine learning approaches for prediction modelling, the generalisability of the results limited to chosen applications. My comments are as follows.

Major comments:

I recommend referring to Rubin's terminology of 'missing data mechanism' rather than patterns of missing data in the Introduction (page 1). The later refers to the location of missing data, whereas the former concerns the reasons for missing data.

As far as I see, multiple imputation (MI) approach has not been applied. Under MI, several imputed datasets are produced for each incomplete dataset. The downstream predication task is then performed separately on each imputed dataset, and the results are subsequently pooled using Rubin's rule. It seems that the authors used the MICE algorithm to create only a single imputed dataset. I therefore avoid calling the method 'multiple imputation'.

I disagree with the statement that the MICE approach cannot learn from temporal correlations. In fact, the conditional model for feature j at time t, for instance, can incorporate previously observed values of the same feature (at times 0 to t-1, or even future data points) when imputing feature j at time t. I would be valuable to include this version of MICE in the comparison across methods.

Minor comments:

Figure 2d may be confusing because rows don't sum up to 1, contrary to what is stated in the legend and the text. Please clarify.

As I understand, the MAR mechanism is defined for the total absent state (TAS) which depends on age. Due to the Markov process, all preceding states would therefore also depend on age. If this interpretation is correct, it should be clarified in the manuscript.

(Remarks on code availability)

Reviewer #2

(Remarks to the Author)
The paper proposes a Markov chain–based simulator that induces realistic spatio-temporal missingness in clinical time series from MIMIC, HiRID, and an intraoperative cohort (ICDEP). It benchmarks 20 imputation methods, from simple linear

interpolation and LOCF to spatio-temporal autoencoders, and evaluates both imputation quality and downstream classification. The central finding is that linear interpolation (non-causal) and LOCF (causal) are the most robust under realistic spatio-temporal missingness, whereas deep STAE models excel mainly when structure is absent (MCAR-like masking). Downstream AUROC/AUPRC differences across imputers are modest.

Strengths

The Markov construction over binary temporal slices, with empirically estimated initial/transition probabilities and MAR/MNAR amplifications, is a crisp and reproducible way to mimic patterns observed in practice.

The study convincingly shows that linear interpolation and LOCF outperform STAE-style deep models when missingness has realistic temporal/spatial structure.

Beyond error metrics (NRMSE/NWSD/CCorE), the paper measures predictive impact (AUROC/AUPRC) on mortality (MIMIC/HiRID) and POD (ICDEP), finding small spreads across imputers—useful for practitioners.

Weaknesses
The work is primarily empirical and lacks theoretical justification for when simple imputers should dominate.
Suggestion: Add a theory appendix with (i) identifiability/consistency conditions for the chosen error metrics under Markov missingness; (ii) bias decompositions for LOCF/linear interpolation versus STAE under MAR/MNAR; and (iii) simple risk bounds in linear-Gaussian or AR(1) settings with block missingness.

Several influential families are missing (masked-modeling Transformers such as BRITS/SAITS, continuous-time ODE/CDE methods, diffusion-based imputers like CSDI). In addition, training deep models on LOCF-prefilled inputs may disadvantage them.
Suggestion: Expand the benchmark to include these families and add a mask-only training regime (no prefill; explicit mask channel; loss computed only on missing entries).

Linear interpolation is non-causal while LOCF is causal; mixing them obscures use-case distinctions (retrospective vs. online).
Suggestion: Report a strictly causal evaluation where only past information is allowed, and contrast it with an offline setting. Clarify which results correspond to which scenario.

The MNAR setup conditions on ground-truth value bins post-simulation, which may be a coarse proxy for clinical censoring.
Suggestion: Consider generative censoring rules tied to physiologic thresholds or workflow events (e.g., sensor removal under certain interventions), and include sensitivity analyses to these choices.

Do your main conclusions hold when restricting evaluation to causal imputers (no access to the future)? How do rankings change relative to the offline setting?

Overall, a good and timely paper with clear practical relevance.

(Remarks on code availability)
The codebase is present and aligned with the paper, and the README documents dataset sources and high-level model information.

Reviewer #3

(Remarks to the Author)
Summary
This manuscript introduces a benchmark framework for evaluating time-series imputation methods in clinical datasets. The authors propose a simulation model that induces realistic spatio-temporal missingness patterns using Markov chains derived from empirical transition probabilities. They apply 20 imputation techniques—including simple statistical methods, MICE variants, and deep spatio-temporal autoencoders—and assess both reconstruction accuracy and downstream prediction performance across three datasets (MIMIC, HIRID, ICDEP). The experimental results indicate that deep learning methods outperform traditional approaches under MCAR conditions, whereas classical methods achieve superior performance under MAR and MNAR scenarios. The study addresses an important gap in the literature, namely the lack of benchmarks that reflect structured missingness in clinical time series.

Strong Points：
1. Innovative Spatio-Temporal Missingness Simulation Model: For the first time, Markov chain sampling is adopted to generate spatio-temporal missing values that are close to real clinical scenarios, breaking the limitation of previous studies focusing mostly on MCAR patterns. It accurately restores the missing correlations between features and over time in clinical data, providing a reliable basis for real-scenario testing of imputation methods.
2. Comprehensive Evaluation System for Imputation Methods: A total of 20 imputation methods are systematically included,

covering four categories: mean/median, interpolation/extrapolation, multiple imputation by chained equations, and deep learning. It includes both classic simple methods and cutting-edge spatio-temporal autoencoders, with a comprehensive comparison dimension, offering abundant references for method selection in different scenarios.
3. Support from Real and Diverse Clinical Datasets: Three real clinical datasets with different sources and complementary scenarios are adopted, covering routine ICU monitoring and intraoperative monitoring scenarios. The data scale is large and features are standardized, improving the external validity and generalizability of the research conclusions.

However, the paper exhibits several non-negligible limitations: the clinical rationality of some simulated missing states is unvalidated, the hyperparameter tuning strategy lacks sufficient justification, the amplification factors for MAR/MNAR are subjectively set without clinical basis, the evaluation metrics lack clinical significance alignment, and the generalizability is constrained by the Western-centric datasets and limited feature scope. These shortcomings weaken the robustness and persuasiveness of the conclusions.

Weak Points：
1. Rationality of Missingness Simulation States: The paper defines 32 spatio-temporal missing states but fails to verify whether all states have real clinical significance. The clinical relevance of some low-frequency states remains unclear, potentially disconnecting simulated scenarios from actual clinical data.
2. Sufficiency of Hyperparameter Tuning for Imputation Methods: Grid search and Hyperband search are used for hyperparameter tuning of autoencoders, but the rationale for designing the search space is not explained. Additionally, the impact of different tuning strategies on model performance is not compared, which may result in suboptimal parameters being overlooked.
3. Limitations of Clinical Relevance in Downstream Tasks: The downstream tasks only include in-hospital mortality and postoperative delirium, excluding more refined clinical outcomes. It is difficult to verify the impact of imputation methods on complex clinical decisions, and the effect of imputation errors on clinical risk stratification thresholds is not analyzed.
4. Subjectivity of Amplification Factors for MAR/MNAR: In the MAR and MNAR scenarios, after applying amplification to the missing data, does the resulting missingness pattern still correspond to realistic clinical settings? How is it ensured that the amplification is not excessive to the extent that too much original information is removed? If such amplification is excessive, and the dataset without amplification more closely reflects real-world conditions, then deep learning methods would, in fact, remain the more effective approach. In addition, the setting of age amplification factors for MAR amplification and feature value amplification factors for MNAR amplification lacks clinical basis. The association between factor selection and real clinical missing mechanisms is not explained, and the sensitivity of results to different factor combinations is not verified.
5. Lack of Clinical Significance in Evaluation Metrics: Existing evaluation metrics focus on statistical errors but do not design indicators combined with actual clinical significance, making it impossible to reflect the applicability of imputation results to clinical practice.

Below are some detailed comments.
1. The first-order Markov assumption for missingness transitions may oversimplify real-world dependencies. Clinical data often exhibit multi-step or periodic patterns of missingness. A brief sensitivity analysis or discussion of this limitation would strengthen the methodological rigor.
2. Selection of imputation methods. The survey of deep learning–based approaches appears insufficient. All deep learning methods in this study are based on the denoising autoencoder (DAE) architecture. There are, however, other relevant architectures for imputation, such as transformer-based [1], diffusion-based [2], and CNN-based [3] models, which have been applied to datasets like Physionet [4]. Please clarify the rationale for not including these alternative deep learning approaches in this work.
3. The amplification of MAR/MNAR effects through manually chosen scaling factors (e.g., age- or value-based multipliers) appears somewhat arbitrary. The authors should provide empirical justification (e.g., observed correlations in the raw data) or theoretical grounding based on prior literature.
4. The main conclusion—that simple linear methods perform robustly under realistic missingness—while practically useful, is not conceptually new. The discussion section should go beyond reporting empirical rankings to offer mechanistic insights into model behavior. For example, why do deep models degrade under structured missingness but excel under MCAR? A deeper analysis would make the study more valuable.
5. Generation of complete case datasets (CCD). According to the provided code, in preprocess.py, the function cut_na_sequences() appears to retain only the first complete sequence for each patient, even though a patient may have multiple complete sequences. Please explain the reason for restricting the selection to the first sequence. In addition, considering the CCD generation procedure and Figure A.2 in the Supplement, this choice might result in relatively short sequences, potentially limiting deep learning models' ability to effectively capture temporal dependencies. It could also affect downstream task performance—for example, using early sequences to predict final outcomes may yield poorer results compared to using later sequences. This may partly explain why different methods show limited variation in downstream performance. The construction of the "complete case dataset" (CCD) as ground truth introduces potential selection bias, as it retains only patients with short continuous segments. The authors should discuss how this might affect the generalizability and realism of the results.
6. The decision to focus on only five vital sign features significantly limits the scope of the benchmark. It would be valuable to test whether the findings hold when including additional intervention variables.
7. Computational efficiency is central to benchmarking. The authors should report approximate training times, hardware configurations, and resource requirements for deep models to support reproducibility.
8. The description of the transition matrix is somewhat confusing. In a Markov chain, each row of the transition matrix should sum to 1, representing a valid conditional probability distribution of next-state transitions given the current state. It is unclear whether the authors normalized rows independently or the entire matrix globally. Clarification and consistency checks are needed, as this directly affects the validity of the simulated missingness process.

9. The term spatio-temporal may be misleading in this context, as the data under study are multivariate clinical time series without true spatial coordinates. A more accurate term might be “multivariate time series” or “cross-feature time series.” The authors should clarify or adjust the terminology accordingly to avoid conceptual confusion.
10. Correct minor grammatical and stylistic issues (e.g., “it’s value levels” → “its value levels”).

References:
[1] Nie T, Qin G, Ma W, Mei Y, Sun J. ImputeFormer: Low Rankness-Induced Transformers for Generalizable Spatiotemporal Imputation[C/OL]//Proceedings of the 30th ACM SIGKDD Conference on Knowledge Discovery and Data Mining. 2024: 2260-2271[2025-02-27]
[2] Yuan X, Qiao Y. Diffusion-TS: Interpretable Diffusion for General Time Series Generation[C]//The Twelfth International Conference on Learning Representations.
[3] Wu H, Hu T, Liu Y, Zhou H, Wang J, Long M. TimesNet: Temporal 2D-Variation Modeling for General Time Series Analysis[C]//The Eleventh International Conference on Learning Representations.
[4] Wang J, Du W, Cao W, Zhang K, Wang W, Liang Y, Wen Q. Deep Learning for Multivariate Time Series Imputation: A Survey[A/OL]. arXiv, 2024[2024-02-22]. http://arxiv.org/abs/2402.04059.

(Remarks on code availability)
The provided repository includes model descriptions and references to datasets, but lacks practical instructions for reproducing the reported results. While a short code snippet for model loading is given, there are no installation guidelines, data preprocessing steps, or example commands for training or evaluation. Thus, the current repository is insufficient for full reproducibility without additional documentation or scripts.

Version 1:

Reviewer comments:

Reviewer #1

(Remarks to the Author)
I thank the authors for carefully addressing my previous comments, which has improved the readability of the manuscript. The paper will certainly be of interest to readers of Nature Communications.

Although the authors have responded to my points in their response letter, some critical issues have not yet been fully reflected in the revised manuscript. It is essential that accurate terminology is used when presenting the results; otherwise, the manuscript may convey misleading or incorrect messages to readers. I highlight two important remaining points:

1- As noted in the previous review round and acknowledged by the authors, a multiple imputation (MI) approach has not been applied in this study. As far as I can see, all imputation methods considered (over 20 methods) are single imputation approaches. It is therefore inaccurate to state that MI has been used (e.g., in Table 3). The authors should avoid this terminology throughout the manuscript. In addition, MICE refers to “multivariate imputation using chained equations,” and one cycle of the MICE algorithm produces one imputed dataset.

2- Similarly, while the authors confirmed that MICE can learn from temporal correlations (a variant they called MICET), Table 3 still states that MICE cannot condition on previous recordings of a feature. This is incorrect and should be revised accordingly throughout the manuscript.

(Remarks on code availability)

Reviewer #2

(Remarks to the Author)
This revised manuscript presents a timely and practically relevant benchmark for imputation in clinical time series. The paper introduces a Markov chain-based simulator to generate realistic spatio-temporal missingness patterns from real clinical data and evaluates a broad range of imputation methods across three datasets. The study addresses an important gap in the literature, since many previous benchmarks rely on oversimplified MCAR-style masking and therefore may overestimate the benefit of complex deep learning imputers. In contrast, this work demonstrates that under more realistic structured missingness, simple approaches such as linear interpolation and LOCF remain highly competitive and often outperform more sophisticated spatio-temporal autoencoder models, while downstream prediction performance differences across imputers are generally modest.

(Remarks on code availability)
This revised manuscript presents a timely and practically relevant benchmark for imputation in clinical time series.

I am very satisfied with the revised manuscript. After revision, my previous concerns are largely resolved. I therefore support publication of this work.

Reviewer #3

(Remarks to the Author)
Overall, the authors have provided clear responses to all comments raised by Reviewer #3, and most of the suggested revisions have been properly incorporated into the main manuscript and the Supplementary Materials. The quality of the manuscript has improved accordingly. However, a few minor issues remain and could be further refined.

1. In the response to Weak point 3, the referenced figure should be Figure A.3 rather than Figure A.4. This appears to be a minor typographical error and does not affect the overall results.

2. Regarding Detailed Comment 3, the design of MAR and MNAR amplification relies on scaling factors and thresholds (e.g., the 90% discussed in Additional Discussion A.2), which appear somewhat heuristic. While this is understandable given the complexity of real-world clinical missingness, it remains unclear how sensitive the results are to these choices. In the current manuscript, the experiments seem to rely on a single configuration of these scaling parameters. It would strengthen the work if the authors could provide a sensitivity analysis with respect to different scaling factors (or threshold values), at least for a subset of representative imputation methods. This would help assess the robustness of the reported conclusions under varying missingness intensities.

3. Regarding Detailed Comment 8, there appears to be an inconsistency in the description of the transition probabilities. On page 5, the manuscript states that “The relative frequencies of occurrence and transition states were retrieved and normalized globally for the entire population across all time series,” which is consistent with the presentation in Figure 6(d). However, in the Simulation Model section on page 15, it is stated that the transition probabilities are defined “with rows summing up to one (see Figure 6d).” This creates ambiguity regarding how the transition probabilities are actually computed. I recommend that the authors clarify this point by providing a formal definition or equation, and explicitly explain why a standard row-normalized transition matrix (i.e., each row summing to one) is or is not used.

(Remarks on code availability)

Version 2:

Reviewer comments:

Reviewer #1

(Remarks to the Author)
I thank the authors for their careful revision and for addressing my remaining concerns. I have no further comments.

(Remarks on code availability)
see above.

Reviewer #3

(Remarks to the Author)
The authors have substantially addressed my remaining concerns. In particular, they added sensitivity analyses under different MAR/MNAR amplification settings and clarified how transition probabilities are estimated in the Markov-chain simulator. I am satisfied with the revised manuscript and therefore support publication of this work.

I only note one minor editorial inconsistency in the rebuttal: the MNAR reverse setting is first described as using scaling factors of {3.4, 2.4, 1.4}, whereas the later quoted text for Additional Results A.4 states that scaling factors of {1.8, 2.8, 3.8} were used for the MNAR reverse setting. Please check and ensure that the reported scaling factors are consistent throughout the rebuttal and Supplementary Materials. This minor issue does not affect the main conclusions.

(Remarks on code availability)

Open Access This Peer Review File is licensed under a Creative Commons Attribution 4.0 International License, which permits use, sharing, adaptation, distribution and reproduction in any medium or format, as long as you give appropriate credit to the original author(s) and the source, provide a link to the Creative Commons license, and indicate if changes were made.
In cases where reviewers are anonymous, credit should be given to 'Anonymous Referee' and the source.
The images or other third party material in this Peer Review File are included in the article's Creative Commons license, unless indicated otherwise in a credit line to the material. If material is not included in the article's Creative Commons license and your intended use is not permitted by statutory regulation or exceeds the permitted use, you will need to obtain permission directly from the copyright holder.
To view a copy of this license, visit https://creativecommons.org/licenses/by/4.0/

We thank all reviewers for their comprehensive feedback and comments. We hope to fully address all concerns raised by the reviewers and provide a revised manuscript alongside additional comprehensive ablation studies in the Supplementary file.

Reviewer #1 (Remarks to the Author)

The authors introduced a novel approach to generating spatio-temporal missing data using Markov chains. Using three real-world applications, they induced missing data and compared the performance of various single imputation methods on these datasets. While this manuscript could be of interest to those applying machine learning approaches for prediction modelling, the generalisability of the results limited to chosen applications.

RESPONSE #1.1: We thank Reviewer #1 for providing valuable feedback. As correctly indicated, we applied a Markov model to clinical data for inducing realistic spatio-temporal patterns of missingness from real-world data. Our study shows that deep learning imputers are especially effective when imputing clinical time series under the MCAR missingness assumption, which is unrealistic in the clinical domain [1]. However, deep learning imputers fail for more realistic spatio-temporal assumptions. In general, the methodology of our study is applicable to other non-medical domains (e.g., finance, traffic, GIS) where spatio-temporal relations are present in multivariate time series, currently out of scope for our experiments. To the point, we trained a CNN-based autoencoder proposed by Fan et al. [2] which was originally trained with time series from photovoltaic systems. Authors like Wu et al. [3], Zhang et al. [4], or Qian et al. [5] applied similar spatio-temporal autoencoder to fill unobserved values in multivariate time series from the traffic or the geographic information system (GIS) domain.

In our **Discussion** section, we added the following to the manuscript: "While we applied our Markov chain model to clinical multivariate time series, additional domains where spatio-temporal missingness patterns are a burden (e.g., traffic or geographic data) can benefit from our novel simulation approach."

[1] Tan, A. L., Getzen, E. J., Hutch, M. R., Strasser, Z. H., Gutiérrez-Sacristán, A., Le, T. T., ... & Holmes, J. H. (2023). Informative missingness: What can we learn from patterns in missing laboratory data in the electronic health record?. *Journal of biomedical informatics*, *139*, 104306.
[2] Fan, Y., Yu, X., Wieser, R., Meakin, D., Shaton, A., Jaubert, J. N., ... & Wu, Y. (2023). Spatio-temporal denoising graph autoencoders with data augmentation for photovoltaic data imputation. Proceedings of the ACM on Management of Data, 1(1), 1-19.
[3] Wu, J., Piao, X., Wei, X., & Zhang, Y. (2025). STCDM: Spatio-Temporal Conditional Diffusion Model for Traffic Data Filling. IET Intelligent Transport Systems, 19(1), e70016.
[4] Zhang, X., & Zhou, P. (2024). A transferred spatio-temporal deep model based on multi-LSTM auto-encoder for air pollution time series missing value imputation. Future Generation Computer Systems, 156, 325-338.
[5] Qian, J., Zhang, S., Pian, Y., Chen, X., & Liu, Y. (2025). Spatiotemporal subspace variational autoencoder with repair mechanism for traffic data imputation. Neurocomputing, 617, 128948.

Major comments:

I recommend referring to Rubin's terminology of 'missing data mechanism' rather than patterns of missing data in the Introduction (page 1). The later refers to the location of missing data, whereas the former concerns the reasons for missing data.

RESPONSE #1.2: We rephrased Rubin's terminology of 'missing data mechanism' as you suggested and ensured consistent naming through the entire manuscript. In principle, we aimed to simulate spatio-temporal patterns that we measured in real-world time series. In real-world clinical data, these patterns cannot be proved to be strictly conditioned on any missing data mechanisms by Rubin. Hence, we

established controlled scenarios where we actively conditioned detected missingness patterns on the covariate age (MAR mechanism) or the values of one selected feature (MNAR mechanism).
As far as I see, multiple imputation (MI) approach has not been applied. Under MI, several imputed datasets are produced for each incomplete dataset. The downstream predication task is then performed separately on each imputed dataset, and the results are subsequently pooled using Rubin's rule. It seems that the authors used the MICE algorithm to create only a single imputed dataset. I therefore avoid calling the method 'multiple imputation'.

RESPONSE #1.3: We completely agree with Reviewer#1 that the original version of multiple imputations (MI) has not been applied to our data since we did not distinctly analyze multiple imputed sets of data within one imputation method.

We now clarify the distinction between multiple imputation by chained equations (MICE) and MI with the following sentence in our **Methods**: "We applied the well-known MICE algorithm as iteratively improving imputations in one dataset by using chained equations. We did not include a classical multiple imputation (MI) approach where multiple distinctly imputed sets are produced. Hence, downstream tasks were also not compared across different MI imputed sets." We further estimate the variance of our downstream results by bootstrapping and cross validation which helps to quantify the robustness of methods across different testing sets.

I disagree with the statement that the MICE approach cannot learn from temporal correlations. In fact, the conditional model for feature j at time t, for instance, can incorporate previously observed values of the same feature (at times 0 to t-1, or even future data points) when imputing feature j at time t. I would be valuable to include this version of MICE in the comparison across methods.

RESPONSE #1.4: We really appreciate the proposed model variant of MICE. As suggested, we implemented MICE as a new time causal variant MICET so that it operates on a conditional feature space holding data up to time t to impute corresponding values. We agree that MICE can learn from temporal correlations by explicitly including lagged values of a time series. Future values can potentially also be incorporated in the MICE algorithm, if temporal causality isn't required.

We introduced MICET to the **Methods** section with "In addition to the standard MICE method, we also evaluated an enhanced temporal version, denoted as MICET, where the input space is conditioned on previously measured recordings for each feature (see Table A.11 Supplement A)"

The newly implemented method MICET achieved following scores in cross-correlation error (CCorE), normalized root mean squared error (NRMSE), and normalized Wasserstein distance (NWSD):

Dataset	Amplification	CCorE_loc	NRMSE_loc	NWSD_glob
MIMIC	none	12.739±0.211	9.283±0.081	0.032±0.010
	MAR	21.218±0.244	14.868±0.100	0.244±0.121
	MNAR	23.775±0.184	12.214±0.082	0.077±0.022
HIRID	none	6.600±0.108	17.047±0.278	0.160±0.172
	MAR	13.875±0.193	20.772±0.264	0.420±0.388
	MNAR	11.559±0.119	18.842±0.297	0.334±0.352
ICDEP	none	12.371±0.184	11.825±0.130	0.051±0.020
	MAR	22.860±0.243	15.483±0.120	0.307±0.115
	MNAR	17.120±0.187	9.498±0.099	0.067±0.031

Table A.11. **Performance metrics for temporally enhanced MICE imputation methods.** The MICE method was enhanced so the input space is conditioned on previously measured recordings for each feature. Results are reported in % with mean±95%CI across time series, features, and validation folds.

We added new results to our main manuscript and we report additional imputation metrics in the **Supplement A**.

Minor comments:

Figure 2d may be confusing because rows don't sum up to 1, contrary to what is stated in the legend and the text. Please clarify.

RESPONSE #1.5: In the transition matrix M sums of probabilities across both rows and columns are required to sum to 1, we apologize for this misconception and now write "we retrieved a transition table across all patient time series covered by our population where rows and columns (all probabilities for all consecutive states) jointly sum up to one" in the caption of Figure 2d.

As I understand, the MAR mechanism is defined for the total absent state (TAS) which depends on age. Due to the Markov process, all preceding states would therefore also depend on age. If this interpretation is correct, it should be clarified in the manuscript.

RESPONSE #1.6: We thank Reviewer#1 for this comment and we agree. All transition states under the MAR scenario follow an age-specific alternation of the probability for the TAS. To clarify this point, we added "By increasing the probability of TAS for elderly patients, all subsequent transition states which were sampled from the Markov chain depend on age, reflecting a controlled simulation scenario for induced MAR effects." to the **Methods** section.

(Remarks on code availability)

Reviewer #2 (Remarks to the Author)

The paper proposes a Markov chain–based simulator that induces realistic spatio-temporal missingness in clinical time series from MIMIC, HiRID, and an intraoperative cohort (ICDEP). It benchmarks 20 imputation methods, from simple linear interpolation and LOCF to spatio-temporal autoencoders, and evaluates both imputation quality and downstream classification. The central finding is that linear interpolation (non-causal) and LOCF (causal) are the most robust under realistic spatio-temporal missingness, whereas deep STAE models excel mainly when structure is absent (MCAR-like masking). Downstream AUROC/AUPRC differences across imputers are modest.

Strengths

The Markov construction over binary temporal slices, with empirically estimated initial/transition probabilities and MAR/MNAR amplifications, is a crisp and reproducible way to mimic patterns observed in practice.

The study convincingly shows that linear interpolation and LOCF outperform STAE-style deep models when missingness has realistic temporal/spatial structure.

Beyond error metrics (NRMSE/NWSD/CCorE), the paper measures predictive impact (AUROC/AUPRC) on mortality (MIMIC/HiRID) and POD (ICDEP), finding small spreads across imputers—useful for practitioners.

Weaknesses
The work is primarily empirical and lacks theoretical justification for when simple imputers should dominate.

RESPONSE #2.1: We appreciate the extensive comments by Reviewer#2. Our manuscript substantially benefited from additional reference models (e.g., BRITS or SAITS) that expanded our empirical evaluation of deep learning imputers. Additionally, we included further justifications of missing scenarios and imputation methods in the revised version of our manuscript.

Suggestion: Add a theory appendix with (i) identifiability/consistency conditions for the chosen error metrics under Markov missingness; (ii) bias decompositions for LOCF/linear interpolation versus STAE under MAR/MNAR; and (iii) simple risk bounds in linear-Gaussian or AR(1) settings with block missingness.

RESPONSE #2.2: We thank Reviewer#2 for requesting additional theory about identifiability and consistency conditions. As we understand, identifiability is the capability that model parameters can be uniquely recovered having access to infinite data from the data-generating process. Here, we unfortunately do not have access to such data generating process of highly complex clinical time series. Consistency further describes the behavior of an estimator that if the amount of finite data increases, the estimated parameters converge towards the true model parameters [1].

We are not completely sure what the comment (i) aims to address with respect to identifiability or consistency properties (error metrics, imputation methods, or Markov model). Reported error metrics did not require parametrization that potentially change, dependent on finite or infinite data. In our study, metrics such as the mean absolute error (MAE), mean squared error (MSE) or the Wasserstein distance (WSD) were used to quantify differences between imputed data and GT, rather than using them to evaluate a parameter fit to generative time series model. If applied to parameters of data models, minimizing MAE and MSE results in identifying the mean and median consistently with growing sample size [2,3]. Berton et al. [2] show that the minimum Wasserstein distance estimator (MWDE) is identifiable and consistent if different parameters result in different data distributions. However, we

cannot prove these properties in our experiments since we rather simulate missingness patterns in clinical time series than the true underlying data.

If mean and median imputers are applied to data that strictly follow MCAR, these imputers can be considered identifiable and consistent [5,6]. In MNAR or MAR cases, the training data does otherwise not reflect a true sample from the data generating process and minimizing MAE or MSE can lead to inconsistent and biased estimates, deviating from the mean or median. The novel simulation method proposed by our study induces realistic spatio-temporal missingness patterns that do not strictly follow a MCAR logic. Furthermore, we believe that our proposed Markov model is identifiable due to the estimation of transition probabilities over a discrete unique state space and consistently converges to the true underlying probabilities if the sample seize increases [7].

Saha and Jones [8] demonstrate that LOCF is neither consistent nor identifiable when data dropout is applied to simulate MCAR and MNAR in longitudinal clinical data, failing to reflect data population properties. Due to linear interpolation being a deterministic algorithm that constructs a unique piecewise data function (unique slope), it is usually considered identifiable [9]. Consistency does only hold for linear interpolation if the underlying generated data do follow linear distributions and the data is free of noise [10]. Both of these constraints are not applicable for our clinical data. MICE imputers are only identifiable when very strict assumptions hold: Saeed et al. emphasize that the joint distribution of data as well as missingness indicators must be identifiable and that these assumptions are violated in MNAR cases [11]. Due to MICE depending on multiple iterations of regression tasks, the method probably does not settle on a stable data distribution in the long run that can produce inconsistent results [12].

Neural network based autoencoders are usually highly parametrized and are trained via stochastic processes where the loss can fall in local minima. Thus, identifiable and consistency assumptions are difficult to prove for these models. Current research by Khemakhem et al. [13] and Mattei et al. [14] argue that spatio-temporal autoencoders can be identifiable if the latent space is conditioned on an auxiliary variable (e.g., the time index) and that in some regularized model architectures, model weights can be obtained consistently. However, model parameters are usually not identifiable because different unique sets can produce the same output and the latent compression in autoencoders prevents the model from consistent estimates regardless of the amount of data.

We sincerely thank Reviewer#2 to direct our work to this very interesting field of research, but we believe that further comprehensive studies would be required to fully address this comment. In the **Discussion**, we now argue:

“While related studies demonstrate that identifiability and consistency are guaranteed for simple averaging techniques under restrictive MCAR assumptions, these properties remain theoretically elusive for deep learning architectures like autoencoders. We posit that our Markov missingness model provides a robust method for simulating realistic missingness patterns beyond MCAR assumptions, yielding consistent estimates of transition probabilities that converge to the true missingness process as the sample size increases. Further research is required to explore the behavior of autoencoders, imputing such realistic missingness patterns in clinical time series.”

We also thank the Reviewer#2 for requesting more information about bias decompositions in comment (ii). As shown in [15], bias decomposition investigates the expected generalization error that is decomposed in a systematic error across samples (bias), a variance term for estimating explicit bias in training data, and irreducible noise. LOCF and linear interpolation do not rely on pre-training, they also do not infer global parameters, such as means. Hence, these models produce zero variance across multiple runs on the same dataset and minimal statistical variance across different samples. For longitudinal time series, both methods are expected to result in high bias (systematic error) because LOCF does not reflect fluctuating trends across time and linear interpolation does not account for non-

linear periodic patterns. On the contrary, STAE are expected to result in little bias due to the fitting to spatio-temporal dependencies in longitudinal data, but they can introduce high variance due to the high dimensionality of training data [16]. Unfortunately, we do not have a well-justified model for the data generating process, meaning that bias decomposition depends on observations which include noise. Realistic estimations of bias, variance, and noise are therefore impossible to obtain.

Comment (iii) addresses risk bounds for a linear Gaussian or AR(1) data generating process. We detected complex patterns of missing values in real-world clinical time series which diversely fluctuate without following simplified Gaussian linear or AR behavior. The detection of original spatio-temporal patterns allowed us to reflect existing block-missingness patterns. These patterns are further compared between original and simulated data in the **Supplementary A** in **Table A.3.**

To describe general bounds of reported error metrics, we indicate that our clinical time series were processed so that data fall into value bounds that are physiologically reasonable.

In the **Methods**, we write: “Similar to previous studies, we used data ranges of [5, 400] bpm for hr, [2, 300] bpm for rr, [10, 100] % for spo2, [20, 400] mmHg for bp_sys, and [10, 300] mmHg for bp_dia to detect outliers. Feature values that fall outside these defined ranges are marked as outliers. The first cleaning strategy preserved the original spatio-temporal missingness by setting outliers to the nearest edges of the valid ranges, e.g, 120% Spo2 was set to 100%. Thereby, outliers were corrected instead of removed from the dataset. (…) In contrast, the second strategy set outliers to missing”

Hence, the root-mean squared error (RMSE) is bounded by the maximum distance between feature values of 395 (here, 400 - 5 = 395 mmHg for bp_sys), the minimum distance between values across features is 90 (here, 100 – 10 = 90 % for spo2), while an RMSE value of zero indicates that the imputation was perfect. Similarly, the mean absolute error (MAE) as well as the Wasserstein distance (WSD) are bounded. For auto-correlation (ACor) and cross-correlator (CCor), their levels were bounded from -1 to 1 due to the inherent usage of correlation coefficients.

However, these global data bounds are independent of both the choice of imputers and the underlying data properties or missingness patterns. For LOCF, KNN, averaging methods, and linear interpolations, these bounds cannot be exceeded. Other imputation methods such as deep learning autoencoders or quadratic imputers might result in data beyond these bounds. To expand the **Methods**, we added:

“While error metrics (MSE, RMSE, WSD) are naturally bounded by the valid data ranges that are used to detect and remove outliers in the original data, all reported metrics are normalized so that errors fall into a range between zero and one.”

To expand our clinical analysis, we also added the MAE and RMSE without normalization in the Supplementary A and grouped these errors by feature and datasets. We also added the following aspects to the **Discussion**:

“When assessing MAE and RMSE grouped by feature without normalization, systolic blood pressure (bp_sys) consistently yields the highest errors (max. of 13.58±0.42 MAE and 18.27±0.42 RMSE) (see Table A.10 in Supplement A). Elevated errors for bp_sys occur likely due to high variability and wider physiological range of blood pressure readings compared to more stable vitals (standard-deviation of max. 29.99 mmHg in CCD). Conversely, blood oxygen (spo2) exhibits the lowest errors (min. of 0.66±0.01 MAE and 1.34±0.04 RMSE), reflecting its narrower clinical range where even small deviations can be physiologically relevant. When comparing metrics, MAE values are notably higher than RMSE across all features, expressing a large deviation for respiratory rate (rr) recordings in HIRID (9.27±1.01 MAE, 28.45±3.67 RMSE for LINPOL). These deviations confirm increased sensitivity to large outliers for high-frequency vital signs which, in a clinical context, often are caused by critical acute changes or sensor noise.”

		MAE			RMSE		
**feature**	**method**	**MIMIC**	**HIRID**	**ICDEP**	**MIMIC**	**HIRID**	**ICDEP**
bp_dia	1locf	7.93±0.21	4.03±0.12	9.85±0.23	11.52±0.37	7.77±0.16	13.81±0.29
	bilstaestat	7.15±0.32	5.58±0.82	8.75±0.38	9.91±0.52	8.23±0.6	11.74±0.59
	linpol	6.92±0.28	3.12±0.11	8.53±0.2	10.18±0.48	6.64±0.54	12.11±0.33
	lstae	7.93±0.16	5.29±0.19	9.46±0.18	10.85±0.29	8.07±0.19	12.5±0.21
bp_sys	1locf	12.09±0.11	7.24±0.2	14.44±0.31	16.63±0.24	11.41±0.24	20.14±0.36
	bilstaestat	11.15±0.05	10.96±1.55	12.71±0.81	14.85±0.15	14.78±1.42	17.37±0.83
	linpol	10.49±0.16	5.35±0.2	12.38±0.3	14.75±0.35	8.99±0.44	17.71±0.34
	lstae	12.53±0.19	10.84±0.52	13.58±0.42	16.56±0.41	14.81±0.35	18.27±0.42
hr	1locf	6.86±0.12	2.44±0.2	10.57±0.37	10.25±0.17	5.48±1.19	13.76±0.43
	bilstaestat	7.44±0.19	6.26±0.7	8.92±0.48	10.22±0.14	9.46±0.92	11.95±0.9
	linpol	5.9±0.1	1.82±0.19	9.46±0.29	9.18±0.17	4.64±1.23	12.9±0.55
	lstae	9.07±0.1	6.34±0.23	9.98±0.29	12.25±0.15	9.71±0.42	13.2±0.51
rr	1locf	3.6±0.05	9.31±1.03	2.22±0.05	5.01±0.04	30.51±3.9	3.55±0.55
	bilstaestat	3.12±0.03	13.25±1.15	1.83±0.03	4.19±0.06	34.72±2.56	2.98±0.37
	linpol	3.23±0.03	9.27±1.01	1.9±0.07	4.49±0.02	28.45±3.67	3.21±0.43
	lstae	3.43±0.11	12.62±1.96	1.86±0.05	4.57±0.14	32.82±5.51	3.01±0.4
spo2	1locf	1.68±0.06	0.83±0.01	0.86±0.02	2.97±0.28	1.69±0.16	1.38±0.04
	bilstaestat	1.55±0.02	1.38±0.09	0.84±0.03	2.6±0.13	2.28±0.24	1.29±0.01
	linpol	1.53±0.06	0.66±0.01	0.86±0.04	2.7±0.22	1.48±0.13	1.34±0.04
	lstae	1.72±0.05	1.38±0.07	0.96±0.02	2.74±0.12	2.25±0.19	1.4±0.03

Table A.10. **Mean absolute error (MAE) and root-mean squared error (RMSE) per feature**. For each feature, the local MAE and RMSE is displayed as mean±standard-deviation grouped by the imputation method and per dataset (MIMIC, HIRID, ICDEP). The reported summary statistics are calculated across all patient time series and across all cross-validation folds

[1] Gabrielsen, A. (1978). Consistency and identifiability. Journal of Econometrics, 8(2), 261-263.

[2] van der Vaart, A. W. (1998). Asymptotic Statistics. Cambridge Series in Statistical and Probabilistic Mathematics 3. Cambridge Univ. Press, Cambridge.

[3] Huber, Peter J., and E. M. Ronchetti. "Robust statistics. 2nd john wiley & sons." *Hoboken, NJ* 2 (2009).

[4] Bernton, E., Jacob, P. E., Gerber, M., & Robert, C. P. (2019). On parameter estimation with the Wasserstein distance. *Information and Inference: A Journal of the IMA*, *8*(4), 657-676.

[5] Joel, L. O., Doorsamy, W., & Paul, B. S. (2025). A comparative study of imputation techniques for missing values in healthcare diagnostic datasets. *International Journal of Data Science and Analytics*, 1-17.

[6] Zhou, Y., Aryal, S., & Bouadjenek, M. R. (2024). A Comprehensive Review of Handling Missing Data: Exploring Special Missing Mechanisms. *arXiv preprint arXiv:2404.04905*.

[7] Billingsley, P. (1961). *Statistical inference for Markov processes* (Vol. 2). Chicago: University of Chicago Press.

[8] Saha, C., & Jones, M. P. (2009). Bias in the last observation carried forward method under informative dropout. *Journal of Statistical Planning and Inference*, *139*(2), 246-255.

[9] Atkinson, K. E., & Han, W. (1993). *Elementary numerical analysis*. New York: Wiley.

[10] Zhang, H. (2004). Inconsistent estimation and asymptotically equal interpolations in model-based geostatistics. *Journal of the American Statistical Association*, *99*(465), 250-261.

[11] Karvanen, J., & Tikka, S. (2024). Multiple imputation and full law identifiability. *arXiv preprint arXiv:2410.18688*.

[12] Zhang, J., Dashti, S. G., Carlin, J. B., Lee, K. J., Bartlett, J. W., & Moreno-Betancur, M. (2024). Sensitivity analysis methods for outcome missingness using substantive-model-compatible multiple imputation and their application in causal inference. *arXiv preprint arXiv:2411.13829*.

[13] Khemakhem, I., Kingma, D., Monti, R., & Hyvarinen, A. (2020, June). Variational autoencoders and nonlinear ica: A unifying framework. In *International conference on artificial intelligence and statistics* (pp. 2207-2217). PMLR.

[14] Mattei, P. A., & Frellsen, J. (2019, May). MIWAE: Deep generative modelling and imputation of incomplete data sets. In *International conference on machine learning* (pp. 4413-4423). PMLR.

[15] Domingos, P. (2000, June). A unified bias-variance decomposition. In Proceedings of 17th international conference on machine learning (pp. 231-238). Morgan Kaufmann Stanford.

[16] Gruber, S. G., & Buettner, F. (2022). Uncertainty estimates of predictions via a general bias-variance decomposition. Proceedings of Machine Learning Research.

Several influential families are missing (masked-modeling Transformers such as BRITS/SAITS, continuous-time ODE/CDE methods, diffusion-based imputers like CSDI). In addition, training deep models on LOCF-prefilled inputs may disadvantage them.
Suggestion: Expand the benchmark to include these families and add a mask-only training regime (no prefill; explicit mask channel; loss computed only on missing entries).

RESPONSE #2.3: We thank the Reviewer#2 for suggesting additional deep learning imputers and we introduce suggested reference models in the **Introduction** section with:

"Fan et al. [1] proposed a CNN autoencoder architecture for processing time series via windowing, which involved a trainable graph for static attributes. Their proposed model was denoted as STGAE (Spatio-temporal Denoising Graph Autoencoder). The authors Cao et al. [2] proposed the model architecture BRITS (Bidirectional Recurrent Imputation for Time Series) which jointly learn imputations for multivariate time series as well as a downstream classification task. For the imputation of elements in temporal data, known values are masked and recurrently predicted by an LSTM cell. Similarly, the authors Du et al. [3] use masking of known values in time series that hold observed as well as unobserved data. Their model architecture SAITS (Self-Attention-based Imputation for Time Series) relies on self-attention modules instead of recurrent mechanisms. In 2018, Chen et al. [4] introduced the concept of neural ordinary differential equations (ODEs) that can be used to continuously model a latent space, improving discrete RNN representations. The CTA (continuous-time autoencoder) model by Wi et al. exploits ODEs that are applied on regression coefficients from cubic splines, fitted to multivariate time series."

Besides, we describe the training procedure and evaluation results for these reference models in the Supplementary A under Additional Results A.3 with:

"Due to the configuration of LSTM modules in BRITS, this model was trained with batch size of one and accumulating gradients, similar to the STAE models reported in the main manuscript. STGAE and SAITS were trained on time series that were padded to the longest sequence within one batch. CTA was adopted so that the model could also handle time series of unequal lengths. To inherently learn to impute time series via masking, 10% of the actual observed values inside the training set were masked out. Hereby, no LOCF pre-imputation was required for learning while only the imputed values contributed to the MSE loss. All reference model architectures were trained with published hyperparameter configurations [1-4].

The Table A.9 displays results from trained reference models (ref), which were all considered non-time causal since they infer statistics from future time points, in comparison to linear interpolation (LINPOL) and BiLSTM autoencoder (LSTM). In scenario **A),** models imputed patterns that strictly follow a MCAR mechanism without spatio-temporal structure, as done by previous authors that claim superiority of DL imputers over linear ones. In scenario **B),** models are applied on more complex and realistically simulated spatio-temporal missingness patterns that we detected in real-world clinical time series. For the oversimplified MCAR scenario, LINPOL yielded lowest globally computed NWSD scores for MIMIC and HIRID. However, more advanced models comprising the reference BRITS and the BILSTAE architecture resulted in lowest CCorD and NRMSE scores across all three datasets. In ICDEP, BILSTAE imputed with best evaluations in comparison to LINPOL as well as all reference models.

		A) MCAR induction without spatio-temporal missingness patterns			B) Markov chain simulation of spatio-temporal missingness patterns		
Data	Method	CCorD_loc	NRMSE_loc	NWSD_glob	CCorD_loc	NRMSE_loc	NWSD_glob
MIMIC	LINPOL	14.024±0.172	3.136±0.060	**0.140±0.038**	6.581±0.164	10.578±0.103	**0.090±0.016**
	BILSTAE	10.143±0.131	**1.947±0.046**	0.248±0.043	6.581±0.159	10.840±0.097	0.234±0.041
	SAITS (ref)	13.954±0.145	5.394±0.053	0.489±0.096	10.002±0.196	6.399±0.060	0.424±0.084
	BRITS (ref)	**9.067±0.125**	3.003±0.033	0.169±0.030	**6.220±0.156**	**3.926±0.041**	0.172±0.029
	CTA (ref)	13.875±0.178	5.205±0.065	0.623±0.157	9.916±0.245	6.369±0.077	0.532±0.135
	STGAE (ref)	9.125±0.159	16.225±0.135	0.417±0.209	13.730±0.256	24.327±0.202	0.554±0.160
HIRID	LINPOL	4.524±0.095	8.337±0.183	**0.043±0.040**	3.384±0.090	17.824±0.296	**0.026±0.024**
	BILSTAE	3.729±0.074	6.694±0.138	0.152±0.138	3.348±0.091	18.685±0.364	0.337±0.385
	SAITS (ref)	8.887±0.100	31.965±0.600	0.427±0.407	7.510±0.115	24.537±0.441	0.388±0.387
	BRITS (ref)	**3.080±0.069**	**6.577±0.108**	0.101±0.100	**3.116±0.082**	**7.095±0.126**	0.102±0.102
	CTA (ref)	7.101±0.109	18.595±0.382	0.424±0.468	6.573±0.130	18.280±0.386	0.392±0.455
	STGAE (ref)	7.861±0.103	13.951±0.142	0.390±0.135	5.892±0.100	35.975±0.471	0.452±0.439
ICDEP	LINPOL	19.836±0.176	10.462±0.103	0.283±0.138	**7.555±0.156**	12.539±0.128	**0.184±0.069**
	BILSTAE	**9.389±0.135**	**4.244±0.059**	**0.239±0.148**	8.166±0.156	13.040±0.119	0.281±0.132
	SAITS (ref)	14.941±0.145	6.446±0.064	0.449±0.200	9.718±0.169	8.201±0.077	0.328±0.157
	BRITS (ref)	10.583±0.123	4.550±0.052	0.268±0.138	7.563±0.151	**6.242±0.069**	0.249±0.118
	CTA (ref)	19.276±0.205	18.285±0.756	1.622±1.245	12.646±0.232	22.069±0.798	1.371±1.429
	STGAE (ref)	9.521±0.159	16.225±0.135	0.417±0.209	16.529±0.378	23.033±0.213	0.496±0.561

Table A.9. Performance metrics for reference baseline models (ref) on data with and without spatio-temporal patterns. Either data are following the MCAR mechanisms without spatio-temporal patterns, or data are induced with spatio-temporal pattern by applying the Markov Chain (MC) simulation model. Results are reported in % with mean±95%CI across time series, features, and validation folds. Best results (smallest metrics) are marked in bold.

Overall, results support findings from the main manuscript, indicating that the simulated missingness patterns from Markov chains pose more complex scenarios for DL imputation methods than simple MCAR inductions lacking in spatio-temporal structure. The improved performance of BRITS through jointly learning imputation as well as classification tasks suggests that downstream labels contain valuable auxiliary information for time series imputation. Similar claims have been made by benchmarking studies before [5,6]. However, treating these two tasks as independent problems, as reported in the main manuscript, has the advantage of freely selecting any downstream task. Also, since other reference models (SAITS, CTA, STGAE) do not have access to the labels, BRITS evaluations must be interpreted carefully. The BILSTAE trained on ICDEP could prevail over all reference models for the MCAR scenario. Here we showed that an autoencoder were successfully trained to model heterogenous multivariate time series imputations."

[1] Fan, Y., Yu, X., Wieser, R., Meakin, D., Shaton, A., Jaubert, J. N., ... & Wu, Y. (2023). Spatio-temporal denoising graph autoencoders with data augmentation for photovoltaic data imputation. Proceedings of the ACM on Management of Data, 1(1), 1-19.

[2] Cao, W., Wang, D., Li, J., Zhou, H., Li, L., & Li, Y. (2018). Brits: Bidirectional recurrent imputation for time series. Advances in neural information processing systems, 31.

[3] Du, W., Côté, D., & Liu, Y. (2023). Saits: Self-attention-based imputation for time series. *Expert Systems with Applications*, *219*, 119619.

[4] Wi, H., Shin, Y., & Park, N. (2024, March). Continuous-time autoencoders for regular and irregular time series imputation. In Proceedings of the 17th ACM International Conference on Web Search and Data Mining (pp. 826-835).

[5] Wang, X., Zhang, R., Sun, Y., & Qi, J. (2019, May). Doubly robust joint learning for recommendation on data missing not at random. In International Conference on Machine Learning (pp. 6638-6647). PMLR.

[6] Le Morvan, M., Josse, J., Scornet, E., & Varoquaux, G. (2021). What's a good imputation to predict with missing values?. Advances in Neural Information Processing Systems, 34, 11530-11540.

Linear interpolation is non-causal while LOCF is causal; mixing them obscures use-case distinctions (retrospective vs. online).
Suggestion: Report a strictly causal evaluation where only past information is allowed, and contrast it with an offline setting. Clarify which results correspond to which scenario.

RESPONSE #2.4: We thank the Reviewer#2 for calling for a deeper analysis regarding retrospective and online settings. In the **Results** section, we now write: "Imputation techniques were categorized into time-causal and non time-causal methods, with the latter having the advantage of accessing future data points (see Table 2). Time-causal methods are suitable for an online prediction setting whereas non time-causal methods offer increased performance for retrospective studies."

We also now distinctly present results for both use-cases separately with "Among non time-causal methods for retrospective studies, linear interpolation (LINPOL) achieved smallest local NRMSE across all datasets" and "Among time-causal methods which can be used for online settings, LOCF imputations performed best across all datasets"

The MNAR setup conditions on ground-truth value bins post-simulation, which may be a coarse proxy for clinical censoring.
Suggestion: Consider generative censoring rules tied to physiologic thresholds or workflow events (e.g., sensor removal under certain interventions), and include sensitivity analyses to these choices.

RESPONSE #2.5: We thank the reviewer for requesting additional justification for the amplified scenarios. In our opinion, physiological thresholds, censoring rules, and clinical interventions are of great interest with respect to the occurence of missingness patterns. In the **Additional Discussion A.2**, we now describe these aspects in detail by adding the following:

"When investigating existing effects between covariates and missingness, a general trend of increased missingness was observed among elderly patients in ICDEP. Several studies indicate that low peripheral perfusion, which is more common in older adults, can lead to higher rates of missing values in pulse oximetry signals [1]. Analyses also confirmed that low values of Spo2 < 70%, common in elderly patients, can lead to sensor malfunction and ultimately to data loss [2]. As findings from [3,4] suggest, the post-hoc simulation of MNAR effects bases on these assumptions that less values are available for more extreme levels of clinical parameters. Hence, negative and positive MNAR effects were amplified with no change in general results, while the main manuscript reports imputation results where more unobserved values are present for lower value bins. In MIMIC and HIRID, missing values appeared more frequently in younger intensive care patients. A multi-center study [5] found that older adults (≥ 75 years) were less likely to receive certain interventions (e.g., imaging or drainage) compared to younger patients. Similarly, younger patients (≤ 65 years) made up the largest proportion in a study cohort investigating ventilated patients [6].

Either way, missingness patterns in clinical time series often have multi-factorial reasons which are not fully clear. All three datasets MIMIC, HIRID, and ICDEP were also restricted to a selection of routinely collected EHRs, not providing the full cascade of actions resulting in sensor removal or medical interventions (complex inherent MNAR effects). As a limitation, we did not investigate transition probabilities conditional on workflow events. It is plausible that certain procedures affect missingness, but we did not have fully access to these data or procedures (e.g. medical codes for surgical interventions) are documented after the hospital stay, discarding temporal dependencies. Real-world clinical missingness patterns might also depend on monitoring guidelines, the structure of the health-care system, staffing levels, hospital routines, as well as additional patient related factors such as diagnosis, disease progression, and comorbidities [7]. Due to the selection of clinical data from Germany, the US, and Switzerland, detected missingness patterns are also limited to health systems in these countries."

[1] Poorzargar, K., Pham, C., Ariaratnam, J., Lee, K., Parotto, M., Englesakis, M., ... & Nagappa, M. (2022). Accuracy of pulse oximeters in measuring oxygen saturation in patients with poor peripheral perfusion: a systematic review. Journal of clinical monitoring and computing, 36(4), 961-973.
[2] Elgendi, M., Fletcher, R. R., Tomar, H., Allen, J., Ward, R., & Menon, C. (2021). The striking need for age diverse pulse oximeter databases. Frontiers in Medicine, 8, 782422.
[3] Haliduola, H. N., Bretz, F., & Mansmann, U. (2022). Missing data imputation using utility-based regression and sampling approaches. Computer Methods and Programs in Biomedicine, 226, 107172.
[4] Sondhi, A., Weberpals, J., Yerram, P., Jiang, C., Taylor, M., Samant, M., & Cherng, S. (2023). A systematic approach towards missing lab data in electronic health records: A case study in non-small cell lung cancer and multiple myeloma. CPT: Pharmacometrics & Systems Pharmacology, 12(9)
[5] EUROBACT-2 Study Group, & European Society of Intensive Care Medicine. (2024). Presentation, management, and outcomes of older compared to younger adults with hospital-acquired bloodstream infections in the intensive care unit: a multicenter cohort study. Infection.
[6] Ma, J. G., Zhu, B., Jiang, L., Jiang, Q., & Xi, X. M. (2022). Gender-and age-based differences in outcomes of mechanically ventilated ICU patients: a Chinese multicentre retrospective study. BMC anesthesiology, 22(1), 18.
[7] Ayilara, O. F., Zhang, L., Sajobi, T. T., Sawatzky, R., Bohm, E., & Lix, L. M. (2019). Impact of missing data on bias and precision when estimating change in patient-reported outcomes from a clinical registry. Health and quality of life outcomes, 17(1), 106.

Do your main conclusions hold when restricting evaluation to causal imputers (no access to the future)? How do rankings change relative to the offline setting?

RESPONSE #2.6: We thank reviewer#2 for requesting information about ranks for imputation methods when restricting the input space to past observed values only. Based on this comment, we enhanced the **Results** section by the following sentences:

"While LINPOL achieved the overall lowest local RMSE scores across all datasets for methods that can access future points in time (10.578±0.103, 17.824±0.296, 2.539±0.128 for MIMIC, HIRID, ICDEP), different rankings were observed for time-causal imputers. In MIMIC, the DL model LSTAE yielded a RMSE of 11.775±0.105, the running average method RMEAN was followed by LOCF with scores of 11.798±0.104 and 11.862±0.114, respectively. In contrast, linear LOCF imputations (RMSE of 17.280±0.242) as well as RMEAN (RMSE of 19.542±0.233) and RMEDI (RMSE of 20.406±0.256) methods were better suited for HIRID than DL imputers. In ICDEP, the methods LSTAE, LOCF and RMEDI yielded the lowest RMSE of 13.877±0.122, 14.247±0.139, and 14.815±0.136. Note that we highly suggest considering additional novel performance scores (e.g., CCorD, NWSD) to fully assess imputation quality of benchmarked methods."

Overall, a good and timely paper with clear practical relevance.

RESPONSE #2.7: We thank the reviewer for all remarks that led to an improved manuscript.

(Remarks on code availability)

The codebase is present and aligned with the paper, and the README documents dataset sources and high-level model information.

Reviewer #3 (Remarks to the Author):

Summary

This manuscript introduces a benchmark framework for evaluating time-series imputation methods in clinical datasets. The authors propose a simulation model that induces realistic spatio-temporal missingness patterns using Markov chains derived from empirical transition probabilities. They apply 20 imputation techniques—including simple statistical methods, MICE variants, and deep spatio-temporal autoencoders—and assess both reconstruction accuracy and downstream prediction performance across three datasets (MIMIC, HIRID, ICDEP). The experimental results indicate that deep learning methods outperform traditional approaches under MCAR conditions, whereas classical methods achieve superior performance under MAR and MNAR scenarios. The study addresses an important gap in the literature, namely the lack of benchmarks that reflect structured missingness in clinical time series.

Strong Points：

1. Innovative Spatio-Temporal Missingness Simulation Model: For the first time, Markov chain sampling is adopted to generate spatio-temporal missing values that are close to real clinical scenarios, breaking the limitation of previous studies focusing mostly on MCAR patterns. It accurately restores the missing correlations between features and over time in clinical data, providing a reliable basis for real-scenario testing of imputation methods.
2. Comprehensive Evaluation System for Imputation Methods: A total of 20 imputation methods are systematically included, covering four categories: mean/median, interpolation/extrapolation, multiple imputation by chained equations, and deep learning. It includes both classic simple methods and cutting-edge spatio-temporal autoencoders, with a comprehensive comparison dimension, offering abundant references for method selection in different scenarios.
3. Support from Real and Diverse Clinical Datasets: Three real clinical datasets with different sources and complementary scenarios are adopted, covering routine ICU monitoring and intraoperative monitoring scenarios. The data scale is large and features are standardized, improving the external validity and generalizability of the research conclusions.

However, the paper exhibits several non-negligible limitations: the clinical rationality of some simulated missing states is unvalidated, the hyperparameter tuning strategy lacks sufficient justification, the amplification factors for MAR/MNAR are subjectively set without clinical basis, the evaluation metrics lack clinical significance alignment, and the generalizability is constrained by the Western-centric datasets and limited feature scope. These shortcomings weaken the robustness and persuasiveness of the conclusions.

RESPONSE #3.1: We thank Reviewer#3 for the positive feedback regarding the novelty of our methodology. The current manuscript now includes clinical justification of observed missingness patterns, provides technical insights into our hyperparameter tuning strategy as well as amplification factors, and critically discusses the limitations and scope of our results. Besides we agree that our data is limited to the Western-centric health systems and we acknowledge: “Due to the selection of clinical data from Germany, the USA, and Switzerland, detected missingness patterns are also limited to health systems in these countries. We are open for collaborations across other globally distributed medical centers to additionally validate our findings” in the **Additional Discussion A.2**.

Weak Points

1. Rationality of Missingness Simulation States: The paper defines 32 spatio-temporal missing states but fails to verify whether all states have real clinical significance. The clinical relevance of some low-frequency states remains unclear, potentially disconnecting simulated scenarios from actual clinical data.

RESPONSE #3.2: We thank Reviewer#2 for requesting additional justification for 32 spatio-temporal missing states. In the enhanced **Additional Discussion A.3**, we relate detected states to clinical practice with

"We observed missing states where respiratory rate is abundantly unavailable (s = [0,0,0,0,1]), commonly found in the ICU dataset HIRID (f = 0.23). For patients in ICUs, who are mechanically ventilated, the configured respiratory rate is measured rather than the spontaneous breaths per minute [1]. These configurations do not change frequently, so clinical information systems store discontinuous mechanically configured respiratory rates, expressing data gaps that are often present in HIRID. The simultaneous absence of systolic and diastolic blood pressure (BP) values (s = [0,1,1,0,0]), primary observed in ICDEP (f = 0.17), is caused by measuring these two variables with the same sensor [2]. Since we dealt with a narrow equidistance time grid in ICDEP of 5 min intervals, more data gaps occurred (e.g., due to the BP cuff being inflated every 15 min) than in other ICU data sets. Spo2 values are found to be uniquely missing (s = [0,0,0,1,0]) with lower occurrence frequencies (e.g., f = 0.036 in MIMIC) than previous described missing patterns. Pulse oximetry is used for Spo2 measurements by attaching a clip to a patient's finger, these clips are oftentimes accidently detached or patients remove the sensor in agitated states [3,4]. Additional missingness patterns that are seldomly present in the extracted data (e.g., s = [1,0,1,0,1] with f = 4.6E-6 in MIMIC) could have occurred through monitoring transition issues, system errors, or fragmented extractions [5]. Hence, multiple authors call for primary source systems that are error robust, interoperable, and designed for secondary data analysis [6]. Besides, patient treatment in a bustling hospital environment also hampers continuous monitoring procedures that might have led to some rarely observed missing states [7]."

[1] Esteban, A., Anzueto, A., Alia, I., Gordo, F., Apezteguia, C., Palizas, F., ... & Tobin, M. J. (2000). How is mechanical ventilation employed in the intensive care unit? An international utilization review. American journal of respiratory and critical care medicine, 161(5), 1450-1458.
[2] Gibson, L. E., Henriques, T. S., Costa, M. D., Davis, R. B., Mittleman, M. A., Mathur, P., & Subramaniam, B. (2020). Comparison of invasive and noninvasive blood pressure measurements for assessing signal complexity and surgical risk in cardiac surgical patients. Anesthesia & Analgesia, 130(6), 1653-1660.
[3] Bowes Jr III, W. A., Corke, B. C., & Hulka, J. (1989). Pulse oximetry: a review of the theory, accuracy, and clinical applications. Obstetrics & Gynecology, 74(3 Part 2), 541-546.
[4] Nguyen, H., Jang, S., Ivanov, R., Bonafide, C. P., Weimer, J., & Lee, I. (2018). Reducing pulse oximetry false alarms without missing life-threatening events. Smart Health, 9, 287-296.
[5] Kramer, O., Even, A., Matot, I., Steinberg, Y., & Bitan, Y. (2021). The impact of data quality defects on clinical decision-making in the intensive care unit. Computer Methods and Programs in Biomedicine, 209, 106359.
[6] Vorisek, C. N., Klopfenstein, S. A. I., Löbe, M., Schmidt, C. O., Mayer, P. J., Golebiewski, M., & Thun, S. (2024). Towards an interoperability landscape for a national research data infrastructure for personal health data. Scientific Data, 11(1), 772.
[7] Stoker, M. R. (2008). Common errors in clinical measurement. Anaesthesia & Intensive Care Medicine, 9(12), 553-558.

2. Sufficiency of Hyperparameter Tuning for Imputation Methods: Grid search and Hyperband search are used for hyperparameter tuning of autoencoders, but the rationale for designing the search space is not explained. Additionally, the impact of different tuning strategies on model performance is not compared, which may result in suboptimal parameters being overlooked.

RESPONSE #3.3: In **Methods**, we now explain how we designed the hyperparameter search space:

"We chose the number of nodes per layer for the simple stacked autoencoder SAE in a monotonically decreasing manner, halving the number of nodes for each subsequent layer (e.g., {32, 16, 8, 4}), as it is common for most DL architectures [1]. We provided three different sets of such node stackings to test

small, medium, and high capacity models that compress inputs to a bottleneck dimension on the last layer. Hereby, the model focuses on layer-wise compressed inputs, avoiding overfitting [2]. The authors Gondara et al. who originally proposed the MIDA architecture suggested a hyperparameter Θ of 7 and varying it around this value [3]. Consequently, we tested different configurations of Θ ({4, 6, 9}) and configured a lower dimensionality for shorter multivariate time series from MIMIC and a higher dimension for longer sequences in HIRID. Inspired by Li et al. [4], a vanilla STAE model was designed that use one or two stacked LSTM or GRU modules. For these modules, four different number of hidden states ({32, 64, 128, 256}) were integrated into the search space, as this selection is commonly chosen for previously proposed autoencoder architectures [5, 6]. Dropout and weighted decay, as implementing a L_2-norm, were implemented as regularization mechanisms, actively reducing the chances that models simply learn samples instead of generalizable trends. By applying Hyperband, we drastically reduced the computational complexity and time for training multiple model configurations in parallel. However, we are aware that fully exhaustive search, such as Grid search, could have led to different parameter selections which would have required too many training resources for our experiments. Following established literature, we fitted autoencoder models to real-world data, utilizing empirical heuristics to determine optimal hyperparameter configurations."

We further describe the computational time that was required for training and hyperparameter tuning in **RESPONSE #3.11** to estimate the computational resources required for training.

[1] Alqahtani, A., Xie, X., & Jones, M. W. (2021, November). Literature review of deep network compression. In *Informatics* (Vol. 8, No. 4, p. 77). MDPI.
[2] Sun, L., Wu, C., Ching, W. K., & Akutsu, T. (2025). On the Compressive Power of Autoencoders With Linear and ReLU Activation Functions. *Neural Computation*, *37*(2), 235-259.
[3] Gondara, L., & Wang, K. (2018, June). Mida: Multiple imputation using denoising autoencoders. In *Pacific-Asia conference on knowledge discovery and data mining* (pp. 260-272). Cham: Springer International Publishing.
[4] Li, D., Li, L., Li, X., Ke, Z., & Hu, Q. (2020). Smoothed LSTM-AE: A spatio-temporal deep model for multiple time-series missing imputation. Neurocomputing, 411, 351-363.
[5] Ghimire, S., Deo, R. C., Wang, H., Al-Musaylh, M. S., Casillas-Pérez, D., & Salcedo-Sanz, S. (2022). Stacked LSTM sequence-to-sequence autoencoder with feature selection for daily solar radiation prediction: A review and new modeling results. *Energies*, *15*(3), 1061.
[6] Qais, M. H., Kewat, S., Loo, K. H., Lai, C. M., & Leung, A. (2023). LSTM-based stacked autoencoders for early anomaly detection in induction heating systems. *Mathematics*, *11*(15), 3319.

3. Limitations of Clinical Relevance in Downstream Tasks: The downstream tasks only include in-hospital mortality and postoperative delirium, excluding more refined clinical outcomes. It is difficult to verify the impact of imputation methods on complex clinical decisions, and the effect of imputation errors on clinical risk stratification thresholds is not analyzed.

RESPONSE #3.3: We added the following thresholding analysis and limitations to our **Discussion**:

"While in-hospital death is less clinically valuable than more actionable outcomes (e.g., hypotension), this endpoint in MIMIC and HIRID is technically seen as an adequate downstream task, since the prediction model inputs were restricted to five vital signs to solve these tasks. Related literature suggests that patient death can serve as an effective auxiliary target variable for evaluating new clinical prediction or imputation models [1,2]. While we acknowledge that utilizing diverse labels would enhance the impact of downstream analysis, the limited availability of clearly defined endpoints in real-world databases like MIMIC and HIRID remains a significant impediment. For the ICDEP dataset, POD is reflecting a clinically actionable and valuable endpoint [3].

When considering the overall best performing downstream model, LSTAE imputations resulted in an AUROC of 0.710±0.074 and an AUPRC of 0.213±0.096 in HIRID on simulated data without any further

MAR or MNAR amplifications. For a fixed threshold where the Youden index was maximized [4], this model achieved a maximum sensitivity of 0.65 and a specificity of 0.60 with a corresponding 0.18 precision. Assuming 100 cases of which 10 are associated with a lethal outcome. Here, 7 of 10 patients at risk (true positives) would be correctly identified, 54 patients that are not at risk (false positives) would be correctly labeled as such whereas 36 cases would be falsely predicted at risk (false positives). This simple numerical example shows that setting clinical thresholds is a non-trivial task where a low prevalence rate and low precision result in overtreatment, expressed by a high number of false positives."

[1] Harutyunyan, H., Khachatrian, H., Kale, D. C., Ver Steeg, G., & Galstyan, A. (2019). Multitask learning and benchmarking with clinical time series data. *Scientific data*, *6*(1), 96.
[2] Yao, F., Luo, J., Zhou, Q., Wang, L., & He, Z. (2025). Development and validation of a machine learning-based prediction model for hepatorenal syndrome in liver cirrhosis patients using MIMIC-IV and eICU databases. *Scientific Reports*, *15*(1), 2743.
[3] Giesa, N., Sekutowicz, M., Rubarth, K., Spies, C. D., Balzer, F., Haufe, S., & Boie, S. D. (2024). Applying a transformer architecture to intraoperative temporal dynamics improves the prediction of postoperative delirium. Communications Medicine, 4(1), 251.
[4] Fluss, R., Faraggi, D., & Reiser, B. (2005). Estimation of the Youden Index and its associated cutoff point. Biometrical Journal: Journal of Mathematical Methods in Biosciences, 47(4), 458-472.

4. Subjectivity of Amplification Factors for MAR/MNAR: In the MAR and MNAR scenarios, after applying amplification to the missing data, does the resulting missingness pattern still correspond to realistic clinical settings? How is it ensured that the amplification is not excessive to the extent that too much original information is removed? If such amplification is excessive, and the dataset without amplification more closely reflects real-world conditions, then deep learning methods would, in fact, remain the more effective approach. In addition, the setting of age amplification factors for MAR amplification and feature value amplification factors for MNAR amplification lacks clinical basis. The association between factor selection and real clinical missing mechanisms is not explained, and the sensitivity of results to different factor combinations is not verified.

RESPONSE #3.4: We thank the Reviewer #3 for asking about changes in occurrences of states that reflect missingness patterns across MAR/MNAR scenarios. We included **Figure A.4** (see below) in **Supplementary A** that displays differences in these state occurrences across age-bins for MAR as well as value-bins for MNAR between original and simulated data. Overall, our results indicate that DL autoencoders clearly outperform linear baselines only if complex spatio-temporal patterns are not simulated. For all amplification scenarios, we believe that linear imputers yield robust imputation performances compared to DL methods without requiring training. We further discuss the following in **Additional Discussion A.1**:

"**Figure A.4** quantifies changes in occurrence probabilities before and after MAR (panel **a**) or MNAR (panel **b**) amplifications across either age-bins or value-bins, respectively. For the amplification of MAR effects, we changed the proposed sampling procedure so that proportions of probabilities for the total present state (TPS = [0,0,0,0,0]) are shifted to the total absent state (TAS = [1,1,1,1,1]) to induce missingness, conditioned on a patient's age. As expected, **Figure A.4a** reflect this behavior (e.g., probabilities of 0.22, 0.56, 0.83 for TSP across three age-bins in MIMIC) without major changes in the order of more complex missing states (e.g., probability of 0.024±0.005 for the original and simulated state s = [0,1,1,0,0]). Due to shifts in overall availability probabilities, minor changes in seldom states occurred for MAR amplifications.

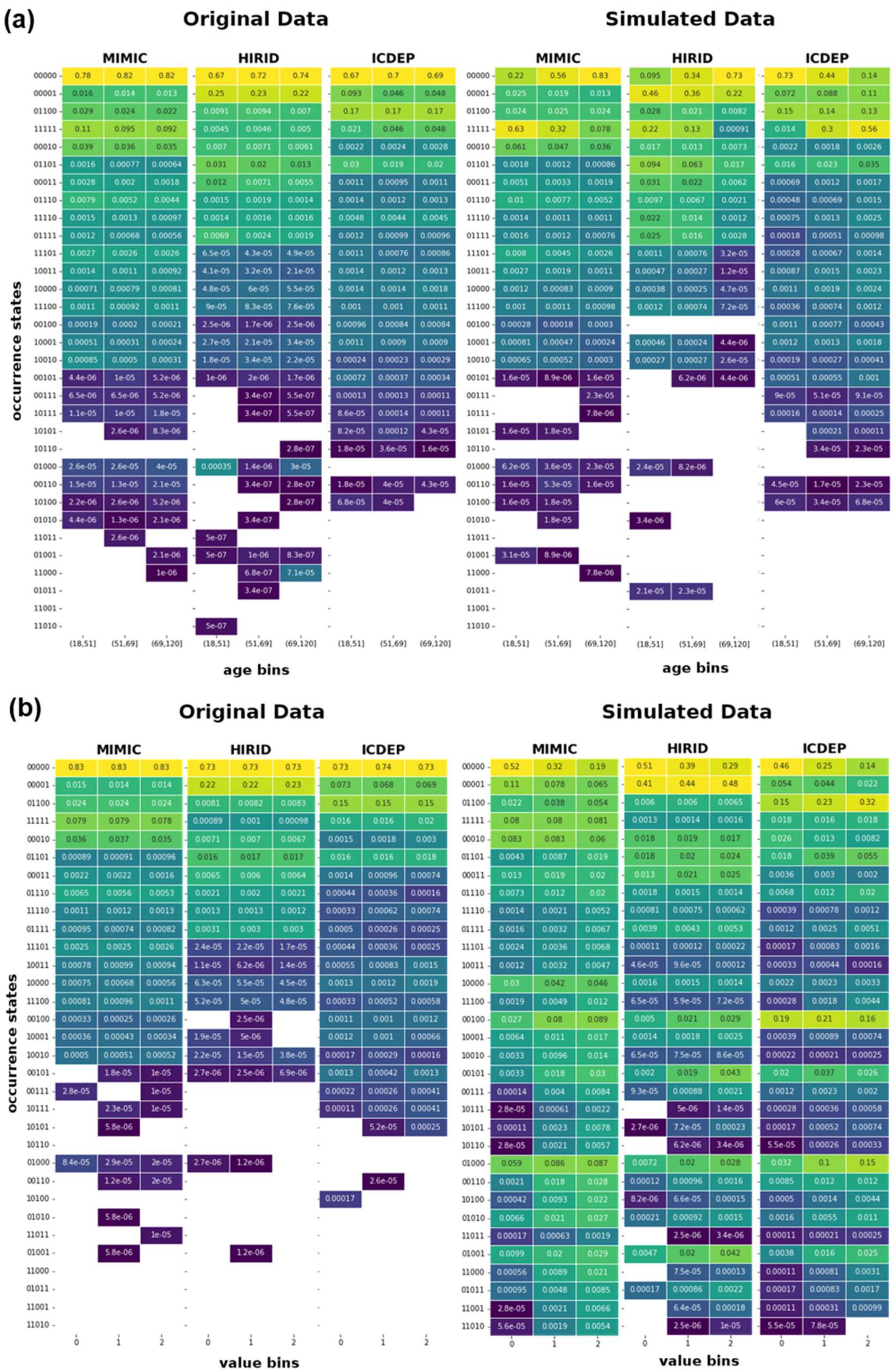

Figure A.4: Occurrence probabilities before and after missing at random (a) or missing not at random (b) amplification. Occurrence states were either conditioned on equally sized age bins (panel **a**) to amplify the missing at random (MAR) effect, or on ground truth (GT) value bins (panel **b**) to amplify a missing not at random (MNAR) effect.

Figure A.4b shows that after simulating spatio-temporal missingness patterns on CCD, no ad-hoc MNAR was detected since the resampling procedure was not conditioned on feature value levels. After

amplification of these effects, seldom states became more frequent in the simulated data. The sampling procedure was adapted so that after simulating original spatio-temporal missingness, feature values were removed according to missingness rates that depend on the GT value level. Hence, overall states where the individual feature was encoded as absent became more likely depending on the underlying true GT value distributions. While the main manuscript includes results from a negative MNAR effect (higher missingness for lower values), ablations with a positive effect direction led to the same benchmarking findings. Overall, results across all amplification scenarios suggest that DL imputers excel over linear models primarily in the simplistic unrealistic MCAR case where spatio-temporal pattens are not present, a method that has been used by previous benchmarking studies."

We provide detailed clinical justifications for MAR and MNAR conditions, also explaining the configuration of amplification factors, in **RESPONSE #3.7**

"When investigating existing effects between covariates and missingness, a general trend of increased missingness was observed among elderly patients in ICDEP. Several studies indicate that low peripheral perfusion, which is more common in older adults, can lead to higher rates of missing values in pulse oximetry signals [1]. Analyses also confirmed that low values of Spo2 < 70%, common in elderly patients, can lead to sensor malfunction and ultimately to data loss [2]. As findings from [3,4] suggest, the post-hoc simulation of MNAR effects bases on these assumptions that less values are available for more extreme levels of clinical parameters. Hence, negative and positive MNAR effects were amplified with no change in general results, while the main manuscript reports imputation results where more unobserved values are present for lower value bins. In MIMIC and HIRID, missing values appeared more frequently in younger intensive care patients. A multi-center study [5] found that older adults (≥ 75 years) were less likely to receive certain interventions (e.g., imaging or drainage) compared to younger patients. Similarly, younger patients (≤ 65 years) made up the largest proportion in a study cohort investigating ventilated patients [6].

Either way, missingness patterns in clinical time series often have multi-factorial reasons which are impossible to tease out retrospectively. Especially MNAR effects cannot be measured in real-world settings due to the reason for a value being missing is never measured. All three datasets MIMIC, HIRID, and ICDEP were also restricted to a selection of routinely collected EHRs, not providing the full cascade of actions resulting in sensor removal or medical interventions (complex inherent MNAR effects). Real-world clinical missingness patterns also depend on monitoring guidelines, the structure of the health-care system, staffing levels, hospital routines, as well as additional patient related factors such as diagnosis, disease progression, and comorbidities [7]. Amplification factors were set so that the altered probabilities for a highly missing state did not exceed 90%, the probabilities for more available states deepening on MAR or MNAR conditions were changed proportionally to have pronounced effects in a controlled environment."

[1] Poorzargar, K., Pham, C., Ariaratnam, J., Lee, K., Parotto, M., Englesakis, M., ... & Nagappa, M. (2022). Accuracy of pulse oximeters in measuring oxygen saturation in patients with poor peripheral perfusion: a systematic review. Journal of clinical monitoring and computing, 36(4), 961-973.
[2] Elgendi, M., Fletcher, R. R., Tomar, H., Allen, J., Ward, R., & Menon, C. (2021). The striking need for age diverse pulse oximeter databases. Frontiers in Medicine, 8, 782422.
[3] Haliduola, H. N., Bretz, F., & Mansmann, U. (2022). Missing data imputation using utility-based regression and sampling approaches. Computer Methods and Programs in Biomedicine, 226, 107172.
[4] Sondhi, A., Weberpals, J., Yerram, P., Jiang, C., Taylor, M., Samant, M., & Cherng, S. (2023). A systematic approach towards missing lab data in electronic health records: A case study in non-small cell lung cancer and multiple myeloma. CPT: Pharmacometrics & Systems Pharmacology, 12(9)
[5] EUROBACT-2 Study Group, & European Society of Intensive Care Medicine. (2024). Presentation, management, and outcomes of older compared to younger adults with hospital-acquired bloodstream infections in the intensive care unit: a multicenter cohort study. Infection.

[6] Ma, J. G., Zhu, B., Jiang, L., Jiang, Q., & Xi, X. M. (2022). Gender-and age-based differences in outcomes of mechanically ventilated ICU patients: a Chinese multicentre retrospective study. BMC anesthesiology, 22(1), 18.
[7] Ayilara, O. F., Zhang, L., Sajobi, T. T., Sawatzky, R., Bohm, E., & Lix, L. M. (2019). Impact of missing data on bias and precision when estimating change in patient-reported outcomes from a clinical registry. Health and quality of life outcomes, 17(1), 106.

5. Lack of Clinical Significance in Evaluation Metrics: Existing evaluation metrics focus on statistical errors but do not design indicators combined with actual clinical significance, making it impossible to reflect the applicability of imputation results to clinical practice.

RESPONSE #3.5: We agree with Reviewer #3 that evaluation metrics lack in further explaining their clinical relevance. We added some clinical explanations of these metrics in the **Methods**:

“The RMSE, widely used in medical research studies [1,2], describes the average clinical error magnitude that is directly interpretable for clinicians per single feature to reflect the acceptable range of imputation errors (e.g., RMSE of 500 mmHg for bp_sys). To make RMSE terms comparable across features, a normalization term was applied, so that the overall reported error metrics reflect the average deviation of imputed values from GT values across all five included parameters. ACorE and CCorE quantify the correlation of single time series values with temporally shifted versions, detecting changes in temporal dynamics for one single time series and across multiple ones [3]. Hereby, the temporal change between clinical parameters can be described per single time step (e.g., values of bp_sys depend 90% on its past values). Instead of quantifying pairwise errors such as the RMSE, the WSD [4] measures the shifts in variance and skewness between distributions. Differences in distributions capture the preservation of clinical characteristics such as outliers and overall mean tendencies across the entire patient population, making them highly useful for descriptively analyzing the patient cohort [5].“

		MAE			RMSE		
**feature**	**method**	**MIMIC**	**HIRID**	**ICDEP**	**MIMIC**	**HIRID**	**ICDEP**
bp_dia	1locf	7.93±0.21	4.03±0.12	9.85±0.23	11.52±0.37	7.77±0.16	13.81±0.29
	bilstaestat	7.15±0.32	5.58±0.82	8.75±0.38	9.91±0.52	8.23±0.6	11.74±0.59
	linpol	6.92±0.28	3.12±0.11	8.53±0.2	10.18±0.48	6.64±0.54	12.11±0.33
	lstae	7.93±0.16	5.29±0.19	9.46±0.18	10.85±0.29	8.07±0.19	12.5±0.21
bp_sys	1locf	12.09±0.11	7.24±0.2	14.44±0.31	16.63±0.24	11.41±0.24	20.14±0.36
	bilstaestat	11.15±0.05	10.96±1.55	12.71±0.81	14.85±0.15	14.78±1.42	17.37±0.83
	linpol	10.49±0.16	5.35±0.2	12.38±0.3	14.75±0.35	8.99±0.44	17.71±0.34
	lstae	12.53±0.19	10.84±0.52	13.58±0.42	16.56±0.41	14.81±0.35	18.27±0.42
hr	1locf	6.86±0.12	2.44±0.2	10.57±0.37	10.25±0.17	5.48±1.19	13.76±0.43
	bilstaestat	7.44±0.19	6.26±0.7	8.92±0.48	10.22±0.14	9.46±0.92	11.95±0.9
	linpol	5.9±0.1	1.82±0.19	9.46±0.29	9.18±0.17	4.64±1.23	12.9±0.55
	lstae	9.07±0.1	6.34±0.23	9.98±0.29	12.25±0.15	9.71±0.42	13.2±0.51
rr	1locf	3.6±0.05	9.31±1.03	2.22±0.05	5.01±0.04	30.51±3.9	3.55±0.55
	bilstaestat	3.12±0.03	13.25±1.15	1.83±0.03	4.19±0.06	34.72±2.56	2.98±0.37
	linpol	3.23±0.03	9.27±1.01	1.9±0.07	4.49±0.02	28.45±3.67	3.21±0.43
	lstae	3.43±0.11	12.62±1.96	1.86±0.05	4.57±0.14	32.82±5.51	3.01±0.4
spo2	1locf	1.68±0.06	0.83±0.01	0.86±0.02	2.97±0.28	1.69±0.16	1.38±0.04
	bilstaestat	1.55±0.02	1.38±0.09	0.84±0.03	2.6±0.13	2.28±0.24	1.29±0.01
	linpol	1.53±0.06	0.66±0.01	0.86±0.04	2.7±0.22	1.48±0.13	1.34±0.04
	lstae	1.72±0.05	1.38±0.07	0.96±0.02	2.74±0.12	2.25±0.19	1.4±0.03

Table A.10. Mean absolute error (MAE) and root-mean squared error (RMSE) per feature. For each feature, the local MAE and RMSE is displayed as mean±standard-deviation grouped by the imputation method and per dataset (MIMIC, HIRID, ICDEP). The reported summary statistics are calculated across all patient time series and across all corss-validation folds

To expand our clinical analysis, we also added the MAE and RMSE without normalization in the Supplementary A and grouped these errors by feature and datasets. We added the following aspects to the **Discussion**:

“When assessing MAE and RMSE grouped by feature without normalization, systolic blood pressure (bp_sys) consistently yields the highest errors (max. of 13.58±0.42 MAE and 18.27±0.42 RMSE) (see Table A.10 in Supplementary A). Elevated errors for bp_sys occur likely due to high variability and wider physiological range of blood pressure readings compared to more stable vitals (standard-deviation of max. 29.99 mmHg in CCD). Conversely, blood oxygen (spo2) exhibits the lowest errors (min. of 0.66±0.01 MAE and 1.34±0.04 RMSE), reflecting its narrower clinical range where even small deviations can be physiologically relevant. When comparing metrics, MAE values are notably higher than RMSE across all features, expressing a large deviation for respiratory rate (rr) recordings in HIRID (9.27±1.01 MAE, 28.45±3.67 RMSE for LINPOL). These deviations confirm increased sensitivity to large outliers for high-frequency vital signs which, in a clinical context, often are caused by critical acute changes or sensor noise.”

1. Toye, A. A., Celik, A., & Kleinberg, S. (2025). Benchmarking Missing Data Imputation Methods for Time Series Using Real-World Test Cases. Proceedings of machine learning research, 287, 480.
2. Luo, Y. (2022). Evaluating the state of the art in missing data imputation for clinical data. Briefings in Bioinformatics, 23(1)
3. Taylor, D. (1990). Time-series analysis: Use of autocorrelation as an analytic strategy for describing pattern and change. Western Journal of Nursing Research, 12(2), 254-261.
4. Panaretos, V. M., & Zemel, Y. (2019). Statistical aspects of Wasserstein distances. Annual review of statistics and its application, 6(1), 405-431.
5. Kazdaghli, S., Kerenidis, I., Kieckbusch, J., & Teare, P. (2024). Improved clinical data imputation via classical and quantum determinantal point processes. Elife, 12, RP89947.

Below are some detailed comments.
1. The first-order Markov assumption for missingness transitions may oversimplify real-world dependencies. Clinical data often exhibit multi-step or periodic patterns of missingness. A brief sensitivity analysis or discussion of this limitation would strengthen the methodological rigor.

RESPONSE #3.5: We enhanced our **Discussion** by the following aspects:

“Missingness in clinical time series can follow complex periodic patterns. The proposed Markov chain simulation method is intended to approximate the observed missingness structure, rather than to explain the underlying data generating process of clinical variables or unobserved patterns. A higher-order Markov process would increase the model’s flexibility to learn oscillations and distinct missingness patterns more freely. Evaluating the order of such process is beyond the scope of this study. However, Figure 2 clearly demonstrates that auto- and cross-correlation patterns found in the original data could also be reproduced in the simulated data that exhibit spatio-temporal missingness patterns.“

2. Selection of imputation methods. The survey of deep learning–based approaches appears insufficient. All deep learning methods in this study are based on the denoising autoencoder (DAE) architecture. There are, however, other relevant architectures for imputation, such as transformer-based [1], diffusion-based [2], and CNN-based [3] models, which have been applied to datasets like Physionet [4]. Please clarify the rationale for not including these alternative deep learning approaches in this work.

RESPONSE #3.6: We thank the Reviewer#2 for this comment. As also Reviewer#1 suggested that additional baseline DL imputers should be evaluated, we introduce suggested reference models in the **Introduction** section with:

“Fan et al. [1] proposed a CNN autoencoder architecture for processing time series via windowing, which involved a trainable graph for static attributes. Their proposed model was denoted as STGAE (Spatio-temporal Denoising Graph Autoencoder). The authors Cao et al. [2] proposed the model architecture BRITS (Bidirectional Recurrent Imputation for Time Series) which jointly learn imputations for multivariate time series as well as a downstream classification task. For the imputation

of elements in temporal data, known values are masked and recurrently predicted by an LSTM cell. Similarly, the authors Du et al. [3] use masking of known values in time series that hold observed as well as unobserved data. Their model architecture SAITS (Self-Attention-based Imputation for Time Series) relies on self-attention modules instead of recurrent mechanisms. In 2018, Chen et al. [4] introduced the concept of neural ordinary differential equations (ODEs) that can be used to continuously model a latent space, improving discrete RNN representations by integration capabilities such as Hamiltonian integrators. The CTA (continuous-time autoencoder) model by Wi et al. exploits ODEs that are applied on regression coefficients from cubic splines, fitted to multivariate time series."

Apart from these suggested architectures, we configured LSTAE and BILSTAE model as described in the main manuscript as newly implemented methods that showed reliable imputation results. Besides, we describe the training procedure and evaluation results for additional reference models in the **Supplement A** with:

"Due to the configuration of LSTM modules in BRITS, this model was trained with batch size of one and accumulating gradients, similar to the STAE models reported in the main manuscript. STGAE and SAITS were trained on time series that were padded to the longest sequence within one batch. CTA was adopted so that the model could also handle time series of unequal lengths. To inherently learn to impute time series via masking, 10% of the actual observed values inside the training set were masked out. Hereby, no LOCF pre-imputation was required for learning while only the imputed values contributed to the MSE loss. All reference model architectures were trained with published hyperparameter configurations [1-4]."

		A) MCAR induction without spatio-temporal missingness patterns			B) Markov chain simulation of spatio-temporal missingness patterns		
Data	Method	CCorD_loc	NRMSE_loc	NWSD_glob	CCorD_loc	NRMSE_loc	NWSD_glob
MIMIC	LINPOL	14.024±0.172	3.136±0.060	**0.140±0.038**	6.581±0.164	10.578±0.103	**0.090±0.016**
	BILSTAE	10.143±0.131	**1.947±0.046**	0.248±0.043	6.581±0.159	10.840±0.097	0.234±0.041
	SAITS (ref)	13.954±0.145	5.394±0.053	0.489±0.096	10.002±0.196	6.399±0.060	0.424±0.084
	BRITS (ref)	**9.067±0.125**	3.003±0.033	0.169±0.030	**6.220±0.156**	**3.926±0.041**	0.172±0.029
	CTA (ref)	13.875±0.178	5.205±0.065	0.623±0.157	9.916±0.245	6.369±0.077	0.532±0.135
	STGAE (ref)	9.125±0.159	6.225±0.135	0.417±0.209	13.730±0.256	24.327±0.202	0.554±0.160
HIRID	LINPOL	4.524±0.095	3.337±0.183	**0.043±0.040**	3.384±0.090	17.824±0.296	**0.026±0.024**
	BILSTAE	3.729±0.074	5.694±0.138	0.152±0.138	3.348±0.091	18.685±0.364	0.337±0.385
	SAITS (ref)	8.887±0.100	1.965±0.600	0.427±0.407	7.510±0.115	24.537±0.441	0.388±0.387
	BRITS (ref)	**3.080±0.069**	**5.577±0.108**	0.101±0.100	**3.116±0.082**	**7.095±0.126**	0.102±0.102
	CTA (ref)	7.101±0.109	8.595±0.382	0.424±0.468	6.573±0.130	18.280±0.386	0.392±0.455
	STGAE (ref)	7.861±0.103	3.951±0.142	0.390±0.135	5.892±0.100	35.975±0.471	0.452±0.439
ICDEP	LINPOL	19.836±0.176	0.462±0.103	0.283±0.138	**7.555±0.156**	12.539±0.128	**0.184±0.069**
	BILSTAE	**9.389±0.135**	**4.244±0.059**	**0.239±0.148**	8.166±0.156	13.040±0.119	0.281±0.132
	SAITS (ref)	14.941±0.145	5.446±0.064	0.449±0.200	9.718±0.169	8.201±0.077	0.328±0.157
	BRITS (ref)	10.583±0.123	4.550±0.052	0.268±0.138	7.563±0.151	**6.242±0.069**	0.249±0.118
	CTA (ref)	19.276±0.205	8.285±0.756	1.622±1.245	12.646±0.232	22.069±0.798	1.371±1.429
	STGAE (ref)	9.521±0.159	6.225±0.135	0.417±0.209	16.529±0.378	23.033±0.213	0.496±0.561

Table A.9. Performance metrics for reference baseline models (ref) on data with and without spatio-temporal patterns. Either data are following the MCAR mechanisms without spatio-temporal patterns, or data are induced with spatio-temporal pattern by applying the Markov Chain (MC) simulation model. Results are reported in % with mean±95%CI across time series, features, and validation folds. Best results (smallest metrics) are marked in bold.

"The **Table** displays results from trained reference models (ref), which were all considered non-time causal since they infer statistics from future time points, in comparison to linear interpolation (LINPOL) and BiLSTM autoencoder (LSTM). In scenario **A),** models imputed MCAR patterns, as done by previous authors that claim superiority of DL imputers over linear ones. In scenario **B),** models are applied on more complex and realistic simulated spatio-temporal missingness patterns. For MCAR patterns, LINPOL

yielded lowest globally computed NWSD scores for MIMIC and HIRID. However, more advanced models comprising the reference BRITS and the BILSTAE architecture resulted in lowest CCorD and NRMSE scores across all three datasets. In ICDEP, BILSTAE imputed with best evaluations in comparison to LINPOL as well as all reference models.

Overall, results support findings from the main manuscript, indicating that the simulated missingness patterns from Markov chains pose more complex scenarios for DL imputation methods than simple MCAR inductions lacking in spatio-temporal structure. Apparently, downstream labels hold valuable information for the imputation of time series since BRITS benefited from jointly learning imputations and classifications. Similar claims have been made by benchmarking studies before [5,6]. However, treating these two tasks as independent problems, as reported in the main manuscript, has the advantage of freely selecting any downstream task. Also, since other reference models (SAITS, CTA, STGAE) do not have access to the labels, BRITS evaluations shall be interpreted carefully. The main DL model BILSTAE could prevail over all reference models and showed that we successfully trained an autoencoder model for heterogenous multivariate time series imputations."

[1] Fan, Y., Yu, X., Wieser, R., Meakin, D., Shaton, A., Jaubert, J. N., ... & Wu, Y. (2023). Spatio-temporal denoising graph autoencoders with data augmentation for photovoltaic data imputation. Proceedings of the ACM on Management of Data, 1(1), 1-19.
[2] Cao, W., Wang, D., Li, J., Zhou, H., Li, L., & Li, Y. (2018). Brits: Bidirectional recurrent imputation for time series. Advances in neural information processing systems, 31.
[3] Du, W., Côté, D., & Liu, Y. (2023). Saits: Self-attention-based imputation for time series. *Expert Systems with Applications*, *219*, 119619.
[4] Wi, H., Shin, Y., & Park, N. (2024, March). Continuous-time autoencoders for regular and irregular time series imputation. In Proceedings of the 17th ACM International Conference on Web Search and Data Mining (pp. 826-835).
[5] Wang, X., Zhang, R., Sun, Y., & Qi, J. (2019, May). Doubly robust joint learning for recommendation on data missing not at random. In International Conference on Machine Learning (pp. 6638-6647). PMLR.
[6] Le Morvan, M., Josse, J., Scornet, E., & Varoquaux, G. (2021). What'sa good imputation to predict with missing values?. Advances in Neural Information Processing Systems, 34, 11530-11540.

3. The amplification of MAR/MNAR effects through manually chosen scaling factors (e.g., age- or value-based multipliers) appears somewhat arbitrary. The authors should provide empirical justification (e.g., observed correlations in the raw data) or theoretical grounding based on prior literature.

RESPONSE #3.7: We thank reviewer for the comment and provide theoretical grounding in prior literature for MAR/MNAR effects in **Additional Discussion A.2** as follows:

"When investigating existing effects between covariates and missingness, a general trend of increased missingness was observed among elderly patients in ICDEP. Several studies indicate that low peripheral perfusion, which is more common in older adults, can lead to higher rates of missing values in pulse oximetry signals [1]. Analyses also confirmed that low values of Spo2 < 70%, common in elderly patients, can lead to sensor malfunction and ultimately to data loss [2]. As findings from [3,4] suggest, the post-hoc simulation of MNAR effects bases on these assumptions that less values are available for more extreme levels of clinical parameters. Hence, negative and positive MNAR effects were amplified with no change in general results, while the main manuscript reports imputation results where more unobserved values are present for lower value bins. In MIMIC and HIRID, missing values appeared more frequently in younger intensive care patients. A multi-center study [5] found that older adults (≥ 75 years) were less likely to receive certain interventions (e.g., imaging or drainage) compared to younger patients. Similarly, younger patients (≤ 65 years) made up the largest proportion in a study cohort investigating ventilated patients [6].

Either way, missingness patterns in clinical time series often have multi-factorial reasons which are impossible to tease out retrospectively. Especially MNAR effects cannot be measured in real-world settings due to the reason for a value being missing is never measured. All three datasets MIMIC, HIRID, and ICDEP were also restricted to a selection of routinely collected EHRs, not providing the full cascade of actions resulting in sensor removal or medical interventions (complex inherent MNAR effects). Real-world clinical missingness patterns also depend on monitoring guidelines, the structure of the health-care system, staffing levels, hospital routines, as well as additional patient related factors such as diagnosis, disease progression, and comorbidities [7]. Amplification factors were set so that the altered probabilities for a highly missing state did not exceed 90%, the probabilities for more available states deepening on MAR or MNAR conditions were changed proportionally to have pronounced effects in a controlled environment."

[1] Poorzargar, K., Pham, C., Ariaratnam, J., Lee, K., Parotto, M., Englesakis, M., ... & Nagappa, M. (2022). Accuracy of pulse oximeters in measuring oxygen saturation in patients with poor peripheral perfusion: a systematic review. Journal of clinical monitoring and computing, 36(4), 961-973.
[2] Elgendi, M., Fletcher, R. R., Tomar, H., Allen, J., Ward, R., & Menon, C. (2021). The striking need for age diverse pulse oximeter databases. Frontiers in Medicine, 8, 782422.
[3] Haliduola, H. N., Bretz, F., & Mansmann, U. (2022). Missing data imputation using utility-based regression and sampling approaches. Computer Methods and Programs in Biomedicine, 226, 107172.
[4] Sondhi, A., Weberpals, J., Yerram, P., Jiang, C., Taylor, M., Samant, M., & Cherng, S. (2023). A systematic approach towards missing lab data in electronic health records: A case study in non-small cell lung cancer and multiple myeloma. CPT: Pharmacometrics & Systems Pharmacology, 12(9)
[5] EUROBACT-2 Study Group, & European Society of Intensive Care Medicine. (2024). Presentation, management, and outcomes of older compared to younger adults with hospital-acquired bloodstream infections in the intensive care unit: a multicenter cohort study. Infection.
[6] Ma, J. G., Zhu, B., Jiang, L., Jiang, Q., & Xi, X. M. (2022). Gender-and age-based differences in outcomes of mechanically ventilated ICU patients: a Chinese multicentre retrospective study. BMC anesthesiology, 22(1), 18.
[7] Ayilara, O. F., Zhang, L., Sajobi, T. T., Sawatzky, R., Bohm, E., & Lix, L. M. (2019). Impact of missing data on bias and precision when estimating change in patient-reported outcomes from a clinical registry. Health and quality of life outcomes, 17(1), 106.

4. The main conclusion—that simple linear methods perform robustly under realistic missingness—while practically useful, is not conceptually new. The discussion section should go beyond reporting empirical rankings to offer mechanistic insights into model behavior. For example, why do deep models degrade under structured missingness but excel under MCAR? A deeper analysis would make the study more valuable.

RESPONSE #3.8: We thank the Reviewer #3 for requesting additional analytical aspects for supporting our findings. In the **Discussion**, we added the following:

"Related work by Jäger et al. [1] showed that commonly used imputation methods such as KNN or MICE outperform DL autoencoder on tabular data for complex MNAR scenarios. Benchmarks by Kazijevs et al. [2] found slightly improved performance evaluations for spatio-temporal DL imputers on longitudinal health records but questioned the benefit of these methods over simple linear models due to high computational expenses. Jahangiri et al. [3] imputed systolic and diastolic BP values recommending mean aggregates instead of more complex linear mixed effects models. None of the related studies simulated spatio-temporal missingness patterns for validating imputation model performances.

Similar to Kazijevs et al., baseline analysis with simple MCAR induction showed superiority of DL autoencoders over linear models in these more unrealistic scenarios. We hypothesize that STAEs more easily infer feature values from spatial dependencies when unobserved patterns lack spatio-temporal structure. As an example, systolic BP (bp_sys) can be inferred from diastolic BP (bp_dia) and vice versa

when one of the two features is present, due to a high correlation between these two variables. Specifically, we found Pearson correlation coefficients of 0.55, 0.68 and 0.78 in MIMIC, HIRID, ICDEP between these two features, similar to Jahangiri et al., supporting this hypothesis. When analyzing the local NRMSE bp_sys for the oversimplified MCAR scenario, errors were much lower with 0.630±0.007, 1.819±0.017, and 5.510±0.058 compared to the realistic spatio-temporal scenario that resulted in 9.897±0.112, 10.198±0.098, and 12.564±0.108 for MIMIC, HIRID, and ICDEP. Likewise, all trained reference models, such as BRITS, SAITS or CTA, which learn spatio-temporal structure in clinical time series, yielded more convincing imputation results on simple MCAR inductions. We argue that our simulation model introduces more realistic missingness patterns in clinical time series, such as the joint absence of BP values, leading to complex scenarios which result in diminished performance of STAE autoencoder models."

[1] Jäger, S., Allhorn, A., & Bießmann, F. (2021). A benchmark for data imputation methods. *Frontiers in big Data*, *4*, 693674.
[2] Kazijevs, M., & Samad, M. D. (2023). Deep imputation of missing values in time series health data: A review with benchmarking. *Journal of biomedical informatics*, *144*, 104440.
[3] Jahangiri, M., Kazemnejad, A., Goldfeld, K. S., Daneshpour, M. S., Mostafaei, S., Khalili, D., ... & Akbarzadeh, M. (2023). A wide range of missing imputation approaches in longitudinal data: a simulation study and real data analysis. *BMC Medical Research Methodology*, *23*(1), 161.

5. Generation of complete case datasets (CCD). According to the provided code, in preprocess.py, the function cut_na_sequences() appears to retain only the first complete sequence for each patient, even though a patient may have multiple complete sequences. Please explain the reason for restricting the selection to the first sequence. In addition, considering the CCD generation procedure and Figure A.2 in the Supplement, this choice might result in relatively short sequences, potentially limiting deep learning models' ability to effectively capture temporal dependencies. It could also affect downstream task performance—for example, using early sequences to predict final outcomes may yield poorer results compared to using later sequences. This may partly explain why different methods show limited variation in downstream performance. The construction of the "complete case dataset" (CCD) as ground truth introduces potential selection bias, as it retains only patients with short continuous segments. The authors should discuss how this might affect the generalizability and realism of the results.

RESPONSE #3.9: We now justify the preprocessing of sequences for generating CCD in the **Methods** with "The selection of early available values in multivariate time series allowed the temporal alignment of patient trajectories to the beginning of ICU stays (MIMIC, HIRID) or surgical (ICDEP) intervention across the entire patient cohort (e.g., for applying 1MEAN, 1MEDI). Slicing of different sections of available feature values alongside the temporal dimension did not result in extended sequence lengths. Lastly, the assignment of one unique CCD time series per patient increased the reliability of estimating confidence intervals across independent samples as well as reducing estimation bias when performing train-test splits."

In the **Discussion**, we further add the sentence "While sequence lengths were overall reduced for CCD compared to original data, different strategies for creating complete sets of consecutive available features might have resulted in increased learnable signal for STAE models. However, we showed similar findings across all three datasets that comprised heterogeneously sampled clinical time series, increasing the generalizability of presented findings."

6. The decision to focus on only five vital sign features significantly limits the scope of the benchmark. It would be valuable to test whether the findings hold when including additional intervention variables.

RESPONSE #3.10: We provided a detailed analysis of commonly available features across all three datasets and their availability constraints in the temporal dimension when applying various resampling intervals. In the main manuscript, the selection of five available features expressing low temporal

missingness was justified. Alternative feature sets would have resulted in even shorter sequences with lower temporal dynamics that can be learned. We emphasize this limitation in the **Discussion** with "The presented simulation model is limited to the selection of five highly available vital signs due to strong availability constraints across features and along the time dimension."

7. Computational efficiency is central to benchmarking. The authors should report approximate training times, hardware configurations, and resource requirements for deep models to support reproducibility.

RESPONSE #3.11: We enhanced our **Methods** section with

"For the training of model variants, GPU jobs on a high-performance cluster allocated a maximum of four NVIDIA RTX A400 and were submitted via SLURM. The average computation time during hyperparameter search with RayTune was approx. 7.5 hours for one hyperparameter run per CV fold that optimized STAE architectures. The combined loss for STAE which uses the WSD metric was implement via a Sinkhorn loss. Model versions that were configured with this loss up were tuned for up to 14h due to the increased complexity of calculating this error metric. **Table A.6** in **Supplement A** show extensive ablations of model components that were iteratively added and removed across all three datasets. Hence, it was important to limit the hyperparameter search space as described before for retrieving tuning results within a reasonable time span."

8. The description of the transition matrix is somewhat confusing. In a Markov chain, each row of the transition matrix should sum to 1, representing a valid conditional probability distribution of next-state transitions given the current state. It is unclear whether the authors normalized rows independently or the entire matrix globally. Clarification and consistency checks are needed, as this directly affects the validity of the simulated missingness process.

RESPONSE #3.12: We thank Reviewer #2 for this sharp observation. We calculated occurrence and transition probabilities globally. In the manuscript, we make this point clear and added "The relative frequencies of occurrence and transition states were retrieved and normalized globally for the entire population across all time series." to the **Results** and **Methods** section.

9. The term spatio-temporal may be misleading in this context, as the data under study are multivariate clinical time series without true spatial coordinates. A more accurate term might be "multivariate time series" or "cross-feature time series." The authors should clarify or adjust the terminology accordingly to avoid conceptual confusion.

RESPONSE #3.13: We believe that spatio-temporal aspects as terminologies are well-known by the research community as related authors used these terms to describe their imputation models [1-4]. However, we clarified the usage of our terminology in the **Introduction** as "This study used multivariate time series that were described in the temporal dimension over time and in the spatial dimension across selected features."

[1] Fan, Y., Yu, X., Wieser, R., Meakin, D., Shaton, A., Jaubert, J. N., ... & Wu, Y. (2023). Spatio-temporal denoising graph autoencoders with data augmentation for photovoltaic data imputation. *Proceedings of the ACM on Management of Data*, *1*(1), 1-19.
[2] Jia, Y., Zhou, C., & Motani, M. (2017, November). Spatio-temporal autoencoder for feature learning in patient data with missing observations. In *2017 IEEE International Conference on Bioinformatics and Biomedicine (BIBM)* (pp. 886-890). IEEE.
[3] Li, D., Li, L., Li, X., Ke, Z., & Hu, Q. (2020). Smoothed LSTM-AE: A spatio-temporal deep model for multiple time-series missing imputation. *Neurocomputing*, *411*, 351-363.
[4] Nascimento, M. L., Gonçalves, K. C., & Mendonça, M. J. (2023). Spatio-Temporal Instrumental Variables Regression with Missing Data: A Bayesian Approach. *Computational Economics*, *62*(1), 29-47.

10. Correct minor grammatical and stylistic issues (e.g., “it’s value levels” → “its value levels”).

RESPONSE #3.14: We extensively edited the manuscript file and corrected all grammatical and stylistic issues that occurred.

We thank the Reviewer #2 for this extensive and detailed feedback that led to a substantially improved manuscript. We also updated the GitHub repository with additional documentations for preprocessing and applying our simulation model.

References:
[1] Nie T, Qin G, Ma W, Mei Y, Sun J. ImputeFormer: Low Rankness-Induced Transformers for Generalizable Spatiotemporal Imputation[C/OL]//Proceedings of the 30th ACM SIGKDD Conference on Knowledge Discovery and Data Mining. 2024: 2260-2271[2025-02-27]
[2] Yuan X, Qiao Y. Diffusion-TS: Interpretable Diffusion for General Time Series Generation[C]//The Twelfth International Conference on Learning Representations.
[3] Wu H, Hu T, Liu Y, Zhou H, Wang J, Long M. TimesNet: Temporal 2D-Variation Modeling for General Time Series Analysis[C]//The Eleventh International Conference on Learning Representations.
[4] Wang J, Du W, Cao W, Zhang K, Wang W, Liang Y, Wen Q. Deep Learning for Multivariate Time Series Imputation: A Survey[A/OL]. arXiv, 2024[2024-02-22]. http://arxiv.org/abs/2402.04059.

Response to Reviewers for the manuscript „Benchmarking imputation methods on real-world clinical time series with simulated spatio-temporal missingness“

We thank all the Reviewers for the overall positive feedback. With our current revision of the manuscript, we hope to especially address remaining concerns raised by **Reviewer#1** and **Reviewer#3**. Furthermore, we appreciate the suggestion by **Reviewer#2** to accept our work for publication in Nature Communications.

Editorial Comment:

In particular, for the ethics of this study, please clarify the 'data privacy and sharing regulations' for the MIMIC and HIRID data under the PhysioNet platform.

Studies that were published in Nature Communications by Thakur et al. [1] as well as Skreta et al. [2] used MIMIC-III data and referenced hyperlinks to the Physionet platform under their ‘data privacy and regulations’ sections. Following these examples and further specifying data privacy aspects we enhanced our **Data Availability** and **Code Availability** statement by the following:

“The pre-processed MIMIC and HIRID data are accessible via PhysioNet (https://physionet.org/content/mimiciii/1.4/, https://physionet.org/content/hirid/1.1.1/) where a research proposal must be submitted for HIRID. Permissions for secondary analysis of these databases are granted by the official PhysioNet Credentialed Health Data Use Agreement 1.5.0. MIMIC as well as HIRID comply to the Health Insurance Portability and Accountability Act (HIPAA) by removing protected health information (e.g., names, addresses), shifting timestamps by a patient specific offset, and applying binning techniques to age values. In contrast to MIMIC and HIRID, ICDEP data is not published openly, due to German data privacy regulations, but can be requested from the corresponding author to establish a research collaboration.”

“We share code, trained downstream models, and instructions for the application of our simulation model via GitHub (https://github.com/ngiesa/mi_icu). After data retrieval, this GitHub code can be used to fully reproduce our results.”

[1] Thakur, A., Zhu, T., Abrol, V., Armstrong, J., Wang, Y., & Clifton, D. A. (2024). Data encoding for healthcare data democratization and information leakage prevention. Nature communications, 15(1), 1582.

[2] Skreta, M., Arbabi, A., Wang, J., Drysdale, E., Kelly, J., Singh, D., & Brudno, M. (2021). Automatically disambiguating medical acronyms with ontology-aware deep learning. *Nature communications*, *12*(1), 5319.

In addition, the **Ethics Statement** section was revised with:

“For processing ICDEP data, the IRB EA4/254/21 was granted by the ethics committee at Charité - Universitätsmedizin Berlin. For MIMIC, the IRB at the Beth Israel Deaconess Medical Center granted a waiver of informed consent and approved the data sharing initiative. The IRB of the Canton of Bern approved collection of patient data for the HIRID database. Further legal information can be found at the PhysioNet (https://physionet.org) platform”

Reviewer #1 (Remarks to the Author):

I thank the authors for carefully addressing my previous comments, which has improved the readability of the manuscript. The paper will certainly be of interest to readers of Nature Communications.

Although the authors have responded to my points in their response letter, some critical issues have not yet been fully reflected in the revised manuscript. It is essential that accurate terminology is used when presenting the results; otherwise, the manuscript may convey misleading or incorrect messages to readers. I highlight two important remaining points:

We thank **Reviewer#1** for the positive feedback underscoring that our work will be of interest to readers of Nature Communications. Accurate terminology is a key component for scientific soundness and manuscripts should comprise unambiguous messages. Hence, we extensively revised the usage of terminology related to the MICE imputation approach.

1- As noted in the previous review round and acknowledged by the authors, a multiple imputation (MI) approach has not been applied in this study. As far as I can see, all imputation methods considered (over 20 methods) are single imputation approaches. It is therefore inaccurate to state that MI has been used (e.g., in Table 3). The authors should avoid this terminology throughout the manuscript. In addition, MICE refers to "multivariate imputation using chained equations," and one cycle of the MICE algorithm produces one imputed dataset.

In the Introduction, we now correctly explain the abbreviation MICE with the following:

"Multivariate imputations by chained equations (MICE) iteratively samples from a series of conditional regression models to impute missing values [1]. While the originally proposed algorithm [2] treats observations as independent, some extensions additionally learn from temporal dependencies [3]."

[1] Azur, M. J., Stuart, E. A., Frangakis, C., & Leaf, P. J. (2011). Multiple imputation by chained equations: what is it and how does it work? *International Journal of Methods in Psychiatric Research*, *20*(1), 40–49.

[2] van Buuren, S., & Groothuis-Oudshoorn, K. (2011). mice: Multivariate Imputation by Chained Equations in R. *Journal of Statistical Software*, *45*(3).

[3] Luo, Y., Szolovits, P., Dighe, A. S., & Baron, J. M. (2018). 3D-MICE: integration of cross-sectional and longitudinal imputation for multi-analyte longitudinal clinical data. *Journal of the American Medical Informatics Association*, *25*(6), 645-653.

Multivariate Imputations by Chained Equations					
**Name**	**Imputation Method**	**Cross Population**	**Cross Features**	**Time Causal**	**Static Features**
MICTRE	MICE Random Forest	TRUE	TRUE	FALSE	TRUE
MICKNN	MICE K-Nearest Neighbor	TRUE	TRUE	FALSE	TRUE
MICET	MICE Temporal	TRUE	TRUE	TRUE	TRUE

Table 3 (extract)

We ensure consistent use the terminology "multivariate imputations by chained equations" through the entire manuscript, including **Table 3**.

We agree that we use single imputation methods and do not apply multiple imputations. Hence, we clarify this aspect in the **Methods** section with:

"We use the MICE algorithm to iteratively impute missing values using chained equations, applying a conditional regression model for each partially observed variable, conditioned on the remaining variables in the dataset. At each iteration, imputations are updated sequentially but intermediate imputed datasets were not retained for this study. Rather than performing multiple imputation (MI) resulting in a number of differently imputed dataset, we used the single completed dataset obtained after 40 iterations. Hence, all our methods displayed in **Table 3** are single imputation approaches. Downstream tasks were also not compared across different MI imputed sets, hence Rubin's rule for pooling results was not applied in our experimental setup."

2- Similarly, while the authors confirmed that MICE can learn from temporal correlations (a variant they called MICET), Table 3 still states that MICE cannot condition on previous recordings of a feature. This is incorrect and should be revised accordingly throughout the manuscript.

We totally agree with **Reviewer#2** that MICET, a temporal extension of MICE, is explicitly conditioned on previous recordings. Hence, this model version can be considered as time causal. In the **Methods**, we write the following:

"In addition to the standard MICE method, we also evaluated an enhanced temporal version, denoted as MICET, where the input space is conditioned on previously measured records for each feature. Due to this condition, MICET can be considered as time causal."

We updated **Table 3** and now integrate MICET into the descriptions of used imputation methods. Besides, we now report the imputation performances of MICET in the main manuscript instead of presenting these results in the **Appendix A** only.

	MIMIC			HIRID			ICDEP		
	CCorE	NRMSE	NWSD	CCorE	NRMSE	NWSD	CCorE	NRMSE	NWSD
Time-Causal Methods									
1MEAN	9.852±0.195	16.695±0.139	0.541±0.112	4.719±0.092	26.046±0.276	0.333±0.281	9.064±0.153	17.423±0.133	0.491±0.242
1MEDI	9.696±0.192	16.333±0.134	0.535±0.108	4.618±0.093	25.769±0.277	0.338±0.285	8.735±0.149	16.783±0.131	0.472±0.228
RMEAN	**6.663±0.162**	11.798±0.104	0.131±0.023	3.370±0.079	19.542±0.233	0.116±0.112	7.945±0.152	14.870±0.134	0.210±0.075
RMEDI	6.692±0.162	11.873±0.107	0.117±0.020	3.617±0.082	20.406±0.256	0.141±0.144	7.970±0.153	14.815±0.136	0.196±0.067
MICET	12.739±0.211	**9.283±0.081**	**0.032±0.010**	6.600±0.108	17.280±0.242	0.160±0.172	12.371±0.184	**11.825±0.130**	**0.051±0.020**
LOCF	7.041±0.164	11.862±0.114	0.062±0.012	**3.164±0.081**	**17.080±0.242**	**0.010±0.008**	**7.764±0.154**	14.247±0.139	0.161±0.050
LSTAE	7.107±0.168	11.775±0.105	0.265±0.045	4.588±0.096	23.791±0.317	0.177±0.168	8.553±0.161	13.877±0.122	0.319±0.146

Table 2 (extract)

In our revised manuscript, **Table 2** presents imputation performance metrics obtained by MICET in addition to other time-causal methods (see extract from **Table 2**). MICET achieved good performance results with respect to NRMSE and NWSD for MIMIC as well ICDEP data. Hence, we highlight these results in the **Imputation Benchmark** section with:

"The autoencoder LSTAE resulted in smaller NRMSE in contrast to LOCF for MIMIC (NRMSE=11.775±0.105) as well as ICDEP (NRMSE=13.877±0.122). However, MICET that was implemented as a time-causal version of MICE resulted in significantly lower error margins for these two datasets (NRMSE=9.283±0.081, 11.825±0.130 in MIMIC, ICDEP)."

Furthermore, we added the following paragraph to the **Discussion** section:

"MICE imputations estimated global distributions of vital signs within our study population closest to GT data. MICET that was conditioned on previous observations led to improved local and distributional errors compared with DL methods, particularly on MIMIC and ICDEP data."

In **Supplement A**, performance metrics for MICET under MAR and MNAR amplification scenarios have been added accordingly.

We thank **Reviewer#1** for emphasizing the capabilities of MICE to condition on previously recorded values. Based on the proposed method, we could drastically improve the creditability of our **Results** and **Discussion**.

Reviewer #2 (Remarks to the Author):

This revised manuscript presents a timely and practically relevant benchmark for imputation in clinical time series. The paper introduces a Markov chain-based simulator to generate realistic spatio-temporal missingness patterns from real clinical data and evaluates a broad range of imputation methods across three datasets. The study addresses an important gap in the literature, since many previous benchmarks rely on oversimplified MCAR-style masking and therefore may overestimate the benefit of complex deep learning imputers. In contrast, this work demonstrates that under more realistic structured missingness, simple approaches such as linear interpolation and LOCF remain highly competitive and often outperform more sophisticated spatio-temporal autoencoder models, while downstream prediction performance differences across imputers are generally modest.

This revised manuscript presents a timely and practically relevant benchmark for imputation in clinical time series.

I am very satisfied with the revised manuscript. After revision, my previous concerns are largely resolved. I therefore support publication of this work.

We thank **Reviewer#2** for the positive response. We are delighted that all comments have been addressed leading to the suggestion to accept our work for publication.

Reviewer #3 (Remarks to the Author):

Overall, the authors have provided clear responses to all comments raised by Reviewer #3, and most of the suggested revisions have been properly incorporated into the main manuscript and the Supplementary Materials. The quality of the manuscript has improved accordingly. However, a few minor issues remain and could be further refined.

We thank the **Reviewer#3** for all previous comments that resulted into an improved manuscript, including various ablations. However, we now provide additional experiments with different scaling factors for MNAR and MAR amplification scenarios as suggested.

1. In the response to Weak point 3, the referenced figure should be Figure A.3 rather than Figure A.4. This appears to be a minor typographical error and does not affect the overall results.

We thank the reviewer for this sharp observation. In the last response to **Reviewer#2**, we included a figure showing differences of occurrence states between simulated and original data across age bins as well as datasets. In the response, this figure was falsely named as **Figure A.4** while in our submitted **Supplement A**, the same figure appears as **Figure A.3.** We ensured consistent naming of figures in the main manuscript and in the supplement.

2. Regarding Detailed Comment 3, the design of MAR and MNAR amplification relies on scaling factors and thresholds (e.g., the 90% discussed in Additional Discussion A.2), which appear somewhat heuristic. While this is understandable given the complexity of real-world clinical missingness, it remains unclear how sensitive the results are to these choices. In the current manuscript, the experiments seem to rely on a single configuration of these scaling parameters. It would strengthen the work if the authors could provide a sensitivity analysis with respect to different scaling factors (or threshold values), at least for a subset of representative imputation methods. This would help assess the robustness of the reported conclusions under varying missingness intensities.

We thank **Reviewer#3** for suggesting additional experiments, evaluating the robustness of our findings under varying scaling factors for MAR and MNAR conditions. We agree that, given the complexity of real-world missingness patterns, we cannot fully specify these factors deterministically. Thus, we provide further heuristic sensitivity analyses using different combinations of scaling factors.

Previous experiments have already applied a reversed MNAR effect (with a negative effect direction; lower GT value levels = higher missingness), but results were left out to reduce the complexity of our reported results (a positive MNAR effect direction is used in the main manuscript). Initially, scaling factors were set in an explorative manner to drastically increase MNAR as well as MAR relations. To weaken these amplifications, additional experiments were conducted for this revision, using a set of smaller scaling factors. Specifically, we now report following three scenarios in **Supplement A.**

1. **MNAR reverse:** Instead of using scaling factors of {1.8, 2.8, 3.8}, reported in the main manuscript, factors of {3.4, 2.4, 1.4} were applied for a reverse (negative) MNAR effect.
2. **MNAR low:** To decrease the intensity of MNAR amplifications, additional scaling factors of {1, 1.5, 2} were used, being smaller than the originally reported {1.8, 2.8, 3.8}
3. **MAR low:** To decrease the intensity of MAR amplifications, factors of {1, 1.1, 1.2} were applied instead of {1.2, 2.2, 3.2} that were reported in the main manuscript

Amplification	Method	CCorE	NRMSE	NWSD
		**MIMIC**		
MNAR reverse	LINPOL	**15.381±0.184**	**4.331±0.054**	**0.191±0.047**
	BILSTAE	21.324±0.201	6.357±0.060	0.878±0.172
	BILSTAE_STAT	20.853±0.199	6.083±0.058	0.761±0.138
	LOCF	**16.072±0.179**	**4.877±0.060**	**0.109±0.028**
	LSTAE	22.126±0.203	6.698±0.063	0.890±0.168
	LSTAE_STAT	22.073±0.201	6.721±0.063	0.935±0.149
MNAR low	LINPOL	**14.553±0.180**	**5.119±0.043**	**0.171±0.029**
	BILSTAE	14.906±0.180	5.369±0.042	0.452±0.074
	BILSTAE_STAT	14.734±0.178	5.292±0.041	0.446±0.075
	LOCF	**15.251±0.177**	5.784±0.047	**0.100±0.021**
	LSTAE	15.671±0.183	5.758±0.044	0.542±0.093
	LSTAE_STAT	15.460±0.182	**5.694±0.044**	0.522±0.090
MAR low	LINPOL	**12.267±0.212**	**4.924±0.048**	**0.199±0.035**
	BILSTAE	20.603±0.300	6.268±0.063	0.990±0.169
	BILSTAE_STAT	20.509±0.300	6.195±0.062	0.958±0.174
	LOCF	**13.003±0.212**	**5.538±0.053**	**0.128±0.026**
	LSTAE	21.236±0.300	6.676±0.065	0.923±0.198
	LSTAE_STAT	21.054±0.299	6.614±0.065	0.901±0.194
		**HIRID**		
MNAR reverse	LINPOL	**5.933±0.113**	**8.145±0.170**	**0.055±0.051**
	BILSTAE	8.834±0.121	11.346±0.202	0.448±0.433
	BILSTAE_STAT	8.352±0.118	11.321±0.203	0.460±0.444
	LOCF	**5.488±0.102**	**7.842±0.151**	**0.017±0.016**
	LSTAE	8.408±0.122	10.602±0.186	0.413±0.377
	LSTAE_STAT	8.223±0.120	10.643±0.190	0.438±0.399
MNAR low	LINPOL	**5.305±0.098**	**8.264±0.133**	**0.017±0.016**
	BILSTAE	7.668±0.108	12.777±0.217	0.553±0.564
	BILSTAE_STAT	7.383±0.105	12.283±0.208	0.552±0.560
	LOCF	**5.615±0.105**	**8.555±0.152**	**0.053±0.049**
	LSTAE	8.759±0.120	13.285±0.222	0.526±0.529
	LSTAE_STAT	7.840±0.110	12.486±0.209	0.547±0.541
MAR low	LINPOL	6.763±0.125	8.620±0.127	0.021±0.012
	BILSTAE	9.543±0.173	11.549±0.205	0.794±0.545
	BILSTAE_STAT	9.043±0.122	10.196±0.205	0.689±0.522
	LOCF	**6.898±0.134**	**8.954±0.156**	**0.064±0.039**
	LSTAE	9.294±0.181	11.465±0.216	0.815±0.531
	LSTAE_STAT	9.047±0.180	11.414±0.216	0.800±0.541
		**ICDEP**		
MNAR reverse	LINPOL	**11.483±0.172**	**4.131±0.077**	**0.160±0.075**
	BILSTAE	19.611±0.175	8.483±0.096	1.040±0.545
	BILSTAE_STAT	19.375±0.173	8.510±0.095	1.071±0.554
	LOCF	**11.938±0.170**	**4.737±0.086**	**0.207±0.108**
	LSTAE	23.471±0.175	8.553±0.096	1.016±0.504
	LSTAE_STAT	24.535±0.172	8.928±0.098	1.231±0.605
MNAR low	LINPOL	18.391±0.183	7.584±0.070	**0.247±0.118**
	BILSTAE	17.576±0.160	7.461±0.062	0.601±0.292
	BILSTAE_STAT	**17.259±0.159**	**7.388±0.062**	0.584±0.283
	LOCF	18.965±0.174	8.745±0.079	**0.324±0.166**
	LSTAE	18.130±0.162	7.862±0.064	0.683±0.323
	LSTAE_STAT	**17.807±0.160**	**7.813±0.064**	0.661±0.312
MAR low	LINPOL	**12.787±0.217**	**5.655±0.064**	**0.278±0.067**
	BILSTAE	19.327±0.272	6.987±0.068	1.014±0.252
	BILSTAE_STAT	19.300±0.273	6.965±0.068	1.006±0.250
	LOCF	**12.256±0.218**	**6.397±0.070**	**0.312±0.092**
	LSTAE	19.734±0.277	7.251±0.069	1.103±0.270
	LSTAE_STAT	19.918±0.285	7.246±0.069	1.083±0.269

Table A.11

Table A.11 presents the locally applied CCorE, NRSME, and the globally computed NWSD across amplification scenarios and imputation methods. Linear interpolation (LINPOL) and BiLSTM autoencoders that learn either with (BILSTAE_STAT) or without (BILSTAE) static attributes are presented as non time-causal methods. Time-causal methods comprise LOCF and LSTM autoencoders with (LSTAE_STAT) and without (LSTAE) static attributes. Performance evaluations in **Table A.11** are in line with our main findings, suggesting that linear imputation methods are robust imputers even when MAR and MNAR conditions are amplified under varying missing intensities. Specifically, we added **Additional Results A.4** to the **Supplement A**:

"We assessed the sensitivity of imputation methods under varying missing intensities for simulation scenarios with amplified MAR and MNAR effects. For additionally evaluating MNAR effects in the reverse direction, scaling factors of {1.8, 2.8, 3.8} were used in the **MNAR reverse** setting. The main manuscript reports experiments using large shifts in missing rates with respect to age-bins for MAR as well as to value-bins for MNAR amplifications. Hence, the scenarios **MAR low** as well as **MNAR low** were constructed using smaller scaling factors of {1, 1.5, 2} and {1, 1.1, 1.2}, respectively. **Table A.11** displays sensitivity results with respect to six imputation methods evaluated with three performance metrics across amplification scenarios and databases. As a result, linear imputation methods were evaluated as robust imputer in contrast to DL autoencoders, supporting the findings of the main manuscript."

3. Regarding Detailed Comment 8, there appears to be an inconsistency in the description of the transition probabilities. On page 5, the manuscript states that "The relative frequencies of occurrence and transition states were retrieved and normalized globally for the entire population across all time series," which is consistent with the presentation in Figure 6(d). However, in the Simulation Model section on page 15, it is stated that the transition probabilities are defined "with rows summing up to one (see Figure 6d)." This creates ambiguity regarding how the transition probabilities are actually computed. I recommend that the authors clarify this point by providing a formal definition or equation, and explicitly explain why a standard row-normalized transition matrix (i.e., each row summing to one) is or is not used.

We thank **Reviewer#3** for requesting specifications of how we normalized the transition matrix to eventually generate transition probabilities from which our Markov model samples.

Under the section **Missingness Simulation** we now specify the following:

"The absolute frequencies of occurrence states were retrieved and normalized globally for the entire population across all time series per dataset, resulting in a set of occurrence probabilities. The absolute counts of transitions from each single state to all other possible states were retrieved and normalized so that resulting relative frequencies, which translate to transition probabilities, sum up to one. Consequently, the Markov chain model uses these normalized transition probabilities to sample the next consecutive state (at t+1) for the selected state (at t)."

The ambiguity of the describing paragraph and the panel in **Figure 6d** arises from the illustration of a minimal example (see blue colorization in panels **a, b, c**) in contrast to the actual global normalization (in addition to the blue missing matrix, the red and all others must be considered for N patients per dataset). To clarify this point, we enhance the caption for **Figure 6** with **"**In panel **d**, the absolute counts of transitions are inserted into a transition table for the observed state space in **c.** In contrast to this minimal example, we retrieved all absolute counts of transitions covered by the entire population and normalized them across all patient time series per dataset so that rows sum up to one."

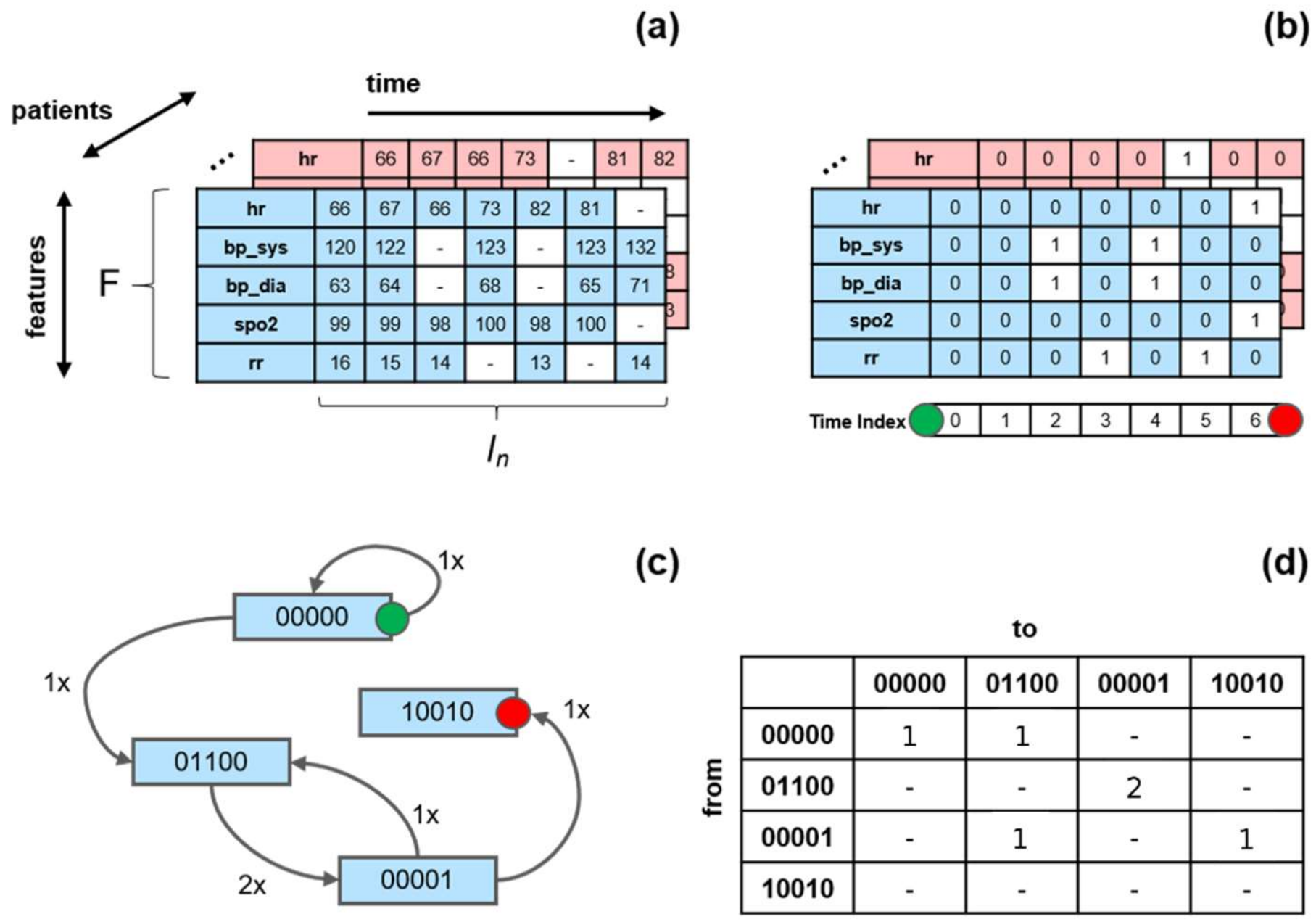

Figure 6

We thank **Reviwer#3** for revising our manuscript and the supplementary files in detail with great precision and high accuracy. We hope to resolve all points that have been raised with this current version of our manuscript.

We thank Reviewer #1 and Reviewer #2 to fully support the publication of our manuscript without further comments. We addressed the remaining minor editorial inconsistency which was raised by Reviewer #3.

Reviewer #3: I only note one minor editorial inconsistency in the rebuttal: the MNAR reverse setting is first described as using scaling factors of {3.4, 2.4, 1.4}, whereas the later quoted text for Additional Results A.4 states that scaling factors of {1.8, 2.8, 3.8} were used for the MNAR reverse setting.

We now clarify this minor inconsistency in **Additional Results A.4** with "For additionally evaluating MNAR effects in the reverse direction, scaling factors of {3.4, 2.4, 1.4} were used in the setting." to be consistent.
