# Supplementary material for: Benchmarking imputation methods on real-world clinical time series with simulated spatio-temporal missingness
Source: Nat Commun. 2026 Oct 5;17:10491. doi: 10.1038/s41467-026-77303-8 (PMC13639038; doi:10.1038/s41467-026-77303-8)
Supplement: Supplementary file 3 [file 41467_2026_77303_MOESM3_ESM.pdf]

nature portfolio

Corresponding author(s): Niklas Giesa
Last updated by author(s): Jul 15, 2026

Reporting Summary

Nature Portfolio wishes to improve the reproducibility of the work that we publish. This form provides structure for consistency and transparency in reporting. For further information on Nature Portfolio policies, see our Editorial Policies and the Editorial Policy Checklist.

Statistics

For all statistical analyses, confirm that the following items are present in the figure legend, table legend, main text, or Methods section.

n/a	Confirmed	
☐	☒	The exact sample size (*n*) for each experimental group/condition, given as a discrete number and unit of measurement
☐	☒	A statement on whether measurements were taken from distinct samples or whether the same sample was measured repeatedly
☒	☐	The statistical test(s) used AND whether they are one- or two-sided<br>*Only common tests should be described solely by name; describe more complex techniques in the Methods section.*
☐	☒	A description of all covariates tested
☐	☒	A description of any assumptions or corrections, such as tests of normality and adjustment for multiple comparisons
☐	☒	A full description of the statistical parameters including central tendency (e.g. means) or other basic estimates (e.g. regression coefficient) AND variation (e.g. standard deviation) or associated estimates of uncertainty (e.g. confidence intervals)
☒	☐	For null hypothesis testing, the test statistic (e.g. *F*, *t*, *r*) with confidence intervals, effect sizes, degrees of freedom and *P* value noted<br>*Give P values as exact values whenever suitable.*
☒	☐	For Bayesian analysis, information on the choice of priors and Markov chain Monte Carlo settings
☒	☐	For hierarchical and complex designs, identification of the appropriate level for tests and full reporting of outcomes
☐	☒	Estimates of effect sizes (e.g. Cohen's *d*, Pearson's *r*), indicating how they were calculated

Our web collection on statistics for biologists contains articles on many of the points above.

Software and code

Policy information about availability of computer code

Data collection	MIMIC and HIRID data was retreived via the PhysioNet platform. For preprocessing MIMIC, the package MIMIC-Extract was used where resulting data is hosted by PhysioNet. For ICDEP, data was extracted from a central data warehouse infrastructure at Charité. After validation, specific data for patients that suffered from postoperative delirium were selected for this database. Python scripts were used for preprocessing residing on the local data instance. See requirements.txt on https://github.com/ngiesa/mi_icu (10.5281/zenodo.21356282).
Data analysis	Data were analyzed with the following Python 3.8 packages: numpy 1.24.4, statsmodels 0.13.5, pandas 2.0.3, sklearn 0.0.post1, and pytorch lightning 1.6.5.post0, torch 2.4.1, autograd 1.5. To install the exact versions, please use the provided requirements.txt (https://github.com/ngiesa/mi_icu).

For manuscripts utilizing custom algorithms or software that are central to the research but not yet described in published literature, software must be made available to editors and reviewers. We strongly encourage code deposition in a community repository (e.g. GitHub). See the Nature Portfolio guidelines for submitting code & software for further information.

Data

Policy information about availability of data

All manuscripts must include a data availability statement. This statement should provide the following information, where applicable:

- Accession codes, unique identifiers, or web links for publicly available datasets
- A description of any restrictions on data availability
- For clinical datasets or third party data, please ensure that the statement adheres to our policy

The pre-processed MIMIC-III v1.4 and HIRID v1.1.1 data are available under restricted access for data privacy reasons, access can be obtained by PhysioNet (https://physionet.org/content/mimiciii/1.4/, https://physionet.org/content/hirid/1.1.1/). After completing an ethics course at the PhysioNet platform, MIMIC and HIRID are accessible, requiring the submission of a research proposal. Permissions for secondary analysis of these databases are granted by the official PhysioNet Credentialed Health Data Use Agreement 1.5.0. MIMIC as well as HIRID comply to the Health Insurance Portability and Accountability Act (HIPAA) by removing protected health information (e.g., names, addresses), shifting timestamps by a patient specific offset, and applying binning techniques to age values. In contrast to MIMIC and HIRID, raw ICDEP data are protected and are not available due to data privacy laws. Here, we cannot fully mitigate all de-identification risks, violating fundamental data privacy rights according to German data privacy regulations and the GDPR. However, pseudonymous ICDEP data can be requested from the corresponding author via niklas.giesa@charite.de. We require a detailed project description of how ICDEP data are going to be used and processed. The expected time frame for responses to access requests is two weeks. If data access has been granted, data will be available for the duration of the project for a maximum of three months. Source data for Figure 2b, Figure 3b, and Figure 4a are provided with this paper.

Research involving human participants, their data, or biological material

Policy information about studies with human participants or human data. See also policy information about sex, gender (identity/presentation), and sexual orientation and race, ethnicity and racism.

Reporting on sex and gender	Through the entire manuscript, we used sex and not gender since this attribute was assigned biologically by the clinical IT systems . These systems were used to extract the data. Currently, there is no data available on gender, defined by the social individual context. For the in-hospital dataset ICDEP, more descriptive fields were integrated into the IT systems after the data extraction (since 2023 available). Hence, we cannot additionally report these data items.
Reporting on race, ethnicity, or other socially relevant groupings	Race or ethnicity are not used as proxies for the socioeconomical status. These properties are not reported for ICDEP since they are not avaiable. For HIRID, these data are restriced to biological sex and some insurence context. For MIMIC, ethnicity is reported in the original published data. We did not actievly controlled for these variables but rather report changes in sex and mortality distributions for our used complete case data. Missingness patterns were only corrected for the confounder of age. Here we found a positive correlation between elderly patients and the fraction of unobserved variables.
Population characteristics	For MIMIC, we included N=34,472 patients of age 64.1±17.68 with a sex distribution of 43.47% F / 56.53% M and a prevalence of in-hospital death of 9%. For HIRID we included N=36,098 patients of age 63.52±15.20 with a sex distribution of 35.80% F / 64.20% M and a prevalence of in-hospital death of 6%. For MIMIC, we included N=34,472 patients of age 64.1 ±17.68 with a sex distribution of 43.47% F / 56.53% M and a prevalence of in-hospital death of 9%. For ICDEP we included N=41,832 patients of age 54.25±18.11 with a sex distribution of 49.76% F / 50.24% M and a prevalence of postoperative delirium of 9%.
Recruitment	There was no specific patient recruiting process due to the retrospective inclusion of electronic health records.
Ethics oversight	For processing ICDEP data, the IRB EA4/254/21 was granted by the ethics committee at Charité - Universitätsmedizin Berlin (chair: Prof. Dr. med. R. Morgenstern). For MIMIC, the IRB at the Beth Israel Deaconess Medical Center granted a waiver ofinformed consent and approved the data sharing initiative. The IRB of the Canton of Bern approved collection of patient datafor the HIRID database. Further legal information can be found at the PhysioNet (https://physionet.org) platform.

Note that full information on the approval of the study protocol must also be provided in the manuscript.

Field-specific reporting

Please select the one below that is the best fit for your research. If you are not sure, read the appropriate sections before making your selection.

☒ Life sciences ☐ Behavioural & social sciences ☐ Ecological, evolutionary & environmental sciences

For a reference copy of the document with all sections, see nature.com/documents/nr-reporting-summary-flat.pdf

Life sciences study design

All studies must disclose on these points even when the disclosure is negative.

Sample size	After using the data extraction pipelines for each dataset, 34,472, 36,098, and 41,832 patients were comprised by the MIMIC, HIRID, and ICDEP dataset. Reducing the clinical time series to complete case data resulted in the inclusion of 14,286, 11,286, and 17,811 subjects for MIMIC, HIRID, ICDEP. No explicit formula has been used to determine these sample sizes as they purely relied on

	removing patient data exhibiting missing values. Furthermore, all experiments were conducted with 5 selected features, splitting data into 3 separate train / validation folds. Here, 66.6% were used for training, 33.3% data were used for testing
Data exclusions	For ICDEP: Data exclusions were made as follows: Adult patients admitted 2017-2020 (116,534 patients, 150,174 hospital stays, 172,624 surgeries) -> Non-cardiac and non-craniotomy surgeries (170,040 surgeries), Anesthesia between 0.5 and 12h (168,032 surgeries), Surgeries with recover room admission (96,715 surgeries) -> 72,100 surgeries with Nu-DESC evaluation
Replication	We wrote reusable code and published our best performing model variants to replicate our findings. We also estimated uncertainty on a bootstrapped and resampled testing set using confidence intervals for estmations.
Randomization	We randomly divided our data into train and testing sets. Prediction models have learned with all covariates that were included as model predictors.
Blinding	Blinding does not apply directly due to the extraction of electronic health records. We have not seen any patients physically that were included into our study. The bias of any relation between providing health care and performing this study can be seen as non-existent.

Reporting for specific materials, systems and methods

We require information from authors about some types of materials, experimental systems and methods used in many studies. Here, indicate whether each material, system or method listed is relevant to your study. If you are not sure if a list item applies to your research, read the appropriate section before selecting a response.

Materials & experimental systems

n/a	Involved in the study
☒	☐ Antibodies
☒	☐ Eukaryotic cell lines
☒	☐ Palaeontology and archaeology
☒	☐ Animals and other organisms
☐	☒ Clinical data
☒	☐ Dual use research of concern
☒	☐ Plants

Methods

n/a	Involved in the study
☒	☐ ChIP-seq
☒	☐ Flow cytometry
☒	☐ MRI-based neuroimaging

Clinical data

Policy information about clinical studies

All manuscripts should comply with the ICMJE guidelines for publication of clinical research and a completed CONSORT checklist must be included with all submissions.

Clinical trial registration	We did not performed a clinical trial. This work was approved by the local institutional review board of the Charité - Universitätsmedizin Berlin under the internal ethics approval number EA4/254/21
Study protocol	The study protocol does not apply for our work relying on retropective health records.
Data collection	MIMIC holds data collected between 2008 and 2014 in the US. HIRID is a Swiss database storing recordings from 2008 to June 2016. For ICDEP, data was collected in our clinical information systems between 2017 and 2020 at three hospital sides.
Outcomes	In-hospital death for MIMIC and HIRID, postoperative delirium measured with the Nu-DESC score for ICDEP.

Plants

Seed stocks	n/a
Novel plant genotypes	n/a
Authentication	n/a
